# Parameter Estimation from Phylogenetic Trees Using Neural Networks and Ensemble Learning

**DOI:** 10.1093/sysbio/syaf060

**Published:** 2025-09-03

**Authors:** Tianjian Qin, Koen J van Benthem, Luis Valente, Rampal S Etienne

**Affiliations:** Groningen Institute for Evolutionary Life Sciences, University of Groningen, Nijenborgh 7, Groningen, 9747 AG, The Netherlands; Groningen Institute for Evolutionary Life Sciences, University of Groningen, Nijenborgh 7, Groningen, 9747 AG, The Netherlands; Groningen Institute for Evolutionary Life Sciences, University of Groningen, Nijenborgh 7, Groningen, 9747 AG, The Netherlands; Naturalis Biodiversity Center, Darwinweg 2, Leiden, 2333 CR, The Netherlands; Groningen Institute for Evolutionary Life Sciences, University of Groningen, Nijenborgh 7, Groningen, 9747 AG, The Netherlands

**Keywords:** Graph neural network, machine learning, recurrent neural network, regression

## Abstract

Species diversification is characterized by speciation and extinction, the rates of which can, under some assumptions, be estimated from time-calibrated phylogenies. However, maximum likelihood estimation methods (MLE) for inferring rates are limited to simpler models and can show bias, particularly in small phylogenies. Likelihood-free methods to estimate parameters of diversification models using deep learning have started to emerge, but how robust neural network methods are at handling the intricate nature of phylogenetic data remains an open question. Here we present a new ensemble neural network approach to estimate diversification parameters from phylogenetic trees that leverages different classes of neural networks (dense neural network, graph neural network, and long short-term memory recurrent network) and simultaneously learns from graph representations of phylogenies, their branching times, and their summary statistics. Our best-performing ensemble neural network (which adjusts the graph neural network result using a recurrent neural network) delivers estimates faster than MLE and shows less sensitivity to tree size for constant-rate and diversity-dependent speciation scenarios. It performs well compared with an existing convolutional network approach. However, like MLE, our approach still fails to recover parameters precisely under a protracted birth-death process. Our analysis suggests that the primary limitation to accurate parameter estimation is the amount of information contained within a phylogeny, as indicated by its size and the strength of effects shaping it. In cases where MLE is unavailable, our neural network method provides a promising alternative for estimating phylogenetic tree parameters. If detectable phylogenetic signals are present, our approach delivers results that are comparable to MLE but without inherent biases.

Identifying the underlying mechanisms shaping biodiversity is an important goal in the fields of evolutionary biology and ecology. Species diversification processes can often be characterized by speciation and extinction rates, which can be estimated from time-calibrated phylogenies (Nee et al. 1997) as long as the assumed model structure of diversification resembles the true underlying data generation process (Louca and Pennell 2021). Time-calibrated phylogenies contain branching times and evolutionary relationships (captured in the topology) between species and offer a complementary source of information to the often incomplete fossil record (Kidwell and Flessa 1996). The increasing availability of reconstructed phylogenies has empowered many studies seeking explanations for the underlying diversity patterns using modelling approaches (Wagner 2000; Morlon 2014; Etienne et al. 2016). One type of models—birth-death (BD) models—are often used to estimate speciation, extinction, and diversification rates from reconstructed phylogenetic trees (Hey 1992; Nee et al. 1994, 1997; Nee 2001).

Likelihood-based approaches, such as maximum likelihood estimation (MLE) and Bayesian inference, can be used to infer not only speciation and extinction rates but also possibly existing evolutionary and ecological signals, such as diversity-dependence or trait-dependence of rates, from branching times and other information sources (Maddison et al. 2007; Etienne et al. 2012, 2014; Valente et al. 2015; Alexander et al. 2016). However, Etienne et al. (2016) showed that MLE estimates of the clade-level carrying capacity in diversity-dependent diversification (DDD) models tend to be close to (but evidently higher than) the number of species observed in the phylogeny, which also biases the other parameters.

An alternative to these likelihood-based approaches for parameter estimation is approximate Bayesian computation (ABC), which approximates the posterior distribution of parameters without requiring explicit calculation of a likelihood. ABC is often seen as a good substitute to MLE when the likelihood function of a model is not available, as long as simulations of the model are fast and tractable (Beaumont et al. 2002; Beaumont 2010; Janzen et al. 2015). However, studies using ABC for parameter estimation in phylogenetics remain scarce (Rabosky 2009; Bokma 2010; Kutsukake and Innan 2013; Xie et al. 2023). This is partly due to the fact that it is often difficult to identify adequate summary statistics and potential distance metrics in ABC, which makes the application and development of this potentially powerful approach challenging.

A promising class of tools that may help overcome the limitations of likelihood-based methods and ABC are machine learning approaches, such as neural networks. Classic feed-forward neural networks comprise layers of nodes, or “neurons,” which process input data and learn to recognize patterns between input and output data from training data (Aggarwal 2018). They have achieved good results in tasks such as image recognition and natural language processing (Zhu et al. 2018). Another class of neural networks, graph neural networks (GNNs), are designed specifically for graph-structured data, such as social networks, molecular structures, and ecological interaction networks. They can capture the dependencies and relationships inherent in data types that can be naturally represented as graphs (Kipf and Welling 2016) and have shown strong performance in various tasks involving graph representation learning (Ying et al. 2018; Li et al. 2020; Rampášek et al. 2022). Phylogenetic trees can also be viewed as graphs, suggesting that GNNs have potential applicability in phylogenetics. Recurrent neural networks, another type of neural network, are designed to handle sequential data, such as time-series, by maintaining a memory of previous inputs (Sak et al. 2014; Salehinejad et al. 2017). Recurrent neural networks can process inputs of varying lengths and capture time-dependent features, making them particularly well-suited for tasks where the order of data points is crucial, such as learning parameters from branching times when viewed as a time-series.

Owing to the rapid development of both hardware capability and deep learning algorithms, applications of neural networks in phylogenetic analyses have started to emerge (Dopazo and Carazo 1997; Tao et al. 2019; Reiman et al. 2020; Suvorov et al. 2020; Zou et al. 2020; Moi and Dessimoz 2022; Lajaaiti et al. 2023; Lambert et al. 2023; Suvorov and Schrider 2024). For instance, phylogenetic deep learning approaches have been shown to provide reliable estimates of parameters in epidemiological, BD, and trait-dependent speciation models (Voznica et al. 2022; Lajaaiti et al. 2023; Lambert et al. 2023; Thompson et al. 2024). Despite their potential, employing neural networks for estimating parameters based on the whole phylogenetic tree, especially those associated with diversification, poses significant challenges and requires further systematic research regarding their performance, accuracy, and robustness. Specifically, feed-forward linear neural networks usually require a large amount of data to be able to generalize well on the patterns within the data (Zhu et al. 2018); producing graph representations for graph-level learning can be challenging given the need to aggregate information across diverse graph sizes and topologies (Ying et al. 2018); the ability of the recurrent neural networks to predict parameters from whole sequences is often challenging (Sak et al. 2014). Hence, how robust neural network methods are at handling the intricate nature of phylogenetic data remains an open question.

In this study, we explore the potential of neural networks in research on species diversification using phylogenies. We first develop various neural network architectures and protocols for transforming phylogenetic trees and branching times into compatible formats. In addition to new neural networks presented here, for comparison with existing methods, we also investigate the one-dimensional convolutional neural network architecture described by Lajaaiti et al. (2023) and Voznica et al. (2022). We then investigate accuracy of parameter estimation on simulated data for ensemble learning strategies. These strategies combine different neural network classes to maximize data utilization and enhance performance. We also assess the determinants of estimation accuracy and robustness for both neural network and MLE methods under various diversification scenarios. Finally, we apply our trained neural networks on empirical phylogenetic data sets and compare their estimates with those of MLE.

Our analyses encompass three different diversification scenarios for which likelihood-based inference approaches already exist: a constant-rate BD scenario, with constant speciation and extinction rates over time (Stadler 2011); a DDD scenario, where the number of species in a clade negatively affects the speciation rate (Etienne et al. 2012); and a protracted birth-death (PBD) scenario, where speciation takes time and does not always proceed to completion (Rosindell et al. 2010). Our findings indicate that neural network approaches are as effective, if not more so, than MLE in recovering parameters from phylogenetic data simulated under a broad range of the parameter space. Trained neural networks can be conveniently applied to empirical trees for parameter estimation. To facilitate this, we present an exemplary R package, “EvoNN,” to illustrate such analyses based on phylogenetic trees (empirical or simulated) supplied by the user (Qin 2024).

## Materials and Methods

### Software Environment and Computational Budget

We used a hybrid programming environment with PyTorch 1.12.1 (Imambi et al. 2021), PyTorch Geometric 2.3.1 (Fey and Lenssen 2019), Python 3.7.1 (Python 2021), CUDA 12.2.2 (Luebke 2008), and R 4.2.1 (Team 2022). Simulations, data transformation, and MLE were handled through parallel CPU computations on the Hábrók high-performance computing cluster of the University of Groningen. The total computational budget for these processes was approximately 3000 h (used CPU time). Our neural networks were trained, optimized, and evaluated on the NVIDIA A100 and V100 Tensor Core GPUs of the Hábrók cluster. The estimated computational budget was 1500 h (used GPU time, excluding CPU time for data set loading and saving). We implemented a user-friendly illustrative tool to estimate parameters from phylogenetic trees using pretrained neural networks from this study in the new R package “EvoNN.”

### Simulation Approaches

To train the neural networks, we simulated phylogenetic trees using functions from different R packages. For each simulated data set, we kept trees with only extant lineages, mimicking reconstructed phylogenies. The settings for the parameters used to simulate the trees were selected to limit the maximum total number of nodes (including root, internal and tip nodes, here and after, we always refer to the total number of nodes) for the trees in each data set. After simulation, we further filtered out all trees containing more than 3000 nodes to avoid the creation of excessively large matrices that could deplete the available memory space allocated to the GPUs during the GNN training process. Such trees are uncommon under the settings we used—typically fewer than 5 trees with more than 3000 nodes ($\sim 1500$ tips) are present within each set of phylogenies we acquired from simulation. We also filtered out all trees containing less than 5 nodes (3 tips) to ensure successful data transformation and summary statistic computation. Small trees inherently carry limited informational content. The exclusion of these trees is unlikely to impact performance of the neural networks on the remaining trees (typically fewer than 100 out of 100,000 trees with less than 5 nodes were removed for each parameter setting).

To consider different diversification processes, we simulated 100,000 random BD trees (BD phylogenies), 100,000 diversity-dependent trees (DDD phylogenies), and 100,000 PBD trees (PBD phylogenies). The amount of simulated data is bounded by the resource and time limits of the computing cluster. All trees have an identical crown age of 10 time units ($t = 10$) to reduce both the complexity of the data and the computational burden. For simulating BD trees, we used the “rlineage” function from R package “ape” (Paradis and Schliep 2019) to generate complete trees and then pruned all the extinct lineages; for DDD trees, we used the “dd_sim” function from R package “DDD” (Etienne et al. 2012); for PBD trees, we used the “pbd_sim” function from our R package “eveGNN” (a codebase of phylogeny simulation, data transformation, neural network training, and MLE computation for our study), which is similar to the function with the same name in the original R package “PBD” (Etienne and Rosindell 2012), but only outputs necessary data for our study.

In our simulation approach, we randomly sampled the (log) parameters required for each scenario (BD, DDD, and PBD) from uniform distributions. The upper bound for the extinction rates was proportionally dependent on the drawn speciation rate to avoid cases where extinction rates could be larger than speciation rates, because in such cases the whole tree likely goes extinct. Furthermore, to prevent a huge number of events, which would deplete available computational time and memory, we also imposed an overall cap of 1.5 on the extinction rates.

Our choice of speciation and extinction rate ranges in the DDD scenario was informed by both computational considerations and biological context. For instance, speciation rates in birds have been estimated to range from 0.1 to 1 event per lineage per million years, whereas extinction rates often fall between 0 and 0.5 events per lineage per million years (Henao Diaz et al. 2019; Stadler and Bokma 2013).

See Table 1 for the detailed parameter distribution settings used in the simulations. Note that the Gillespie algorithm is scale-invariant, and therefore the absolute rate magnitudes are interchangeable with the length of the simulated time interval.

**Table 1.** Parameter settings for the simulated tree data sets.

A: parameter settings for BD and DDD trees												
			$\lambda _0$		$\mu _0$		*K*					
Type	Age	*N*	*a*	*b*	*a*	*b*	*a*	*b*				
BD	10	100,000	0.1	0.8	0.0	0.9$\lambda _0$	–	–				
DDD	10	100,000	0.1	4.0	0.0	${0.9\lambda _0}$ ^a^	10	1000				
**B:** parameter settings for PBD trees												
			$\lambda _1$		$\log _{10}(\lambda _2)$		$\lambda _3$		$\mu _1$		$\mu _2$	
Type	Age	*N*	*a*	*b*	*a*	*b*	*a*	*b*	*a*	*b*	*a*	*b*
PBD	10	100,000	0.1	1.0	−3	1	0.1	1.0	0.0	0.8$\lambda _1$	0.0	0.8$\lambda _3$

Parameter settings for the simulated tree data sets. The type column specifies which function is used to generate the trees. The columns specify the crown age (age), the number of trees in the data set (*N*), the lower (*a*) and the upper (*b*) bounds of the parameters for the tree simulations, all the parameters being sampled from *U*(*a, b*), except for $\lambda _1$ of the protracted BD scenario. $\lambda_1$ is computed as $\lambda _1 = 10^i$ where *i* is sampled from *U*(−3, 1). *U* denotes uniform distribution. Sub-table A shows the parameter distributions of the constant-rate BD model and the diversity-dependent-diversification model, $\lambda$: intrinsic speciation rate/birth rate; $\mu$: intrinsic extinction rate/death rate; *K*: clade-level carrying capacity. Sub-table B shows the parameter distributions of the protracted BD model, $\lambda _1$: speciation-initiation rate of good species; $\lambda _2$: speciation-completion rate; $\lambda _3$: speciation-initiation rate of incipient species; $\mu _1$: extinction rate of good species; $\mu _2$: extinction rate of incipient species.

a In diversity-dependent-diversification simulations, the maximum extinction rate is capped at 1.5 if $0.9\lambda > 1.5$ .

### Data Preparation

**Figure 1.**
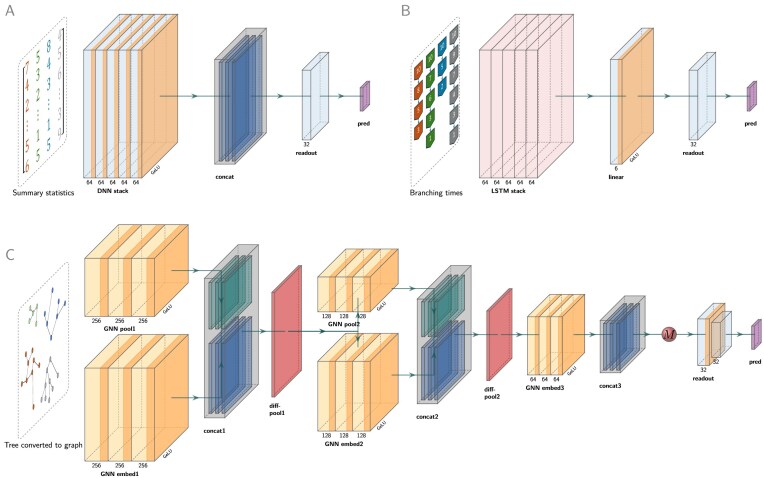
Illustration of the neural network architectures. From left to right, for each neural network, the inputs are filtered through the layers, and the network ultimately outputs the final predictions of the parameters through the readout layers. A: DNN, whose input data are summary statistics. The major component of the DNN is a stack comprising five linear layers (“DNN stack”), each followed by a Batch Normalization for 1D Inputs operator (BatchNorm1D, not shown in figure) and Gaussian error linear units (GELU, the orange band within the boxes). Learned features from all the linear layers within the stacks are collected and concatenated (“concat”). A single linear readout layer (“readout”) outputs *n* predicted parameters (“pred”). B: LSTM recurrent neural network, whose input data are the branching times. The major component of the LSTM is a stack of five LSTM recurrent neural network layers (“LSTM stack”). Learned features are processed by a linear layer accompanied by a GELU (“linear”), then passed to a single linear readout layer (“readout”) that outputs *n* predicted parameters (“pred”). C: GNN, whose input data is a graph representation of the phylogeny. GNN is assembled from five modules. Each module comprises the same number of GraphSAGE (sample-and-aggregate graph convolutional neural network) operators. Each operator is accompanied by a BatchNorm1d (not shown in the figure) operator and then a GELU activation function (illustrated by the orange bands within the yellow boxes). Learned features from all the GraphSAGE operators within a module are collected and concatenated. The differentiable pooling (DiffPool) technique is adopted to perform graph coarsening. In the first coarsening operation, the graph data inputs are passed to two GNN modules (“GNN pool1” and “GNN embed1”). The pooling group reduces the graph size, whereas the embedding group captures the node features. The filtered data from each GraphSAGE operator are concatenated (“concat1”) and then passed to a DiffPool layer (“diff-pool1”), which finalizes the first coarsening operation. The second coarsening operation is applied in the same way as the first (as represented by “GNN pool1,” “GNN embed2,” “concat2”), and the outputs from the second DiffPool layer (“diff-pool2”) are passed to the final (fifth) GNN module (“GNN embed3”). After the final GNN module, the outputs are concatenated (“concat3”) and transformed by a global mean pooling operation (red ball “M”) to create a final graph representation. This graph representation is passed to a readout layer group (“readout” as represented by light blue boxes) consisting of two linear layers to perform graph-level regression, which ultimately outputs a vector of *n* predicted parameters (“pred” as represented by a purple box). Only the first linear layer is followed by GELU (see the orange band of the first linear layer). See Supplementary Appendix C for the detailed description and technical details.

**Figure 2.**
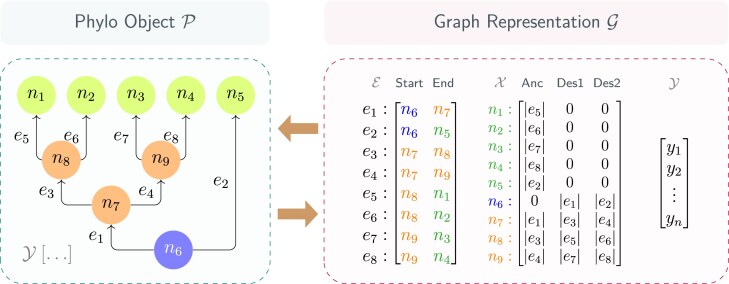
Illustration of data transformation between a “phylo” object and its graph representation. The left panel shows a visualization of a “phylo” object. The blue circle (n_6_) represents the root node, orange circles (n_7_ to n_9_) represent the internal nodes, and green circles (n_1_ to n_5_) represent the tip nodes. Arrows represent directed edges between each pair of the nodes. The right panel shows the transformed graph data structure. The adjacency list is denoted as $\mathcal {E}$. Each row of the adjacency list represents one edge, the first column represents the starting node, and the second column represents the end node. Note that the adjacency list is transposed (in the example into $\mathcal {E}^{2 \times 8}$) after converting to a tensor. The node feature matrix is denoted as $\mathcal {X}$. Each row of the node feature matrix represents the features contained in one node, the first column represents the distance from the node to its direct ancestor node, the second and the third columns represent the distances from the node to its two descendants. In the node feature matrix, the distances from a node to non-existing nodes (e.g., the tip nodes have no descendants, and the root node has no ancestor) are represented by zeros. The node and edge labels before the colons (including the colons) are placed here for visual assistance. After transformation, we use graph-level attributes $\mathcal {Y}$ to store the parameters used to generate the “phylo” object. The node labels are given by $n_1, n_2, n_3, \ldots , n_9$, the edge labels are given by $e_1, e_2, e_3, \ldots , e_8$, the edge lengths are given by $|{e_1}|, |{e_2}|, |{e_3}|, \ldots , |{e_8}|$. The generating parameters are given by a vector $[y_1, y_2, \ldots , y_n]$, where *n* is the number of parameters.

We employed three different basic neural network architectures: a dense neural network (DNN), a GNN, and a long short-term memory (LSTM) recurrent network, as illustrated in Figure 1 (see Supplementary Appendix C for a detailed description). Each of these architectures was refined through validation and required different input data. For the DNN, the input data consisted of a total of 54 summary statistics (Supplementary Appendix O) for each simulated tree. In the GNN, the full phylogeny was interpreted as a graph and could in that form be used as input data (as illustrated in Fig. 2). In the LSTM, we treated branching times of the phylogenies as sequential or time-series data (Sak et al. 2014). Given its recurrent architecture, LSTM is adept at sequence prediction tasks, making it particularly suitable for estimating tree parameters from entire sequences of branching times.

Therefore, our data comprises three major components: the phylogenetic trees, their corresponding summary statistics, and their branching times, to maximize the use of available data. The functions needed for the data transformations are either available in PyTorch or implemented in our package “eveGNN” and described in more detail in Supplementary Appendix A.

### Ensemble Learning Strategies

To leverage all available data and improve prediction accuracy, we combined GNN, DNN, and LSTM using bagging, stacking, and boosting, which are typical ensemble learning strategies (Graczyk et al. 2010). With bagging, we trained GNN, DNN, and LSTM independently on the same data set, translated their original outputs to parameter predictions (we will use “readout” hereafter to refer to this translation), and then aggregated the predictions. We used four aggregation methods: mean, median, max, and min.

With stacking, we used GNN, DNN, and LSTM in the same architecture but without their own readout layers. Instead, we combined the features learned from DNN, LSTM, and GNN and fed them to a meta-learner comprising linear neural network layers that learns the best readout parameter predictions from these combined features. GNN, DNN, LSTM, and the meta-learner were trained simultaneously.

With boosting, the neural networks were trained sequentially. Boosting strategies offer various pathways for enhancing model performance. We started with a GNN to make initial predictions and explored the effectiveness of both DNN and LSTM for correcting residuals, either individually or in sequence. We used “Boost SS” to refer to correcting GNN’s residuals by DNN (from summary statistics, thus “SS”); “Boost BT” to refer to correcting GNN’s residuals by LSTM (from branching times, thus “BT”); “Boost SS+BT” to refer to correcting GNN’s residuals by DNN and then correcting DNN’s residuals of residuals by LSTM; “Boost BT” to refer to correcting GNN’s residuals by LSTM (from branching times); “Boost BT+SS” to refer to correcting GNN’s residuals by LSTM and then correcting LSTM’s residuals by DNN.

**Figure 3.**
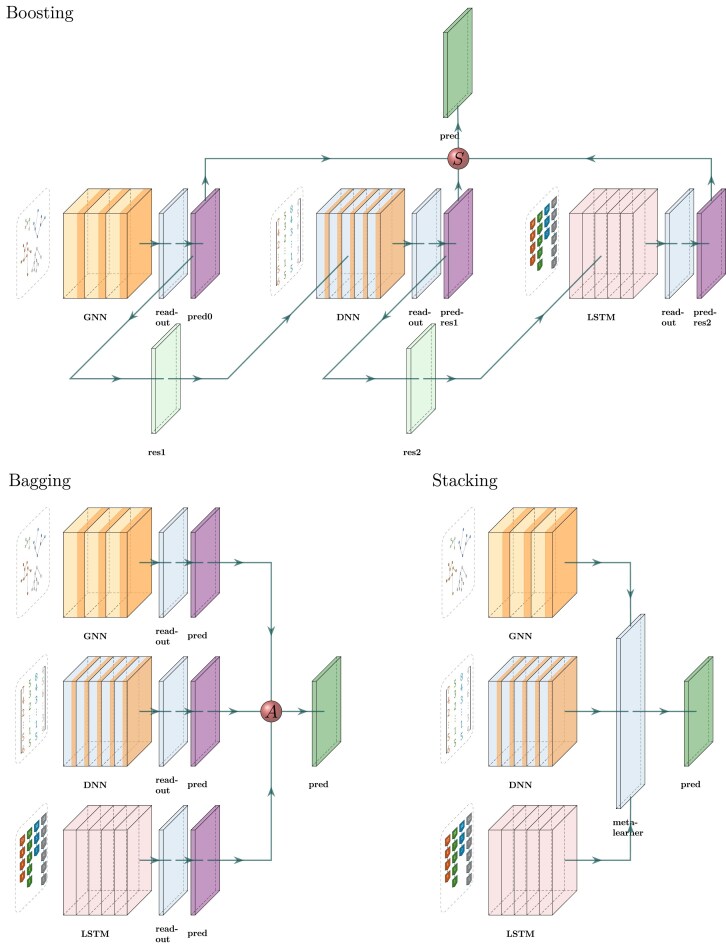
Illustration of the ensemble learning strategies to combine the GNN, the DNN, and the LSTM recurrent neural network. The neural networks are largely simplified. With boosting, GNN, DNN, and LSTM were trained sequentially to iteratively correct residuals. For example, DNN is trained to predict the residuals of GNN predictions. Subsequently, LSTM is trained to predict the residuals of the residuals after DNN corrected the GNN predictions. The final prediction comes from the initial prediction by GNN minus two learned residual terms by DNN and LSTM. With bagging, we trained GNN, DNN, and LSTM independently, translated their original outputs to parameter predictions, and then aggregated the predictions. With stacking, we trained GNN, DNN, and LSTM simultaneously but without readout. We directly concatenated the outputs from GNN, DNN, and LSTM and then used a meta-learner to make predictions from the outputs. With bagging, we trained GNN, DNN, and LSTM independently (“GNN,” “DNN,” and “LSTM” blocks of boxes), translated their outputs to parameter predictions through their own readout layers (three “readout” boxes next to the neural networks and three “pred” boxes next to the readout layers), and then aggregated the predictions (red ball “A”). With stacking, we trained GNN, DNN, and LSTM simultaneously (“GNN,” “DNN,” and “LSTM” blocks of boxes) but without their own readout layers. We combined the features from DNN, LSTM, and GNN and fed to a meta-learner (“meta-learner”) comprising linear neural network layers to output parameter predictions. With boosting, there can be different pathways. In our illustration, GNN, DNN, and LSTM were trained sequentially to iteratively correct residuals. First, the GNN is trained from the graphs to make the initial predictions (see “GNN,” “readout,” and then “pred0”), and from predicted and ground truth values of the parameters, we computed the residuals (“res1”); second, the DNN is trained to predict these residuals from the summary statistics (see “DNN,” “readout,” and then “pred-res1”), learning to correct the GNN’s errors; lastly, the LSTM is trained to predict the residuals of the residuals (see “LSTM,” “readout,” and then “pred-res2”), which is the initial predictions minus the predicted residuals by the DNN, from branching times, to further improve the predictive accuracy. Finally, we subtracted the two residual terms from the initial predictions (red ball “S”) to make the corrected predictions. See Supplementary Appendix D for a detailed explanation.

See Figure 3 for a simplified illustration of the ensemble learning strategies.

### Training Neural Networks

Prior to training, each data set of 100,000 trees was randomly shuffled and subsequently divided into two segments. The first segment, consisting of 90% of the data set, was allocated for training purposes, whereas the remaining 10% was used as the validation data set for monitoring and fine-tuning the neural network performance. The training session is carried out by epochs, each consisting of three major steps: first, performing forward pass on the training data set; second, assessing the prediction accuracy; and lastly, performing back-propagation (adjusting the weights of the neuron connections to improve the neural network performance). Back-propagation, requires quantifying the error between the neural networks’ predictions and the actual ground truth values. We quantified the “total loss” as the sum of the residual error and other terms for facilitating neural network training. We represented total loss using a loss function that sums up all the loss terms (see Supplementary Appendix B for more detail).

We use the AdamW (Adaptive Moment Estimation with decoupled weight decay) optimizer (Loshchilov and Hutter 2017) to iteratively update the neural networks’ parameters to minimize the loss function. We used default AdamW argument settings. During training, we adopted mini-batches of size 64 (data of 64 simulated trees per mini-batch) to reduce GPU memory usage. The total number of epochs was manually optimized per architecture to avoid underfitting and overfitting. This was done by comparing the loss metrics for the training data set to those of the validation data sets at every epoch. Overfitting is indicated by a training loss that continues to decrease while the validation loss starts to increase, whereas underfitting is suggested by both training and validation losses being high and decreasing at a similar rate. Analyzing these loss trends over time can help to optimize hyper-parameters (“settings” that might alter neural network behavior or impact performance). Early in the study, we used early stopping to identify the optimal training duration. Once we observed that our simulated data sets converged at highly consistent time points (because the simulated data sets are homogeneous between simulations and splits), we switched to manually setting training lengths—guided by diagnostic metrics—to achieve the best balance between training and testing performance.

Under the BD scenario, the neural networks were trained to predict two parameters: birth rate ($\lambda$) and death rate ($\mu$). Under the DDD scenario, the neural networks were trained to predict three parameters: speciation rate ($\lambda$), extinction rate ($\mu$), and carrying capacity (*K*). Under the PBD scenarios, the neural networks were trained to predict five parameters: speciation rate of the good species ($\lambda _1$), speciation completion rate ($\lambda _2$), speciation rate of the incipient species ($\lambda _3$), extinction rate of the good species ($\mu _1$), and extinction rate of the incipient species ($\mu _2$).

To enable a direct comparison between our networks and an existing method, we additionally implemented a one-dimensional convolutional neural network architecture (CNN1D here and after) described by Lajaaiti et al. (2023); Voznica et al. (2022) to estimate parameters from branching times.

### MLE as Baseline Benchmark

MLE approaches have been developed for the BD, DDD, and PBD scenarios (Etienne et al. 2012; Etienne and Rosindell 2012; Etienne et al. 2014). Per scenario, we simulated additional testing data sets each comprising 10,000 phylogenies using the same parameter spaces as the training data sets. For these testing data sets, we adopted the MLE approaches to estimate the parameters of each phylogeny from their branching times under different scenarios. For the BD trees, we estimated their birth and death rates. For the DDD trees, we estimated their speciation rate, extinction rate, and clade-level carrying capacity. There is a limitation in the MLE approach for PBD trees because to allow for a computation of the likelihood, the speciation initiation rates of good species and incipient species need to be equal (Etienne et al. 2014). We therefore only estimated four initial parameters: speciation initiation rate (for both good and incipient species, assuming they are the same), speciation-completion rate, extinction rate of good species, and extinction rate of incipient species, although in our simulation we have five independently sampled parameters. The MLE prediction accuracies are used as a baseline benchmark to evaluate the prediction accuracies of the neural networks on tree parameter estimation.

We implemented two different MLEs: one with ground truth parameter values set as the starting point of the MLE searching process, the other with a starting point randomly sampled from the parameter space of the simulation. We consider the first type of benchmarks as a best-case MLE performance (as in real applications ground truth parameters are not known) and the other type as a naive-case MLE performance, which mimics the pragmatic approach if true parameter values are not known. Note that in practice it is possible to achieve much better performance than the naive-case, for example, by optimizing from several random starting points to avoid being stuck in local optima.

We also explored the effectiveness of different optimization and integration approaches for MLE on the DDD phylogenies. We used the “Simplex” (Lagarias et al. 1998) optimizer. See Supplementary Appendix E for reasons and for a detailed comparison between the optimizers.

### Performance Analysis

We used the same testing data set as was used for MLE parameter estimation to obtain neural network parameter predictions. To evaluate the performance of each method (MLE benchmarks and different neural network architectures), we analysed the patterns of residuals (differences between ground truth and predicted values, which can be viewed as the goodness of fit) by examining their relation to true values and the total node counts of the phylogenetic trees, which include root, internal, and tip nodes. Considering the complex nature of residual patterns, which may vary according to specific characteristics of the simulation processes (for instance, carrying capacity effects in DDD and protracted speciation in PBD), as well as the performance and robustness of the estimation methods, we calculated error metrics locally for three different phylogeny size ranges, as a global metric could be misleading.

Our observation is mainly based on simulated trees under the DDD scenario, because it involves more evolutionary mechanisms than the simple BD scenario while containing fewer parameters than the protracted BD scenario. This simplifies our analyses on the neural network performance while maintaining enough complexity to challenge the capability of our proposed methods. From this case study, we identified and selected the most effective MLE optimization algorithm, neural network architecture, and ensemble strategy, which we then applied to BD and PBD scenarios. We therefore only analysed the best-performing neural network methods against the naive and best MLE cases on BD and PBD. Additionally, for the BD scenario, we computed net diversification rate ($\lambda -\mu$) and extinction-to-speciation ratio ($\mu /\lambda$); for the PBD scenario, we computed a composite parameter called the mean duration of speciation from the speciation completion rate, the speciation rate of incipient species, and the extinction rate of incipient species, because MLE can arguably better estimate the mean duration of speciation than the original parameters (Etienne et al. 2014).

### Robustness Analysis

We assessed the robustness of the estimation results of both neural networks and MLE by measuring the consistency with which these approaches produce similar estimates for phylogenies generated under identical parameter settings. We also applied this bootstrapping method to assess the estimation robustness of empirical trees; the details are provided in the following section. In the previous simulations, each parameter combination was sampled and used only once, whereas in the robustness analysis we repeatedly use identical parameter settings to generate sets of phylogenies (bootstrapping) under the DDD scenario. Even when the same parameters are used, the resulting phylogenies can vary substantially in size, topology, and structure due to stochasticity. Such an evaluation helps assess the neural networks’ ability to abstract the underlying parameter influences from the phylogenetic data, regardless of heterogeneity. For each parameter combination, 1000 trees were generated randomly. We used a total of 80 sets of parameter combinations, thus 80,000 phylogenies in total. Specifically, we used all combinations of speciation rates $\lambda = 1.0, 1.5, 2.0, 2.5, 3.0$, extinction rates $\mu = 0.2, 0.4, 0.6, 0.8$, and carrying capacities $K = 200, 400, 600, 800$.

**Figure 4.**
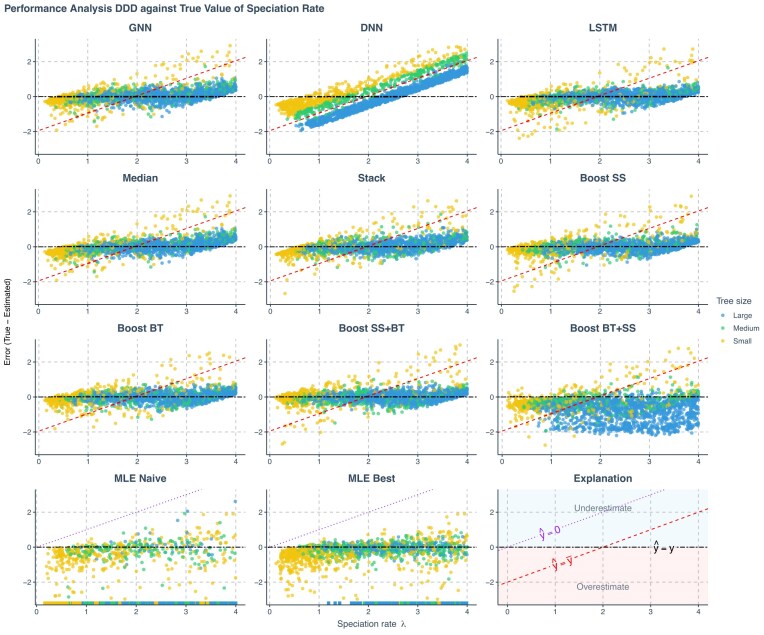
Prediction error of various methods applied to phylogenies simulated under a DDD scenario, against true values of the speciation rate. The errors shown (*y*-axis) are the differences between the true parameters (*x*-axis) used to simulate the phylogenies and the values predicted or estimated by each method. Each panel represents an estimation method. Phylogenies are categorized based on their size: yellow for small phylogenies with fewer than 200 nodes (including root, internal, and tip nodes), green for medium-sized phylogenies with 200–500 nodes, and blue for large phylogenies with more than 500 nodes, refer to Supplementary Appendix G, Figure 19 for how the tree sizes are distributed. GNN: Predictions obtained by the GNN using the phylogenies. DNN: Predictions by the DNN using summary statistics. LSTM: Predictions by the LSTM recurrent neural network using branching times. Median: Bagging strategy that takes the median value of the predictions from GNN, DNN, and LSTM. Stack: Stacking strategy that utilizes a meta-learner to integrate results from GNN, DNN, and LSTM. Boost SS: Boosting strategy that DNN corrects GNN results using summary statistics (SS). Boost BT: Boosting strategy that LSTM corrects GNN results using branching times (BT). Boost SS+BT: Sequential correction of GNN errors first using DNN, followed by LSTM. Boost BT+SS: Sequential correction of GNN errors first using LSTM, followed by DNN. MLE Naive: maximum likelihood estimation results using a random starting point within the parameter space of the training data set for each parameter’s optimization. MLE Best: MLE results using the true parameter values as the starting points for optimization. Red dashed lines in panels representing neural network results indicate the mid-points of the parameter spaces ($\hat{y}=\bar{y}$ where $\hat{y}$ denotes a estimated parameter and $\bar{y}$ denotes the mid-point of the parameter space). Data points close to purple dotted lines ($\hat{y}=0$) in MLE result panels indicate near-zero estimates. Black two-dash lines indicate accurate estimates ($\hat{y}=y$ where *y* denotes the true parameter value). In the MLE result panels, small squares spreading along the *x*-axis signify optimization failures. Due to significantly lower accuracy, other aggregation methods from the bagging strategy are not displayed on the plot. $\lambda$: Speciation rate.

**Figure 5.**
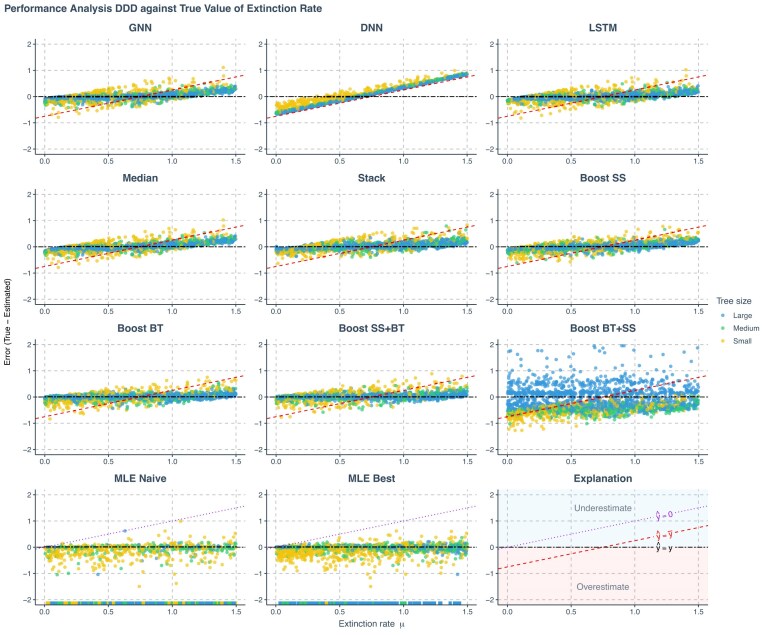
Prediction error of various methods applied to phylogenies simulated under a DDD scenario, against true values of the extinction rate. The errors shown (*y*-axis) are the differences between the true parameters (*x*-axis) used to simulate the phylogenies and the values predicted or estimated by each method. Each panel represents an estimation method. Phylogenies are categorized based on their size: yellow for small phylogenies with fewer than 200 nodes (including root, internal, and tip nodes), green for medium-sized phylogenies with 200–500 nodes, and blue for large phylogenies with more than 500 nodes. GNN: Predictions obtained by the GNN using the phylogenies. DNN: Predictions by the DNN using summary statistics. LSTM: Predictions by the long short-term memory recurrent neural network using branching times. Median: Bagging strategy that takes the median value of the predictions from GNN, DNN, and LSTM. Stack: Stacking strategy that utilizes a meta-learner to integrate results from GNN, DNN, and LSTM. Boost SS: Boosting strategy that DNN corrects GNN results using summary statistics (SS). Boost BT: Boosting strategy that LSTM corrects GNN results using branching times (BT). Boost SS+BT: Sequential correction of GNN errors first using DNN, followed by LSTM. Boost BT+SS: Sequential correction of GNN errors first using LSTM, followed by DNN. MLE Naive: maximum likelihood estimation results using a random starting point within the parameter space of the training data set for each parameter’s optimization. MLE Best: MLE results using the true parameter values as the starting points for optimization. Red dashed lines in panels representing neural network results indicate the mid-points of the parameter spaces ($\hat{y}=\bar{y}$ where $\hat{y}$ denotes a estimated parameter and $\bar{y}$ denotes the mid-point of the parameter space). Data points close to purple dotted lines ($\hat{y}=0$) in MLE result panels indicate near-zero estimates. Black two-dash lines indicate accurate estimates ($\hat{y}=y$ where *y* denotes the true parameter value). In the MLE result panels, small squares spreading along the *x*-axis signify optimization failures. Due to significantly lower accuracy, other aggregation methods from the bagging strategy are not displayed on the plot. $\mu$: extinction rate.

**Figure 6.**
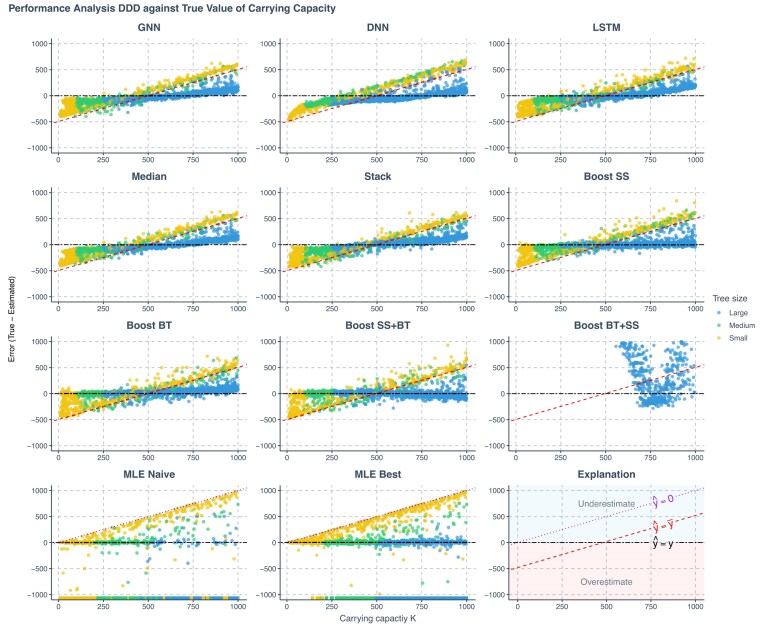
Prediction error of various methods applied to phylogenies simulated under a DDD scenario, against true values of the carrying capacity. The errors shown (*y*-axis) are the differences between the true parameters (*x*-axis) used to simulate the phylogenies and the values predicted or estimated by each method. Each panel represents an estimation method. Phylogenies are categorized based on their size: yellow for small phylogenies with fewer than 200 nodes (including root, internal, and tip nodes), green for medium-sized phylogenies with 200–500 nodes, and blue for large phylogenies with more than 500 nodes. GNN: Predictions obtained by the GNN using the phylogenies. DNN: Predictions by the DNN using summary statistics. LSTM: Predictions by the long short-term memory recurrent neural network using branching times. Median: Bagging strategy that takes the median value of the predictions from GNN, DNN, and LSTM. Stack: Stacking strategy that utilizes a meta-learner to integrate results from GNN, DNN, and LSTM. Boost SS: Boosting strategy that DNN corrects GNN results using summary statistics (SS). Boost BT: Boosting strategy that LSTM corrects GNN results using branching times (BT). Boost SS+BT: Sequential correction of GNN errors first using DNN, followed by LSTM. Boost BT+SS: Sequential correction of GNN errors first using LSTM, followed by DNN. MLE Naive: Maximum Likelihood Estimation results using a random starting point within the parameter space of the training data set for each parameter’s optimization. MLE Best: MLE results using the true parameter values as the starting points for optimization. Red dashed lines in panels representing neural network results indicate the mid-points of the parameter spaces ($\hat{y}=\bar{y}$ where $\hat{y}$ denotes a estimated parameter and $\bar{y}$ denotes the mid-point of the parameter space). Data points close to purple dotted lines ($\hat{y}=0$) in MLE result panels indicate near-zero estimates. Black two-dash lines indicate accurate estimates ($\hat{y}=y$ where *y* denotes the true parameter value). In the MLE result panels, small squares spreading along the *x*-axis signify optimization failures. Due to significantly lower accuracy, other aggregation methods from the bagging strategy are not displayed on the plot. *K*: carrying capacity.

**Figure 7.**
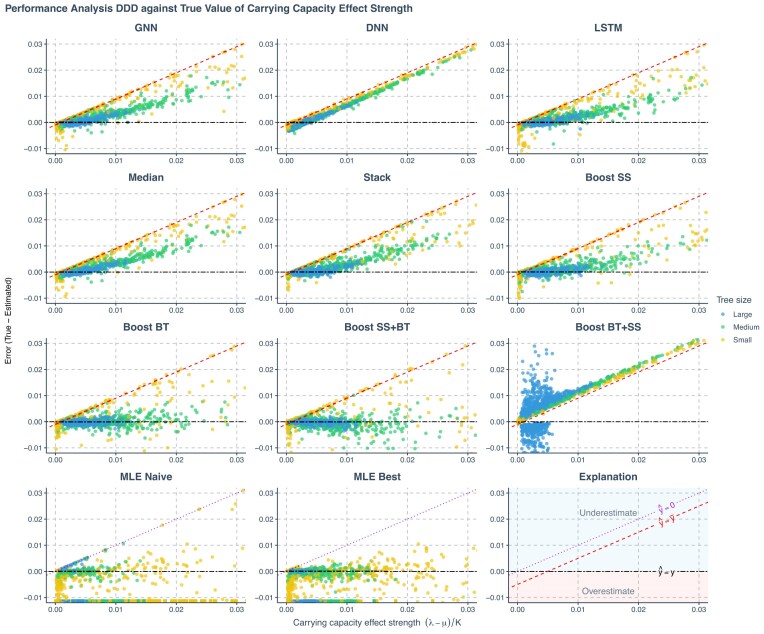
Prediction error of estimated carrying capacity effect computed from estimated values of speciation rate, extinction rate, and carrying capacity using various methods applied to phylogenies simulated under a DDD scenario, plotted against the true carrying capacity effect computed from true parameters. The errors shown (*y*-axis) are the differences between the values of true carrying capacity effect (*x*-axis) used to simulate the phylogenies and the values predicted or estimated by each method. Each panel represents an estimation method. Phylogenies are categorized based on their size: yellow for small phylogenies with fewer than 200 nodes (including root, internal, and tip nodes), green for medium-sized phylogenies with 200–500 nodes, and blue for large phylogenies with more than 500 nodes. GNN: Predictions obtained by the GNN using the phylogenies. DNN: Predictions by the DNN using summary statistics. LSTM: Predictions by the long short-term memory recurrent neural network using branching times. Stack: Stacking strategy that utilizes a meta-learner to integrate results from GNN, DNN, and LSTM. Boost SS: Boosting strategy that DNN corrects GNN results using summary statistics (SS). Boost BT: Boosting strategy that LSTM corrects GNN results using branching times (BT). Boost SS+BT: Sequential correction of GNN errors first using DNN, followed by LSTM. MLE Naive: maximum likelihood estimation results using random starting points for parameter optimization. MLE Best: MLE results using the true parameter values as the starting points for optimization. Red dashed lines in panels representing neural network results indicate the mid-points of the parameter spaces ($\hat{y}=\bar{y}$ where $\hat{y}$ denotes an estimated parameter and $\bar{y}$ denotes the mid-point of the parameter space). Data points close to purple dotted lines ($\hat{y}=0$) in MLE result panels indicate near-zero estimates. Black two-dash lines indicate accurate estimates ($\hat{y}=y$ where *y* denotes the true parameter value). In the MLE result panels, small squares spreading along the *x*-axis signify optimization failures. $\lambda$: Speciation rate. $\mu$: extinction rate. *K*: carrying capacity.

MLE is computationally more expensive than predicting from already trained neural networks, and computational time rapidly increases with the size of the phylogenies. We thus performed MLE on only 2000 simulated phylogenies. To ensure fair visual and numerical comparisons when plotting the results of these analyses, extreme MLE estimates were not shown in the figures (they exceeded the fixed range of the *y*-axis) and excluded from the computation of the mean absolute errors of the MLE estimates. Neural network results were randomly sub-sampled to match the MLE data count, maintaining equivalent visual density and facilitating a more accurate performance comparison between approaches. For the neural networks, the mean absolute errors were computed on the complete data set without sub-sampling and exclusion. In Figures 4–7, on average (we simulated the testing data sets many times throughout the study), out of 2000 samples, 5–150 samples per MLE figure panel, and 1–3 per neural network figure panel fell beyond the axis range. For the most underperforming method (Boost BT+SS) 600–1500 samples fell beyond the axis range.

We did not analyze the robustness of BD and PBD scenarios, because BD is a special case of DDD (if we set carrying capacity to an infinite value) and PBD related parameters can hardly be estimated accurately using MLE methods (Etienne et al. 2014).

The complete code base for this study, including simulations, data processing, neural network training, evaluation, and both data analysis and visualization tools, is available in the GitHub repository “eveGNN” (Qin 2025).

### Misspecification Analysis

To further assess the performances of MLE and neural network approaches when confronted with misspecified assumptions, we treated simulated BD trees as if they were produced under a DDD model. Specifically, we applied the MLE algorithm designed for the DDD scenario and used two neural networks—GNN and Boost BT—trained on DDD trees to estimate the speciation rate $\lambda$, extinction rate $\mu$, and clade-level carrying capacity *K*. Under a true BD process viewed through the DDD perspective, $K\rightarrow \infty$.

To compare naive and best-case performances of MLE under estimation method misspecification, we set the starting points of the optimizer using two strategies: one in which the initial *K* was set to 10,000 (naive-case) and another in which *K* was set to $\infty$ (best-case). In both cases, the starting points of $\lambda$ and $\mu$ were set to the true parameters used to simulate the BD trees. For each case, we summarized the proportions of MLE estimates falling into four intervals relative to the total number of nodes $N_{\mathrm{nodes}}$: (1) between $2\, N_{\mathrm{nodes}}$ and $5\, N_{\mathrm{nodes}}$, (2) between $5\, N_{\mathrm{nodes}}$ and $20\, N_{\mathrm{nodes}}$, (3) between $20\, N_{\mathrm{nodes}}$ and $\infty$, and (4) exactly $\infty$ (by exactly we mean the MLE algorithm gives infinite *K* estimate).

We computed the expected $N_{\mathrm{nodes}}$ under the BD process as a function of the net diversification rate and simulation time *t*,


\begin{eqnarray*}
\mathbb {E}[N_{\mathrm{nodes}}(t)] \,\,=\,\, 2\, \mathbb {E}[N_{\mathrm{tips}}(t)] - 1 \,\,=\,\, 2\, e^{(\lambda -\mu )t} \,\,-\,\, 1
,
\end{eqnarray*}

where $t=10$ for all trees. This expectation guided the interpretation of estimated *K* magnitudes relative to the total number of nodes of the trees being estimated.

### Empirical Tree Estimation

We deployed pre-trained neural networks to estimate phylogenetic parameters from a data set of 199 empirical phylogenetic trees curated by Condamine et al. (2019), with a tip count ranging from 20 to 1500. To align with the training conditions of our neural networks, which were trained on simulated phylogenies spanning exactly 10 time units (Myr), we rescaled the crown ages of all empirical trees to this duration. The parameter estimates we present are therefore rescaled. All the selected empirical trees are reconstructed phylogenies and fully bifurcated (each root or internal node has exactly two descendants). If an empirical tree fails an ultrametric (all tip-ends are aligned at the present) test due to branch length precision issue, we forced all its tips to end exactly at the present by extending the shorter tips to align with the longest one. See Supplementary Appendix N for meta information of the empirical trees.

We used two distinct neural networks, each pre-trained on simulated trees from one of two evolutionary scenarios (BD or DDD) to estimate parameters from the empirical trees. For the BD scenario, we estimated the parameters $\lambda$ (speciation rate) and $\mu$ (extinction rate); for the DDD scenario, we estimated $\lambda$, $\mu$, and *K* (carrying capacity). We did not estimate parameters for the PBD scenarios because neither neural networks nor MLE approaches could recover the individual parameters accurately from the simulated phylogenies. In addition to our neural network estimates, we used MLE methods for parameter estimation to provide a comparative assessment of the results. The MLE methods were set to use default starting points of likelihood optimization, as we do not know the true parameters of the empirical phylogenies.

We used the same bootstrapping method described before to quantify the uncertainties of both MLE and neural network estimates from empirical data. The process involves three main steps: first, estimating parameters from empirical phylogenies using MLE and pre-trained neural networks; second, simulating a set of phylogenies under a specified diversification scenario (such as BD, DDD, or PBD) using the MLE and neural network estimates; and third, re-estimating parameters from the simulated phylogenies using MLE and neural networks. The estimates derived from the bootstrapped phylogenies form a distribution.

We applied this uncertainty computation to a selected set of empirical phylogenies from the Condamine data set (Condamine et al. 2019) under the DDD scenario (see Supplementary Appendix F for details). The criteria for selection were phylogenies with more than 300 and less than 1000 nodes, and MLE of *K* (carrying capacity) being less than 1000. The distributions of MLE and neural network estimates from the bootstrapped phylogenies were compared with the original MLE and neural network estimates from empirical phylogenies. For each set of parameters estimated from empirical phylogenies, we bootstrapped 1000 simulated phylogenies.

Our R package “EvoNN” (Qin 2024) provides functions to perform the uncertainty (bootstrap) analyses.

### Supplementary Studies

To account for the potential effects of under- and over-representation of phylogenies of different sizes in our data sets, we conducted a supplementary study to explore whether the patterns we observed persist in a data set with a re-balanced distribution of phylogeny sizes (see Supplementary Appendix K, Fig. 33) for details.

To explore the generalization ability of the neural networks when facing data with true parameters completely outside the training space, as well as to compare neural network performances between extant-only phylogenies and complete phylogenies with extinct species, we also conducted another supplementary study. MLE and neural network approaches were examined on in-distribution and out-of-distribution data sets crossed with extant and complete trees. See Supplementary Appendix L for details.

## Results

### Performance Analysis

We evaluated the performances of various neural networks, both individually and in combination through ensemble strategies, in predicting parameters from simulated DDD phylogenies. These predictions were compared against best-case and naive-case MLE results using the Simplex optimizer.

**Figure 8.**
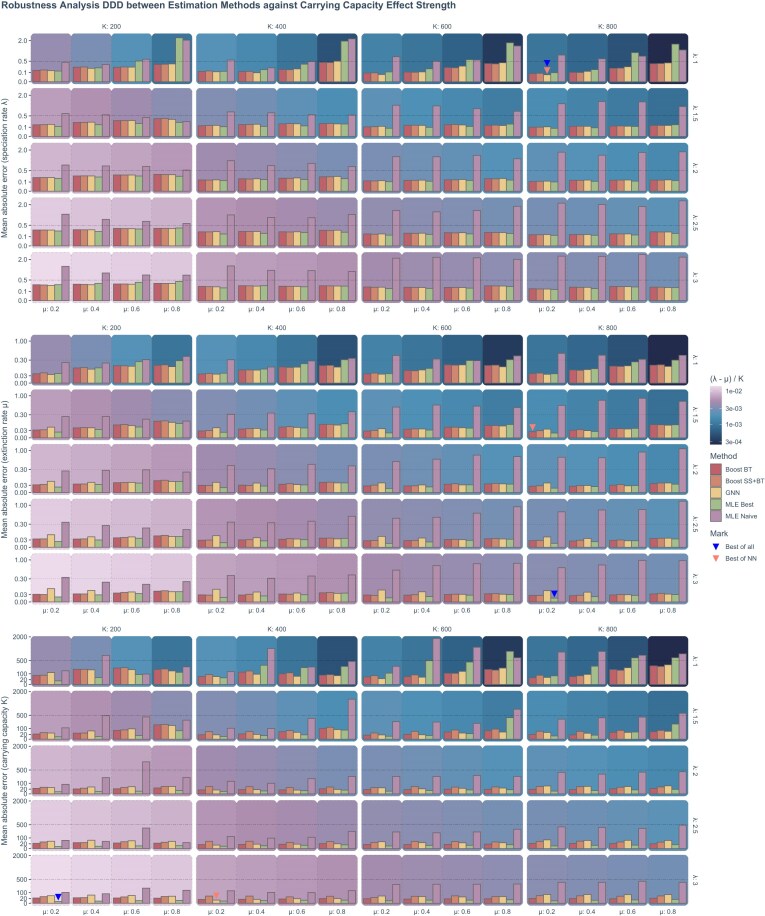
The robustness (mean absolute error) of neural network and MLE was assessed for 80 sets of phylogenies, each containing 1000 trees randomly simulated under a DDD scenario, employing identical parameter settings but varying in size, topology, and structure. The robustness results of the speciation rate, the extinction rate, and the carrying capacity are shown from top to bottom. For each panel group associated to a parameter, each panel contains the robustness of different estimation methods (the MLE and neural networks) under a combination of parameters indicated by the facet strip labels. Each facet column represents the robustness under a specific carrying capacity (*K*) setting used in the simulation of the phylogenies. Each facet row represents a specific speciation rate ($\lambda$). Each group of the bars represents a specific extinction rate ($\mu$) as shown by the *x*-axis. The background color of a panel represents the carrying capacity effect strength (calculated as $(\lambda -\mu )/K$ and visualized in “log10” scale), from bottom-left to top-right, the carrying capacity effect strength decreases. The color of a bar represents the associated estimation method. Boost BT: Boosting strategy that LSTM corrects GNN results using branching times (BT). Boosting SS + BT: GNN with DNN and LSTM recurrent neural network correcting residuals sequentially using summary statistics (SS) and branching times (BT). GNN: graph neural network. MLE Best: maximum likelihood estimation using true parameters as the starting points. MLE Naive: maximum likelihood estimation using a random value as the starting point of optimization for each parameter. *X*-axis: Represents extinction rate ($\mu$) settings. *Y*-axis: Represents the mean absolute error in a square-root transformed scale. Some bars are marked; for each parameter, the blue triangle represents the greatest possible robustness achieved among all the estimation methods, the red triangle represents the greatest possible robustness achieved among the neural network methods.

Among all the methods, we consider boosting GNN with LSTM as the most robust method based on the goodness of fit (see Figs. 4–7), the mean absolute errors (see Supplementary Appendix G, Fig. 16), and robustness (see rows named Boost BT in Fig. 8 and Fig. 17). Both neural networks and MLE approaches generally struggled with small phylogenies (see Figs. 4–6 with larger errors for the small phylogenies represented by the yellow points, see also Supplementary Appendix G, Fig. 16). Performance improved significantly on medium and large phylogenies for both neural network and MLE approaches.

The MLE implementation sometimes fails to find an optimal solution, partly due to numerical overflow issues. In our visualizations, failed MLE estimations are indicated by small squares spreading along the *x*-axis to avoid misinterpretation. MLE tended to give small or near-zero estimates, particularly for the carrying capacity. This phenomenon is more prominent when starting optimization from a random point. For all figures showing the MLE error, the ideal situation is that all the data points lie near the horizontal black two-dash reference lines (at which the error is 0) and do not spread along or near the purple dotted reference lines (which suggests near-zero MLE estimations). See the last panel at the bottom right for explanation; then also refer to the left two panels in the last row (the MLE results) in Figures 4–6 with the help of the explanation panel. Some MLE estimates are removed from the visualizations due to disagreements of results between different MLE settings, see Supplementary Appendix E for details. Therefore, the MLE performances shown are both visually and statistically better than that in the complete results, particularly for the best case, as the disagreements occur mainly when MLE struggles to recover parameters accurately.

Neural networks often return values closer to the parameter space’s mid-points, a result of making “safer” predictions that minimize loss compared with random guesses. Note that the *y*-axis in Figures 4–7 represents estimation error, which is why the red dashed lines indicating the mid-points cross $y = 0$ at $x = (\mathrm{ lower bound} + \mathrm{ upper bound})/2$. For example, for speciation rate $\lambda$ in Figure 4, we observe $y = 0$ when $x = (0.1 + 4.0) / 2 = 2.05$ ). Consequently, neural networks usually overestimate at low true values and underestimate at high true values (see Figs. 4–6). These errors are mitigated or partially corrected when the neural networks are trained in tandem through boosting strategies, for example, boosting GNN results with DNN or LSTM or both (see the panels of Boost SS, Boost BT, and Boost SS+BT in Figs. 4–6). This happens particularly for large phylogenies (the blue data points in Supplementary Appendix G, Fig. 16) when the underlying true carrying capacity (*K*) is large, or for small phylogenies (the yellow data points) when the underlying true speciation rate ($\lambda$) is small.

However, boosting strategies can introduce their own challenges. When boosting GNN results first with LSTM and then with DNN, the DNN failed to identify a general pattern of errors from LSTM results. This led to overfitting on the training data set at the second epoch of the training session (the total loss in the validation data set started to increase and became much larger than the total loss in the training data set), which, in turn, resulted in poor performance on the testing data set (see the panels named Boost BT+SS in Figs. 4–6 and Supplementary Appendix G, Fig. 16).

Upon further analysis of the residuals, we observed that inaccuracies in the predictions were largely influenced by the size of the phylogeny (Figs. 4–6). For neural network approaches, the prediction errors for speciation rate, extinction rate, and carrying capacity tended to increase as the size of the phylogeny decreased, especially in phylogenies with fewer than 200 nodes. Systematic error was also identified in the estimate of carrying capacity: neural networks generally overestimated this parameter in smaller phylogenies and underestimated it in larger ones. Boosting strategies were effective in mitigating or partially correcting systematic errors, and enhancing prediction accuracy, particularly for carrying capacity (see the rows of Boost SS, Boost BT, and Boost SS+BT in Supplementary Appendix G, Fig. 16).

We calculated the strength of the carrying capacity effect using the formula $1/K^{\prime } = (\lambda -\mu ) / K$, where $\lambda$ represents the true speciation rate, $\mu$ the true extinction rate, *K* the true carrying capacity, and $K^{\prime }$ the diversity at which speciation becomes zero for linear negative diversity-dependence (Etienne et al. 2012). A larger $(\lambda -\mu ) / K$ value corresponds to smaller $K^{\prime }$ and therefore a stronger carrying capacity effect. In the case of smaller phylogenies, neural networks tended to underestimate speciation and extinction rates while overestimating carrying capacity when the carrying capacity effect is weak, and the reverse is observed when the effect is strong. In contrast, MLE tends to overestimate speciation and extinction rates while underestimating carrying capacity under conditions of weak carrying capacity effect, with the reverse occurring under strong effects, except for the carrying capacity, which is always underestimated (see Supplementary Appendix G, Fig. 16). Neural network methods tend to underestimate the carrying capacity effect. This phenomenon can be mitigated by the boosting strategies, especially the Boost BT method, which achieved a performance similar to that of the best-case MLE estimates (see Fig. 7).

Unlike GNN and LSTM, DNN cannot by itself reliably recover speciation and extinction rates from the summary statistics of the phylogenies, with its predictions mostly clustering around the mid-points of the parameter space (around the red dashed lines in the DNN panels in Figs. 4–6). The overall accuracy of the carrying capacities recovered by the DNN is also lower than the accuracy recovered by the other approaches (see the row named DNN in Supplementary Appendix G, Fig. 16).

Among all ensemble learning strategies, boosting consistently outperformed both bagging and stacking in enhancing prediction accuracy compared with using neural networks independently, as can be seen, for instance, by the lower mean absolute prediction errors in Supplementary Appendix J, Figure 16. Boosting strategies also exhibited better performance in recovering the true values of the carrying capacity effect (see Fig. 7). The most effective neural network approaches overall matched or even surpassed the results of MLE while exhibiting no bias, even on smaller phylogenies. Overall, sequential boosting of GNN results first with DNN and then with LSTM (Boost SS+BT) led to best performance in terms of prediction accuracy, except for estimating carrying capacity on large phylogenies with around 2000 nodes (see the row named Boost SS+BT in Supplementary Appendix G, Fig. 16 this strategy led to overestimation on very large phylogenies). However, Boost SS+BT led to more overestimation on the true values of the carrying capacity effect, as compared with the strategy of boosting the GNN results with only LSTM (Boost BT, see Fig. 7).

In general, boosting methods (except for Boost BT + SS) yielded more accurate and less biased estimates across tree sizes, matching or outperforming MLE. In particular, the Boost SS+BT strategy achieved the highest overall accuracy by improving inference of carrying capacity effects.

The neural network architecture by Voznica et al. (2022) performed similarly and exhibited similar patterns as our approaches. CNN1D better recovered the carrying capacity effect strength than GNN, DNN, and LSTM alone, but lagged behind our boosting approaches—except for Boost BT+SS—in overall parameter prediction accuracy (see Figs. 28–32 in Supplementary Appendix J).

### Robustness Analysis

As a proxy for robustness of each method, we used the mean absolute errors of the parameters estimated from sets of phylogenies simulated under identical true parameters. Our analysis indicates that the robustness of the methods against phylogenetic heterogeneity (e.g., phylogenies of very different sizes, topologies, and other characteristics) depends on the values of the underlying true parameters. We observed that the strength of the carrying capacity effect critically influences robustness. Generally, a weaker carrying capacity effect (associated to a smaller value of $(\lambda -\mu ) / K$) tends to diminish the robustness of both MLE and neural network methods across all parameters: speciation rate, extinction rate, and carrying capacity (as can be seen in Fig. 8 and Supplementary Appendix G, Figs. 17 and 18, by observing the increase of error along with the darkening background colors from light pink to dark blue).

When the carrying capacity effect is weak, neural network methods typically exhibit greater robustness in estimating speciation and extinction rates compared with the best-case MLE results (see Fig. 8 and Supplementary Appendix G, Fig. 18). When the carrying capacity effect is exceptionally strong, the best-case MLE results can outperform neural networks particularly when estimating carrying capacity. Naive-case MLE results consistently show less robustness compared with all neural network methods.

A higher extinction rate generally decreases the robustness of all methods in estimating any parameter. A higher speciation rate enhances the robustness of carrying capacity estimates across all methods, although its impact on the robustness of speciation and extinction rate estimates is not consistent. A higher carrying capacity generally decreases the robustness of all methods in estimating carrying capacity.

Note that MLE naive-case results often contained more extreme estimates than best-case results, consequently, the exclusion of extreme values could wrongfully give the impression in the figures that when the carrying capacity effect is weak, the naive-case MLE is more robust than the best-case MLE. This is particularly prominent for the speciation rate. The exclusion of these extreme values is crucial, however, as they are rare, and their magnitude can obstruct meaningful interpretation and comparison.

We find that DNN alone (estimating parameters from summary statistics) shows the worst robustness among all the methods and LSTM alone (estimating parameters from branching times) shows the greatest robustness overall. Among all the estimation methods, the MLE best-case achieved the greatest possible robustness in estimating the extinction rate and the carrying capacity, whereas GNN alone (estimating parameters from phylogenies) achieved the greatest possible robustness in estimating the speciation rate. Among the neural network methods, GNN alone achieved the greatest possible robustness in estimating the speciation rate and the carrying capacity, whereas Boost BT (boosting GNN estimates with LSTM) achieved the greatest possible robustness in estimating the extinction rate. See Figure 8 and Supplementary Appendix G, Figures 17 and 18 for details.

### Misspecification Analysis

Under model misspecification, in the naive cases, DDD MLE estimates of the carrying capacity *K* based on trees generated under BD scaled closely with the expected total number of nodes $\mathbb {E}[N_{\mathrm{nodes}}(t)]$ and were generally larger than the total number of nodes of the underlying trees. Under the naive case, 5.0% of *K* estimates fell in $[2N_{\mathrm{nodes}},5N_{\mathrm{nodes}}]$, 1.9% in $[5N_{\mathrm{nodes}},20N_{\mathrm{nodes}}]$, 5.4% in $[20N_{\mathrm{nodes}},\infty )$, and 2.6% are estimated as $\infty$. In the best-case scenario (*K* initialized at $\infty$), MLE always converged to $K=\infty$.

Neural network estimates of *K* reflect both the center of the training range (mean of sampled *K* values) and tree size ($N_{\mathrm{tips}}$, roughly half the total number of nodes). On medium- to large-sized trees, predictions concentrated near $N_{\mathrm{tips}}$, and never exceeded the training upper bound of 1000 when using the GNN alone. The Boost BT ensemble approach occasionally yielded slightly larger estimates than 1000 on large trees, but exhibited the same size-dependent bias toward $N_{\mathrm{tips}}$.

**Figure 9.**
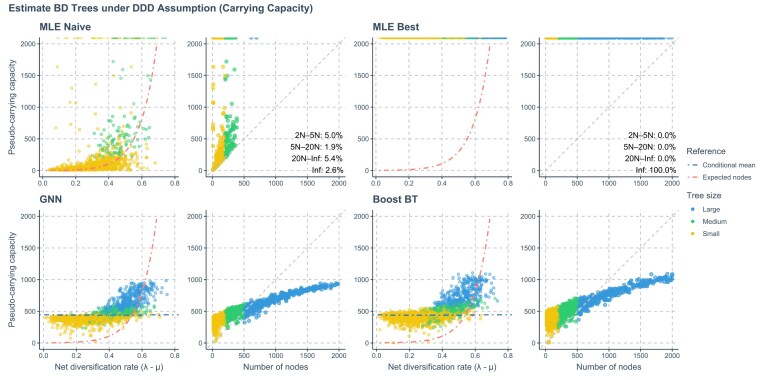
Results of applying MLE and neural networks for DDD trees to trees generated under BD (model misspecification). The predicted carrying capacities by estimation method (y-axis) are plotted against real net diversification rates $\lambda -\mu$ and numbers of observed total nodes of the trees $N_{\mathrm{nodes}}$ (*x*-axis). There are four groups of panels. Each panel group represents an estimation method. Phylogenies are categorized based on their size: yellow for small phylogenies with fewer than 200 nodes (including root, internal, and tip nodes), green for medium-sized phylogenies with 200 to 500 nodes, and blue for large phylogenies with more than 500 nodes. MLE Naive: maximum likelihood estimation results with starting point set to true speciation and extinction rates and carrying capacity *K =* 10,000. MLE Best: MLE results with starting point set to true speciation and extinction rates and carrying capacity $K = \infty$). GNN: Predictions obtained by the GNN using the phylogenies. Boost BT: Boosting strategy that LSTM corrects GNN results using branching times (BT). Red dot-dashed lines in panels referencing the expected total number of nodes of the trees under the true BD process given net diversification rate and simulation time. Blue dot-dashed lines referencing the mid-points of the parameter space of carrying capacity when training the neural networks. Gray dashed lines indicate where the number of nodes of a tree is equal to its estimated carrying capacity under wrong assumption (pseudo-carrying capacity). In the MLE result panels, predicted pseudo-carrying capacities larger than 2000 but not $\infty$ are not shown. The actual infinite predictions are displayed as points attaching to the top panel borders. Percentages shown at the bottom-right corners indicate the proportions of predicted pseudo-carrying capacities lying between certain value ranges: (1) between $2\, N_{\mathrm{nodes}}$ and $5\, N_{\mathrm{nodes}}$, (2) between $5\, N_{\mathrm{nodes}}$ and $20\, N_{\mathrm{nodes}}$, (3) between $20\, N_{\mathrm{nodes}}$ and $\infty$, and (4) $\infty$.

See Figure 9 for details.

### Empirical Data

**Figure 10.**
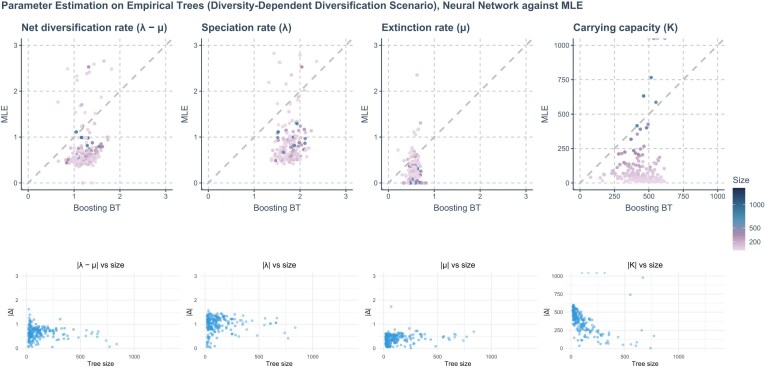
Comparison of the estimations of MLE and neural network methods (specifically, Boosting BT, which refers to using GNN to make first predictions and then using a LSTM recurrent neural network to correct for residuals) on empirical trees under a DDD scenario. Each column, arranged from left to right, focuses on a specific parameter being estimated. In the first row, x-axis represents the estimated values of the neural network. *Y*-axis represents estimated values from MLE. A gray dashed line is included in each panel to indicate where the estimations from the neural network and MLE are exactly the same. The color of the points varies from purple to blue, with the gradient representing the size of the phylogenies measured by the total number of nodes (including root, internal, and tip nodes). In the second row, the change of absolute differences between MLE and neural network predictions along tree sizes is presented. *X*-axis represents tree size, y-axis represents absolute differences.

For the empirical data sets, MLE estimates of carrying capacity were typically lower than those of the neural networks, especially in smaller phylogenies (Fig. 10). However, as the size of the phylogenies increases, MLE estimates tended to converge towards those produced by neural networks. Similarly, MLE estimates of net diversification rate also aligned more closely with neural network estimates in larger phylogenies Figure 10.

MLE generally provided a broader range of estimates on all the parameters except for carrying capacity on small phylogenies. Neural networks provided a broader range of carrying capacity estimates on small phylogenies and less frequently produced zero or near-zero estimates for extinction rates, which we often observed for MLE. We also observed that MLE sometimes produced very high values (ranging from 10,000 to infinity) for carrying capacity on empirical trees, see Figure 10 for the comparison between MLE and neural networks on empirical tree parameter estimation.

Generally, neural network estimates of all the parameters under the DDD scenario were close to the center (mean) of the distribution generated by the bootstrapping method. See Figure 15 in Supplementary Appendix F for details.

### Other Scenarios

#### BD scenario

Neural network methods outperformed MLE in accuracy on smaller phylogenies, under the BD scenario in the simulated data set (see Supplementary Appendix H, Figs. 20 and 21). Both MLE and neural network methods gave less accurate estimates on small phylogenies; this was more prominent for the MLE estimates.

Net diversification rate strongly affects predictive accuracy across all methods: larger net diversification rates typically yield larger trees and result in lower prediction errors. Extinction-to-speciation ratio has minimal impact on neural network accuracy and only a weak effect on MLE performance—higher ratios slightly increase prediction errors (see Supplementary Appendix H, Figs. 22 and 23).

**Figure 11.**
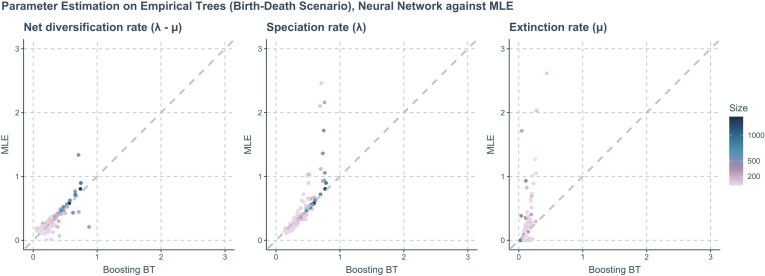
Comparing the estimations of MLE and neural network methods (specifically, Boosting BT, which refers to using GNN to make first predictions and then using LSTM recurrent neural network to correct for residuals) on empirical trees under a BD scenario. Each panel, arranged from left to right, focuses on a specific parameter being estimated. *X*-axis: Represents the estimated values of the neural network. *Y*-axis: Represents estimated the values from MLE. A gray dashed line is included in each panel to indicate where the estimations from the neural network and MLE are exactly the same. The color of the points varies from purple to blue, with the gradient representing the size of the phylogenies measured by the total number of nodes (including root, internal, and tip nodes).

On empirical phylogenies, similar to the DDD scenario, neural network methods seldom produced zero-estimation of extinction rate, unlike MLE, which often produced zero or near-zero estimates for the extinction rate. Neural networks tended to give estimates within the parameter space of the training data set. They predicted conservative speciation and extinction rates yet were highly consistent with MLE estimation on the net diversification rate. The consistency of prediction increased on larger empirical phylogenies. See Figure 11 for details.

#### Protracted BD scenario

MLE and neural network methods did not perform well on estimating parameters under the PBD scenario; MLE estimates were generally less accurate, but neural networks also failed to predict the parameters as all the parameter estimates were close to the mid-points of corresponding parameter spaces (Supplementary Appendix I, Fig. 24). However, there were exceptions: neural networks seemed to perform better on the speciation rate of the incipient species ($\lambda _3$) and on the mean duration of speciation ($\tau$) when the true value is between 0 and 2.

MLE estimates became significantly inaccurate as phylogenies became smaller (Supplementary Appendix I, Fig. 25); it is also noticeable that MLE estimates of the speciation completion rate ($\lambda _2$) are very inaccurate, especially when the phylogenies are large. A general pattern is that both MLE and neural network methods achieved more accurate estimates on phylogenies with higher true values of the mean duration of speciation (Supplementary Appendix I, Fig. 26).

## Discussion

We have developed an ensemble learning neural network approach that matches and sometimes outperforms the accuracy and robustness of MLE for estimating phylogenetic tree parameters. Our approach leverages different classes of neural networks by learning from the phylogenies, their branching times, and their summary statistics simultaneously.

When trained, our neural networks can compute estimates faster than MLE on larger phylogenies as computation time is less affected by increases in phylogeny size. We considered boosting strategies most effective in eliminating systematic prediction errors in neural network estimates. Among them, Boost BT (which corrects GNN results using LSTM) achieved overall best performance, which is comparable to, or even surpassing, the best-case MLE, in terms of accuracy and robustness. We observed that generally the performance of the naive MLE was second-worst (Boost BT+SS was worst). Interestingly, some phylogenies, such as small trees and those shaped by relatively weak effects, pose significant challenges to both MLE and neural network methods.

Previous neural network methods applied to phylogenies have experimented with various architectures such as CNN1D, GNN, and LSTM (Voznica et al. 2022; Lajaaiti et al. 2023; Lambert et al. 2023). The deep learning architectures we employed differ from those used in prior studies, making direct comparisons challenging. However, the comparison we performed against CNN1D reveals that the neural network architectures we have tested share similar patterns. Additionally, while previous research focused on BD (Lambert et al. 2023) and trait-state-dependent models (Lajaaiti et al. 2023; Thompson et al. 2024), our approach is novel in its application to models such as DDD and PBD from a neural network perspective. Despite these differences, our findings align with recent studies in underscoring the potential of neural networks to infer diversification processes, offering a viable alternative to mathematically complex methods. Future research could attempt conformalized prediction approaches to quantify uncertainty in the NNs, as these have been shown to be quite efficient (Romano et al. 2019).

### Rethinking Neural Networks

Although performance was equal or potentially better than MLE, the neural network approach is not without shortcomings. The neural networks often defaulted to predicting values close to the mid-point of the true parameter space of the training data set, indicating that they struggle to extract meaningful features from the data set. This predictive performance in the absence of information is similar to that of Bayesian analysis, in which the posterior is equal to the prior when the likelihood function is flat. The similarity also depends on the choice of loss function, for example, when the MSE is chosen, the neural-network regression setting is similar to maximum-likelihood estimation with independent and Gaussian observation noise. Under these assumptions the network converges on the conditional mean of the target variable. In our study we adopted the Huber loss, which retains this connection while introducing heavier tails to reduce the impact of outliers, thereby behaving much like MSE (Casella and Berger 2002; Bishop and Nasrabadi 2006). This conservative prediction strategy minimizes overall error compared with random guessing. Examples can be found in the GNN predictions of carrying capacity from simulated DDD trees, the DNN estimates of the speciation and extinction rates (Figs. 4–6, but see Supplementary Appendix M for a detailed investigation of possible under-performance of DNN on the summary statistics) and most neural network predictions of PBD related parameters (Supplementary Appendix I, Fig. 24), especially for smaller phylogenies.

This behavior, while effective in reducing apparent error metrics, can skew our understanding of a neural network’s performance particularly when the focal parameter space is relatively narrow. Neural networks may consistently show smaller overall error compared with MLE, because the latter has no prior knowledge of the limits of the parameter space, which would lead to a false impression of better accuracy of the neural networks. We therefore recommend performing case-specific residual analyses on the neural network predictions and the MLE estimates, which are often overlooked or over-simplified. This behavior also dominates neural network predictions on small trees under model misspecification, where neural networks trained on DDD trees try to recover the carrying capacity *K* from BD trees (see Fig. 9). If robust prior bounds on the admissible parameter space are available, however, neural networks can leverage the knowledge and achieve better performance.

To mitigate the impact of training data sets that may insufficiently represent the parameter space, we can first train the neural networks using a relatively narrow training data set and generate parameter predictions from empirical data; then retrain the networks with a broader training data set that covers a larger or different portion of the parameter space. By comparing predictions of the neural networks from the two training data sets on the same empirical data, we can evaluate whether and how strongly our predictions depend on the parameter ranges represented in the training data. This procedure is similar to a prior sensitivity analysis in Bayesian inference. Generally, we recommend to train the neural networks with as large a data set (sample size) and as broad a parameter space as possible, unless there is prior knowledge of the parameter space being estimated.

Improving neural network predictions that are close to the mean is unlikely to be achieved by increasing the amount of training data: we did not observe major performance improvement when changing the size of the data sets (from 1000 to 100,000 phylogenies per data set). Instead, one might consider increasing the complexity of the network architecture, such as increasing their depth or adapting the scale of the hidden nodes (Zhang et al. 2021), but note that this can only work if there is additional signal in the data (as discussed in the next section) and this will typically require more data.

Although potentially beneficial, increasing the depth can also harm predictive power. In particular for GNNs, increasing their depth may lead to “over-smoothing” and “over-squashing.” Over-smoothing causes node features to become increasingly similar as more layers are added (Li et al. 2018), leading to a loss of distinct node embeddings across different clusters. Over-squashing involves the compression of expansive node information through bottleneck edges into a fixed-size vector, which is problematic in graphs with large diameters and long-range dependencies (Alon and Yahav 2021), for example, phylogenies. Both issues degrade node representations and distort information flow, making deeper GNNs potentially less effective than shallower ones (Dwivedi et al. 2022). Moreover, over-smoothing and over-squashing are intrinsically linked, creating a trade-off that cannot be easily resolved (Giraldo et al. 2023).

In our analyses, we observed that increasing the number of GraphSAGE layers beyond three in the differentiable pooling architecture destabilized the training process and reduced the accuracy of estimates on validation data sets, introducing more outliers. We therefore opted to maintain two layers throughout our study. We explored newer algorithms designed to mitigate deep GNN issues (Li et al. 2018; Chen et al. 2020; Gravina et al. 2022), but found that these deeper architectures performed worse than our differentiable pooling approach with fewer layers. For DNN and LSTM, we also experimented with more complex architectures, different activation functions, and various hyper-parameter optimizations but failed to achieve better performance.

### Fundamental Problems with Phylogenies

The lack of improvement when changing the amount of training data or the network architecture suggests that the real challenges of estimating parameters might not lie in the architecture of the networks, but might instead be attributed to underlying weak or absent phylogenetic signals. Whenever this is the case, we expect similarities in inaccuracies of both MLE and neural network approaches. This occurs, for example, for the carrying capacity when it is high and thus has a weak effect (measured by $(\lambda - \mu )/K$). Here, the phylogeny is typically not near the carrying capacity, allowing the number of species to grow (almost) unbounded. This may result in carrying capacity estimates that are arbitrarily high, especially in the MLE methods. The PBD scenario is known to present difficulties in reliably recovering parameters with MLE (Etienne et al. 2014) and we find similar poor performance with neural networks. A second case where accurate parameter estimation is complicated occurs when extinction processes erase critical information (Louca and Pennell 2021), as observed in the decline of estimation robustness associated with increasing extinction rate (see Fig. 8 for a comparison of errors across different values of $\mu$: the prediction errors tend to increase with $\mu$, particularly when $\mu$ constitutes a larger proportion relative to $\lambda$).

More generally, small phylogenies tend to contain less information than large ones. In our results, we see that estimation accuracy and robustness decline with decreasing size of the phylogenies. This trend is observed across both MLE and neural network methods. In the BD and PBD scenarios, where data sets have greater variability in phylogeny sizes, poor estimations for small trees could be explained by both low information content, or under-representation of such trees. After re-balancing tree sizes (Supplementary Appendix K), the same patterns occurred and they are therefore unlikely to be a result of under- or over-representation of different phylogeny sizes, but instead reflect low information content of small trees (compare Figs. 20 and 33). Empirical phylogenies usually offer only one single tree per one exact process, so formal goodness-of-fit tests have low power regardless of the estimator. In such single-realization settings, the safest strategy is to compare several estimators, if available; large discrepancies are a warning sign of misspecification.

### Confronting the Empirical Phylogenies

The processes of evolution within natural systems are often unknown. Determining the “true parameters” of an empirical phylogeny is challenging, even when they meet theoretical assumptions, making it difficult to evaluate which tool provides more accurate estimates. Therefore, choosing the right tool is crucial.

With neural networks, it is possible that the true parameter value is not part of the assumed parameter range for simulating the training data. In such cases, neural network accuracy decreases notably, as shown in our second supplementary analysis (Supplementary Appendix L). We also noticed that when comparing the estimates of MLE and neural network methods on the empirical phylogenies (see Figs. 10 and 11), MLE estimates spread wider than the neural networks (e.g., our BD training data set comprises phylogenies simulated using speciation rate between 0 and 0.8 and extinction rate between 0 and 0.72, where our neural networks never predict speciation rates larger than 0.8 or extinction rates larger than 0.72, see Fig. 11, similar results can be found under the DDD scenario in Fig. 10). Expanding the training data set’s parameter space can resolve the generalization issue (we expanded our training data sets several times in the experiments), but this approach requires significantly more computational resources for both simulation and training of the neural networks.

Our supplementary study on generalizability and data completeness (explained in Supplementary Appendix L) also reveals that neural networks tend to provide more accurate estimates of speciation and extinction rates from complete phylogenies (with both extant and extinct tips) than from phylogenies with only extant species under the BD scenario. This increase in accuracy was not observed in the DDD scenario. Although complete phylogenies offer a broader picture and more contextual information, obtaining them is challenging because it is nearly impossible to account for all extinct species.

Our analyses indicate that GNN is more robust but more prone to systematic errors (GNN achieved the greatest possible robustness in estimating the speciation rate and carrying capacity among neural network methods). We show that using GNN as a base and other neural networks like LSTM to enhance GNN might effectively combine the advantages of different methods and information sources, thus strengthening overall generalization ability. Our boosting methods (e.g., Boost BT) perform the best in this context.

In conclusion, when applied with caution, we expect that neural network methods can be applied to diversification scenarios where MLE is absent or non-tractable, as our best-performing neural network method showed comparable or even better performance to the best-case MLE. Our neural networks particularly performed better than MLE in terms of accuracy and robustness on small phylogenies and can be significantly faster when estimating very large phylogenies. Thus, if properly trained, neural network methods may substitute for or at least cross-reference with MLE estimates where they exist.

## Supplementary Material

syaf060_Supplemental_Files

## Data Availability

Our research outputs from a complete run, which includes simulation, maximum likelihood estimation, neural network training, validation, and testing from our computing cluster are deposited through Dryad at https://doi.org/10.5061/dryad.2v6wwpzx7
