# Supplementary material for: Parameter Estimation from Phylogenetic Trees Using Neural Networks and Ensemble Learning
Source: Syst Biol. 2025 Sep 3;75(2):344–65. doi: 10.1093/sysbio/syaf060 (PMC13017624; doi:10.1093/sysbio/syaf060)
Supplement: syaf060_Supplemental_Files [file syaf060_supplemental_files.zip › final_appendix.pdf]

APPENDIX

A. Data Transformation Protocol

Protocol Transforming Phylogenies for Graph Neural Network

Phylogenetic trees are usually stored in the "phylo" data format in R. This data format is not directly compatible with GNN implementations. To facilitate graph convolutional operations, we transformed phylogeny from a "phylo" object into three major components: adjacency list, node feature matrix and graph attributes (see Figure 2). The adjacency list contains information on the connectivity between nodes and tips, the node feature matrix contains distances between nodes and tips, and the graph-level attributes include the ground truth model parameters used to generate the phylogenies. These components are stored in separate tensors. In machine learning, a tensor is a mathematical object that generalizes scalars, vectors, and matrices to higher dimensions, allowing complex operations to be performed efficiently on multi-dimensional arrays.

Adjacency List In the context of a phylogenetic tree, tip nodes usually represent taxonomic units such as species, while root nodes and internal nodes represent the points where two taxonomic units depart from each other. An edge in a phylogenetic tree represents the hierarchical connection between two nodes (the ancestor and the descendant), and as such describes the evolutionary relatedness between taxa. Each root node, internal node and tip node in an R "phylo" object is indexed sequentially, each edge is also sequentially indexed independently of node indices. The sub-list "edge" of a "phylo" object contains the adjacency list of a phylogenetic tree which describes the relationships between nodes. Each row of the adjacency list represents an edge, the first column contains the index (or numbering) of the ancestor node, and the second column contains the index of the descendant node.

This data structure effectively captures the tree's branching pattern, showing how each taxon (or node) is connected to others. The adjacency list in "phylo" object uses a

"1-based" indexing in R, we therefore element-wise deduct 1 from the list to convert it into "0-based" indexing which is compatible with the python environment.

We output the converted adjacency list within the "phylo" object as the adjacency list $\mathcal{E}$ of the graph representation, in PyTorch Geometric, which is conventionally named as "data.edge_index". We store $\mathcal{E}$ as a "torch.long" long integer type tensor and transpose it such that it has shape $[2, num\_edges]$, where "num_edges" is the number of edges in the "phylo" object. This tensor has two dimensions. This way, the connections between nodes in the transformed graph are all single-directional, from the ancestor nodes to their descendants (if any). Training the GNN with graphs of non-directed edges gives no performance advantage, according to our tests in phylogenetic tree parameter estimation tasks. Single-directional data structure can save GPU memory and reduce the computation complexity.

Node Feature Matrix In a "phylo" object in R, the "edge.length" sub-list defines the lengths of the edges in the phylogenetic tree. In a phylogenetic context, these lengths often correspond to evolutionary distances, time, or genetic change. "edge.length" is a numeric vector where each element corresponds to the length of the edge as defined in the adjacency list. The order of lengths in the "edge.length" vector aligns with the order of edges in the adjacency list.

For each tree, we aggregate information contained in "edge.length" to a node feature matrix. Each row of the matrix represents features contained in a node. The first column contains the edge length from a node to its direct ancestor node, the second and the third columns contain the edge lengths from a node to its two daughter nodes. We pad the row of the root node with an 0 in the first column as it has no ancestor. We also pad the rows of the tip nodes with two 0s in the second and the third columns as they have no descendants. The row order of feature matrix aligns with the order of edges in the adjacency list.

We output the node feature matrix of each tree as the node feature matrix $\mathcal{X}$ of the graph representation, in PyTorch Geometric, this is conventionally named as "data.x". We

store $\mathcal{X}$ as a "torch.float" floating point type tensor, it has shape $[num\_nodes, num\_node\_features]$, where "num_nodes" is the number of nodes (including tip nodes) in the "phylo" object and "num_node_features" in our case is 3, i.e. the phylogenetic distances from a node to its ancestor (if any) and two descendants (if any). This tensor has two dimensions. We do not store the phylogenetic distance information in edge features because GCN operators will eventually pass and aggregate the edge features into each of the node. Our data structure is simpler and so is the GNN architecture.

Graph-Level Attributes as Training Targets We store all the parameters used to simulate a tree (ground truth values) in the graph-level attributes $\mathcal{Y}$. These can have arbitrary length, which should be consistent with the number of the parameters to be estimated (the three diversification scenarios, BD, DDD and PBD, have different number of parameters). We store graph-level attributes as a "torch.float" floating point type tensor with length of the number of parameters we want to predict for each type of the phylogenetic tree. In PyTorch Geometric, graph-level attributes can be named as "data.y". The graph-level attributes are used as training targets to compute loss (see Appendix B for the definition of loss).

Protocol Transforming Summary Statistics for Dense Neural Network

The summary statistics of a phylogeny are represented by a 1D vector, so the protocol for DNN is straightforward: we convert the vector into a tensor containing floating type data, with the shape [num_stats], where "num_stats" denotes the total number of statistics. This tensor has only one dimension. This conversion guarantees that each tensor is associated with its respective tree, with all contained statistics maintaining their original order. Within the PyTorch Geometric framework, these statistics are encapsulated as "data.stats" for each tree. When using DNN alone to estimate parameters

from the summary statistics, the ground truth values of the parameters of the trees are stored in the same way as the graph-level attributes, as model training targets. When using DNN with other neural networks (e.g. in stacking and boosting strategies), they share the same ground truth values which are the graph-level attributes.

Protocol Transforming Branching Times for Recurrent Neural Network

To address the varying lengths in branching times across different phylogenetic trees, we standardize these sequences by padding them to match the length of the longest branching time sequence. This is achieved by appending zeros to the shorter sequences until they match the predefined maximum length. The padded sequences are stored in tensors containing floating type data. As the original branching times do not contain zero values, this padding strategy allows us to distinguish between original data and padding. Consequently, we can pass masks of the sequences to the LSTM, which indicates the positions of the paddings, making LSTM concentrate only on the informative portions of the sequences, thereby optimizing its performance. When using LSTM alone to estimate parameters from the branching times, the ground truth values of the parameters of the trees are stored in the same way as the graph-level attributes, as model training targets. When using LSTM with other neural networks (e.g. in stacking and boosting strategies), they share the same ground truth values which are the graph-level attributes.

An example of data components extracted and computed from a phylogenetic tree is illustrated in Figure 12.

Phylogenetic Tree

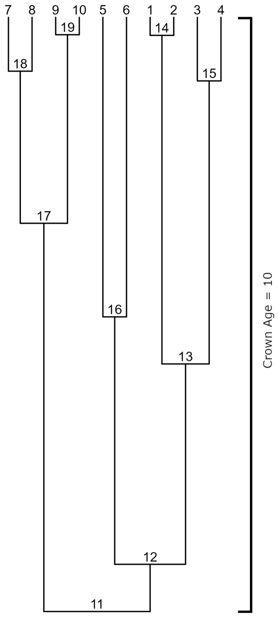

Adjacency List

Start	End
11	12
12	13
13	14
14	1
14	2
13	15
15	3
15	4
12	16
16	5
16	6
11	17
17	18
18	7
18	8
17	19
19	9
19	10

Feature Matrix

	Anc	Des1	Des2
1	0.152	0	0
2	0.152	0	0
3	0.535	0	0
4	0.535	0	0
5	2.52	0	0
6	2.52	0	0
7	0.453	0	0
8	0.453	0	0
9	0.146	0	0
10	0.146	0	0
11	0	0.397	3.267
12	0.397	1.686	2.082
13	1.686	2.765	2.382
14	2.765	0.152	0.152
15	2.382	0.535	0.535
16	2.082	2.52	2.52
17	3.267	1.28	1.587
18	1.28	0.453	0.453
19	1.587	0.146	0.146

Branching Times

	Time
11	5
12	4.603
13	2.917
16	2.52
17	1.733
15	0.535
18	0.453
14	0.152
19	0.146

Summary Stats (first 16)

Stat	Value
area_per_pair	5.378
average_leaf_depth	3.4
avg_ladder	0
avg_vert_depth	2.632
b1	6.333
b2	3.25
beta	10
blum	6.004
cherries	5
colless	4
colless_corr	0.111
colless_quad	8
crown_age	5
diameter	7
double_cherries	2
eigen_centrality	0.436
…	…

Fig. 12. Example of a simulated phylogenetic tree and its derived input data for the neural networks. The first panel is a simulated tree with extant taxa (tips) under the diversity-dependent diversification scenario, its nodes (including root, internal and tip nodes) are labeled. The second panel shows the corresponding adjacency list, in which each parent-child relationship is presented in a row by an Start $\longrightarrow$End format. The third panel shows the corresponding feature matrix, with one row per node and three columns (Anc, Des1, Des2) corresponding to the branchlength values toward the parent and up to two descendant nodes, the row indices are only for visual assistance, not included in the input data. The fourth panel shows branching times for each internal node, listing node ages, the row indices are not included. The fifth panel shows a subset of the first 16 summary statistics computed, the final row ("...") indicates continuation of the full statistic vector, the "Stat" column is not included in the input data.

B. Total Loss

Total loss comprises three key components: Huber loss, link prediction loss and entropy of regularization. Huber loss was used for optimizing regression accuracy while the remaining components focused on alleviating a possible issue where GNN can be hard to train, if incorporating the differentiable pooling method (Ying et al., 2018).

The Huber loss (Huber, 1992) for vectors y and $\hat{y}$, each with n elements, computed as the average loss across all elements, is given by:

$$L_\delta(\mathbf{y}, \hat{\mathbf{y}}) = \frac{1}{n}\sum_{i=1}^{n}\begin{cases}\frac{1}{2}(y_i - \hat{y}_i)^2 & \text{for } |y_i - \hat{y}_i| \leqslant \delta, \\ \delta(|y_i - \hat{y}_i| - \frac{1}{2}\delta) & \text{otherwise,}\end{cases} \tag{B.1}$$

where $\mathbf{y}$ is the true value vector comprising the ground truth parameters used for simulating a phylogenetic tree, $\hat{\mathbf{y}}$ is the predicted value vector comprising the parameter predictions, y_i and $\hat{y}_i$ are the i-th elements of $\mathbf{y}$ and $\hat{\mathbf{y}}$ respectively, n is the number of elements in the vectors $\mathbf{y}$ and $\hat{\mathbf{y}}$ and δ is the threshold parameter that defines the transition from squared to linear loss (here loss refers to the difference between ground truth and predicted values). In our research, we set $\delta = 0.8$ for all the training sessions, making the neural networks more sensitive to smaller errors and more robust to outliers .

The total loss L is given by

$$L = L_\delta(\mathbf{y}, \hat{\mathbf{y}}) + L_{\mathrm{LP}} + L_{\mathrm{E}}, \tag{B.2}$$

where L_{LP} is the link prediction loss and L_{E} is the entropy of regularization, see Ying et al. (2018) for their definitions.

C. Neural Network Architecture

For the graph neural network, we used GraphSAGE (Hamilton et al., 2017), a sample-and-aggregate graph convolutional neural network, to capture a graph-level representation. GraphSAGE has achieved strong performance of learning from large graphs. We use graph neural network (GNN) to refer to the graph neural network approach which incorporates GraphSAGE.

GNN is mainly assembled from five GNN modules (see Figure 1-C for five blocks of boxes in yellow and orange colors). Each module comprises the same number of GraphSAGE operators (Hamilton et al., 2017), where the number of layers (GraphSAGE operators, as illustrated by the number of combined boxes within each GNN modules in Figure 1-C) $N_{\mathcal{L}} = 1, 2, \ldots, 6$. Each GNN operator is accompanied by a Batch Normalization for 1D Inputs (BatchNorm1d, not shown in Figure 1) operator (Ioffe and Szegedy, 2015) and then a Gaussian Error Linear Units (GELU, as illustrated by the orange bands within the yellow boxes in Figure 1-C) activation function (Hendrycks and Gimpel, 2016). The GraphSAGE operators facilitate the convolution operation over graphs, capturing both local node features and their neighborhood information. The BatchNorm1d operator is commonly employed in neural networks to stabilize and accelerate the training process. The GELU activation layer is used for introducing non-linearity into the data. Learned features from all the GraphSAGE operators within a module are collected and concatenated. Larger $N_{\mathcal{L}}$ will result in the GNN modules to aggregate information into each node from its more distantly connected neighbors. According to our experiments, the optimal case is $N_{\mathcal{L}} = 2$, all figures and results relating to GNN were reported on the optimal case.

The graph-learning process also involves graph coarsening operations. We incorporated the differentiable pooling (DiffPool hereafter) technique to better learn hierarchical representations of the graphs. DiffPool can aggregate graph nodes into clusters after each operation. It facilitates graph coarsening and captures intricate hierarchical

structure, which makes it particularly suitable for graph-level tasks (Ying et al., 2018). In the first coarsening operation, the graph data inputs are passed to two GNN modules (pooling and embedding, see Figure 1-C for the blocks marked as "GNN pool1" and "GNN embed1"). The pooling group reduces the graph size, while the embedding group captures the node features. The filtered data from each GraphSAGE operator are concatenated (see Figure 1-C for the blocks of boxes marked as "concat1") then passed to a DiffPool layer (see Figure 1-C for the red box marked as "diff-pool1"), which finalizes the first coarsening operation. The second coarsening operation is applied in the same way as the first (as represented by "GNN pool1", "GNN embed2", "concat2" in Figure 1-C), and the outputs from the second DiffPool layer ("diff-pool2" in Figure 1-C) are passed to the final (fifth) GNN module ("GNN embed3" in Figure 1-C). The nodes in a graph are dynamically clustered and reduced after each coarsening operation. The coarsening ratio at each operation is determined by a pre-set DiffPool pooling ratio. Let $N_{\text{coarsened}}$ represent the number of nodes in the coarsened graph and N_{original} the number of nodes in the original graph. The DiffPool pooling ratio ρ_{pool} is given by $\rho_{\text{pool}} = \frac{N_{\text{coarsened}}}{N_{\text{original}}}$. Throughout the study, we used a manually optimized value $\rho_{\text{pool}} = 0.25$. This is a manually optimized hyper-parameter.

After the final GNN module, the outputs are concatenated ("concat3" in Figure 1-C) and transformed by a global mean pooling operation (red ball "M" in Figure 1-C) to create a final graph representation. This graph representation is passed to a readout layer group ("readout" as represented by light blue boxes in Figure 1-C) consisting of two linear layers to perform graph-level regression which ultimately outputs a vector of n predicted parameters ("pred" as represented by a purple box in Figure 1-C). Only the first linear layer is followed by GELU (see the orange band of the first linear layer). All the linear layers incorporate dropout operations with a pre-set dropout ratio to prevent over-fitting and to utilize as many neuron connections as possible. Let ρ_{dropout} represent the probability p of disabling a connection between an input node and a hidden node of a

linear layer in each epoch. The dropout ratio ρ_{dropout} is simply given by $\rho_{\text{dropout}} = p$. Throughout the study, we used a commonly picked value $\rho_{\text{dropout}} = 0.5$. This is a manually optimized hyper-parameter.

DNN's major component is a stack comprises 5 linear layers ("DNN stack" in Figure 1-A), each followed by a BatchNorm1D (not shown in figure) and a GELU (the orange band within the boxes). All the linear layers within the stack incorporate dropout operations with $\rho_{\text{dropout}} = 0.5$. Learned features from all the linear layers within the stacks are collected and concatenated ("concat" in Figure 1-A). A single linear readout layer ("readout" in Figure 1-A) outputs n predicted parameters ("pred" in Figure 1-A). According to our experiments, stacking more linear layers gives no substantial improvement to the performance.

LSTM's major component is a stack of 5 LSTM recurrent neural network layers ("LSTM stack" in Figure 1-B). The final hidden state from the last recurrent neural network layer is processed by a linear layer with $\rho_{\text{dropout}} = 0.5$ accompanied by a GELU ("linear" in Figure 1-B), then passed to a single linear readout layer ("readout" in Figure 1-B) that outputs n predicted parameters ("pred" in Figure 1-B). According to our experiments, stacking more recurrent neural network layers provides no substantial improvement to the performance.

The hyper-parameters not mentioned are set by their default values. The dimensions of the boxes do not map to any hyper-parameter settings, they are set for the best visual effect. The values below the boxes indicate their respective number of hidden neurons, their input and output neurons are not shown in the figure, they can be found in the configuration files in our GitHub repository "eveGNN" (Qin, 2023).

D. Ensemble Learning

With bagging, we trained GNN, DNN and LSTM independently ("GNN", "DNN" and "LSTM" blocks of boxes in Figure 3-Bagging), translated their outputs to parameter predictions through their own readout layers (three "readout" boxes next to the neural networks and three "pred" boxes next to the readout layers in Figure 3-Bagging) and then aggregated the predictions (red ball "A" in Figure 3-Bagging). We experimented with four aggregation methods: taking the mean, median, max and min values among the three predictions. We also recorded the individual predictions without aggregation.

With stacking, we trained GNN, DNN and LSTM simultaneously ("GNN", "DNN" and "LSTM" blocks of boxes in Figure 3-Stacking) but without their own readout layers. We combined the features from DNN, the LSTM's final hidden state, and GNN's graph representation and fed to a meta-learner ("meta-learner" in Figure 3-Stacking) comprising linear neural network layers that learns to best readout parameter predictions from these combined outputs.

With boosting, there can be different pathways. In our illustration, GNN, DNN and LSTM were trained sequentially to iteratively correct residuals. For example, firstly, the GNN ("GNN" in Figure 3-Boosting) is trained from the graphs to make the initial predictions ("readout" and then "pred0" in Figure 3-Boosting) and from predicted and ground truth values of the parameters we computed the residuals ("res1" in Figure 3-Boosting)secondly, the DNN ("DNN" in Figure 3-Boosting) is trained to predict these residuals from the summary statistics ("readout" and then "pred-res1" in Figure 3-Boosting), learning to correct the GNN's errorslastly, the LSTM ("LSTM" in Figure 3-Boosting) is trained to predict the residuals of the residuals ("readout" and then "pred-res2" in Figure 3-Boosting), which is the initial predictions minus the predicted residuals by the DNN, from branching times, to further improve the predictive accuracy. Finally, we subtracted the two residual terms from the initial predictions (red ball "S" in Figure 3-Boosting) to make the corrected predictions.

E. Comparison between MLE Optimizers

On the phylogenies from the diversity-dependent diversification (DDD) dataset, we compared between three approaches: "Simplex", "Subplex" and "DEoptim". Simplex is a derivative-free optimization method that uses a simplex of solutions to iteratively explore and adjust within the parameter space, suitable for non-smooth objective functions but potentially slow for high-dimensional problems (Morgan and Deming, 1974). Subplex is an enhancement of the Simplex method, Subplex breaks high-dimensional optimization into smaller subproblems, each optimized using Simplex techniques, providing improved efficiency and effectiveness in complex parameter landscapes (Rowan, 1990). DEoptim (Differential Evolution) is a more recent population-based algorithm that applies evolutionary strategies such as mutation, crossover, and selection to efficiently navigate and optimize multimodal and complex objective functions (Ardia et al., 2010).

All three MLE methods encountered consistent optimization challenges, likely due to numerical issues related to machine precision limits or unexpected negative values during matrix operations. From a random sample of 2000 DDD phylogenies, the completion rates for each method were as follows: Simplex achieved 1966 completions from true parameter starts and 1910 from random starts; Subplex completed 1681 from true starts and 1612 from random starts; DEoptim finished 1122 from true starts and 999 from random starts. It is more difficult to estimate parameters from random starts, comparing to true starts.

For all the three optimization approaches, -1 will be returned as a parameter estimation if the likelihood becomes too small in the searching process. This means that the algorithm cannot find optima given the initial starting point of the parameters. It is highly possible that the unfinished estimations consisted of inaccurate or even -1 values. The comparison between MLE optimizers can be skewed due to less completion rate of the Subplex and DEoptim results.

In instances where optimization starting points were randomly set, a significant number of outcomes were trapped at local optima, failing to achieve global optima and

often leading to inaccurate parameter estimates. This issue was less prevalent when starting points were the true parameters. For visual reference, see Figure 13 and Figure 14. Notably, in DEoptim's best-case scenarios, estimation accuracy deteriorated significantly on larger phylogenies, as shown in the last row of Figure 14.

In the best-case scenarios, all MLE methods tended to yield more accurate estimates on larger phylogenies, while in naive cases, larger phylogenies posed challenges. However, all MLE methods generally performed better with larger trees, and all displayed similar trends of bias. We calculated the strength of the carrying capacity effect with the formula $(\lambda - \mu)/K$, where λ is the true speciation rate, μ is the true extinction rate, and K is the true carrying capacity.

Subplex was the fastest among the tested algorithms, Simplex and DEoptim were slower. Simplex, although slower, completed the most computations and did not show a definitive performance disadvantage compared to Subplex or DEoptim. For this reason, in all comparisons between MLE and neural network methods, we consistently used results from the Simplex optimizer due to data coverage and reliability.

With the Simplex optimizer, we also compared the estimates between using two integration methods: matrix exponentiation ("analytical") and numerical integrator ("odeint::runge_kutta_cash_karp54"). To ensure that the MLE estimates shown in the figures are reliable, we always adopted the results of the numerical integrator and excluded the data points on which the discrepancy of estimates between two integration methods is larger than 10% relatively. See

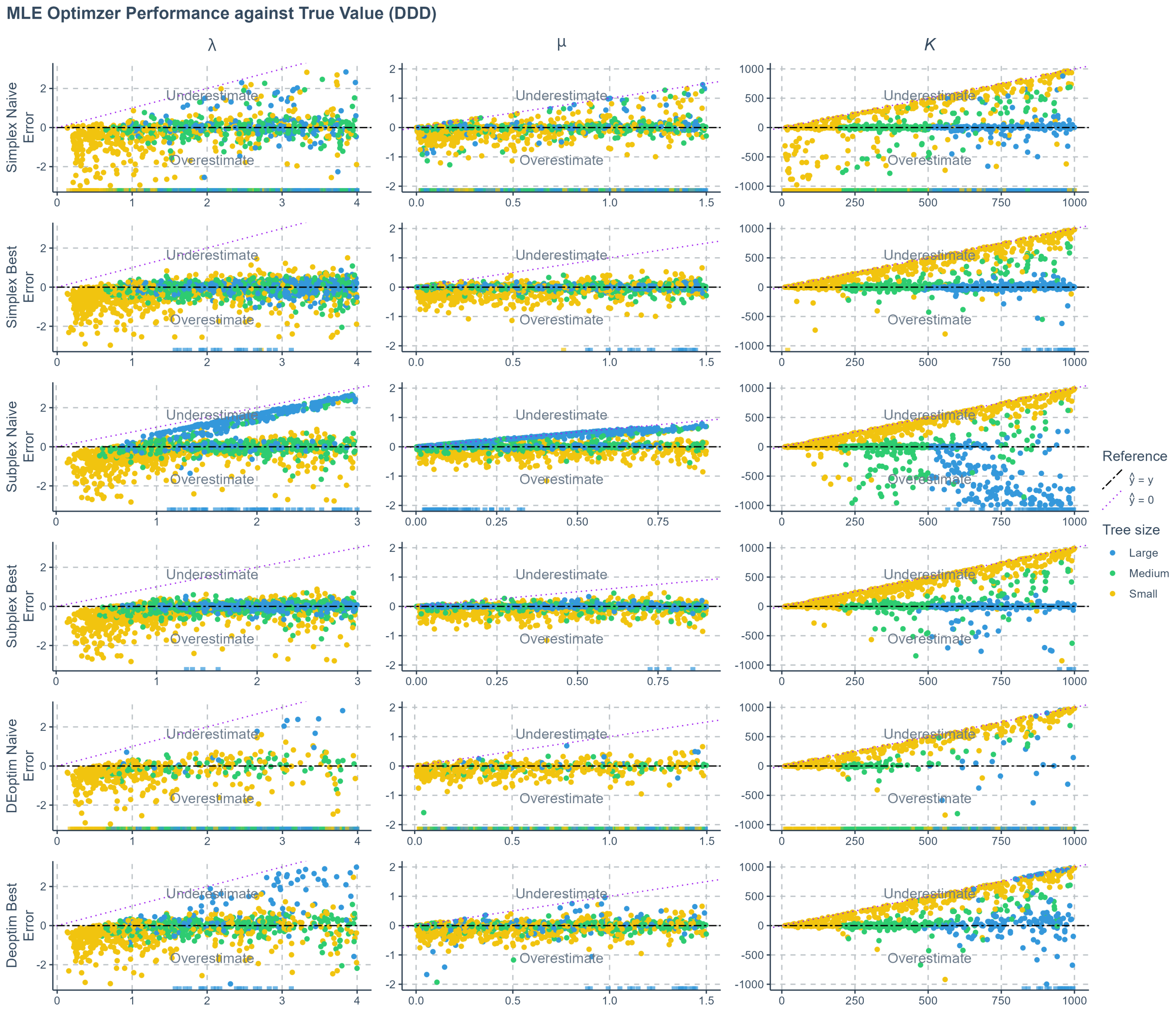

Fig. 13. Error of maximum likelihood estimation using Simplex, Subplex and DEoptim optimzers applied to phylogenies simulated under a diversity-dependent diversification scenario, against true values. For each optimzer there were two cases. The best case (Best) refers to using the true parameter values as the starting points for the searching process; the naive case (Naive) refers to using randomly sampled values from the true parameter space as the starting points. The errors shown (y-axis) are the differences between the true parameters (x-axis) used to simulate the phylogenies and the values estimated by each method. Each row represents a method, and each column corresponds to the results for one specific parameter. Phylogenies are categorized based on their size: yellow for small phylogenies with fewer than 200 nodes (including root, internal, and tip nodes), green for medium-sized phylogenies with 200 to 500 nodes, and blue for large phylogenies with more than 500 nodes. Data points close to purple dotted lines ($\hat{y} = 0$) in MLE result panels indicate near-zero estimates. Black two-dash lines indicate accurate estimates ($\hat{y} = y$ where y denotes the true parameter value). Small squares spreading along the x-axis signify optimization failures. Extremely deviating estimates are not shown in the figure. λ: Speciation rate. μ: extinction rate. K: carrying capacity.

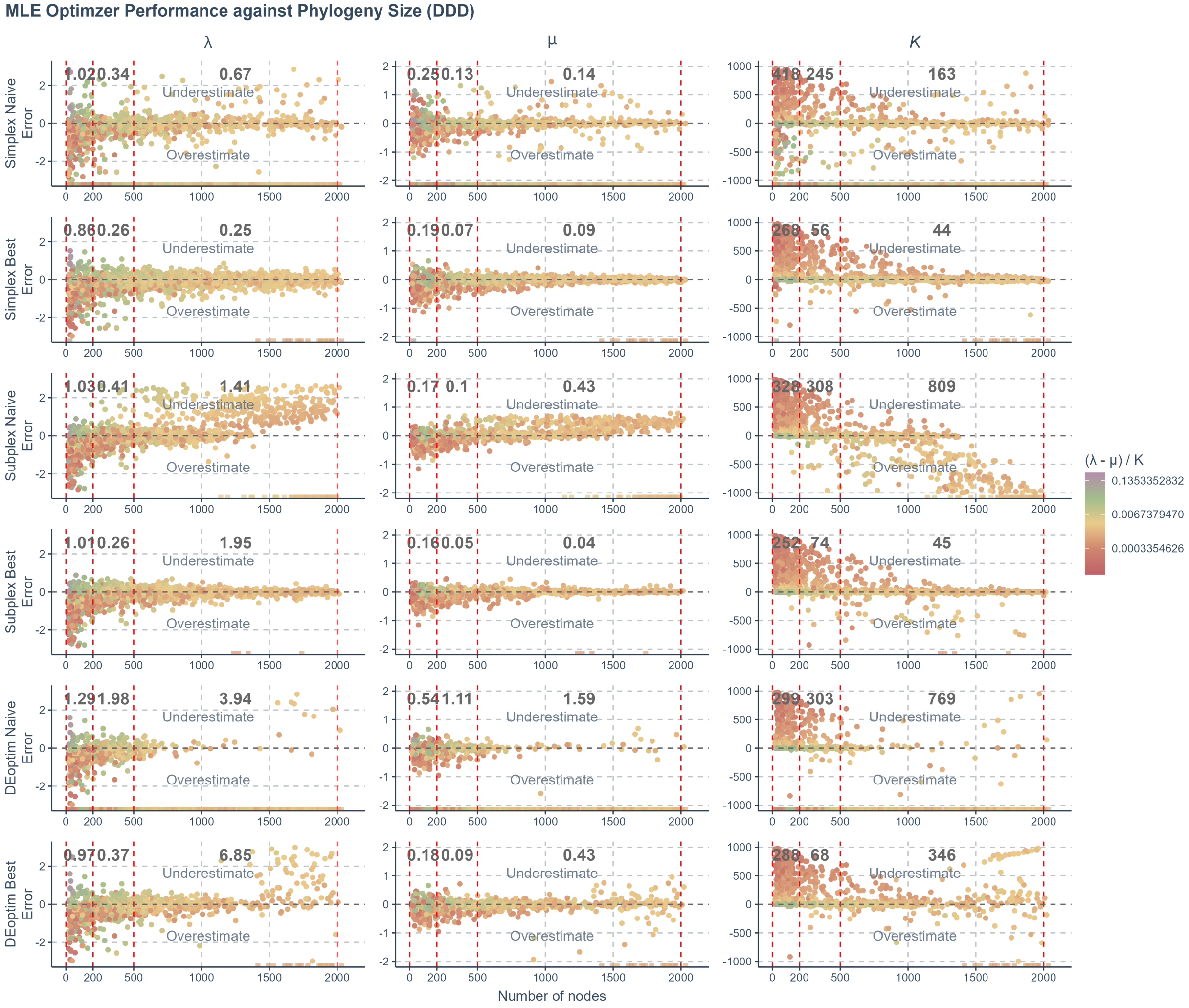

Fig. 14. Error of maximum likelihood estimation using Simplex, Subplex and DEoptim optimzers applied to phylogenies simulated under a diversity-dependent diversification scenario, against the total number of nodes (including root, internal, and tip nodes) in the phylogenies. For each optimzer there were two cases. The best case (Best) refers to using the true parameter values as the starting points for the searching process; the naive case (Naive) refers to using randomly sampled values from the true parameter space as the starting points. The errors shown (y-axis) are the differences between the true parameters used to simulate the phylogenies and the values estimated by each method. Each row represents a method, and each column corresponds to the results for one specific parameter. Phylogenies are categorized based on their size into three sectors within each panel, separated by four vertical red dashed lines. From left to right, the sectors are: small phylogenies with fewer than 200 nodes, medium-sized phylogenies with 200 to 500 nodes, and large phylogenies with more than 500 nodes. The values shown in black within each sector are the mean absolute prediction errors of all data points in the sectors. Color coding: The color of the data points illustrates the strength of the carrying capacity effect, calculated as $(\lambda - \mu)/K$. The color gradient transitions from red to purple, indicating increasing strength of the effect. This scale is transformed using log10 for clearer visual differentiation. Small squares spreading along the x-axis signify optimization failures. Extremely deviating estimates are not shown in the figure. X-axis: Size of the phylogenies. Y-axis: Error. λ: Speciation rate. μ: extinction rate. K: carrying capacity.

Table 2. The distribution of relative differences of estimated parameters between using matrix exponentiation ("analytical") and numerical integrator ("odeint::runge_kutta_cash_karp54") with the Simplex optimizer. The "Case" column indicates the maximum-likelihood estimation scenarios, "Best" represents using true speciation rate, true extinction rate and $K = \infty$ as starting points, "Naive" represents using true speciation rate, true extinction rate and $K = 10000$ as starting points. The "Parameter" column indicates each of the estimated parameters, "K" is the carrying capacity, "λ" is the speciation rate and "μ" is the extinction rate. The rest of the columns indicate the ranges of measured relative differences between estimates of the two integration methods. The percentages under these columns indicate the proportions of data having the relative discrepancies smaller than the values indicated by the column names.

**Case**	**Parameter**	**⩽10%**	**⩽1%**	**⩽0.1%**	**⩽0.01%**
Best	K	89.5%	78.6%	73.2%	64.6%
Best	λ	83.9%	78.1%	73.7%	63.2%
Best	μ	83.1%	77.1%	72.6%	61.5%
Naive	K	76.9%	68.9%	64.3%	56.0%
Naive	λ	71.8%	67.3%	64.1%	54.3%
Naive	μ	71.0%	67.0%	62.9%	52.6%

F. Estimation Uncertainty for Empirical Trees

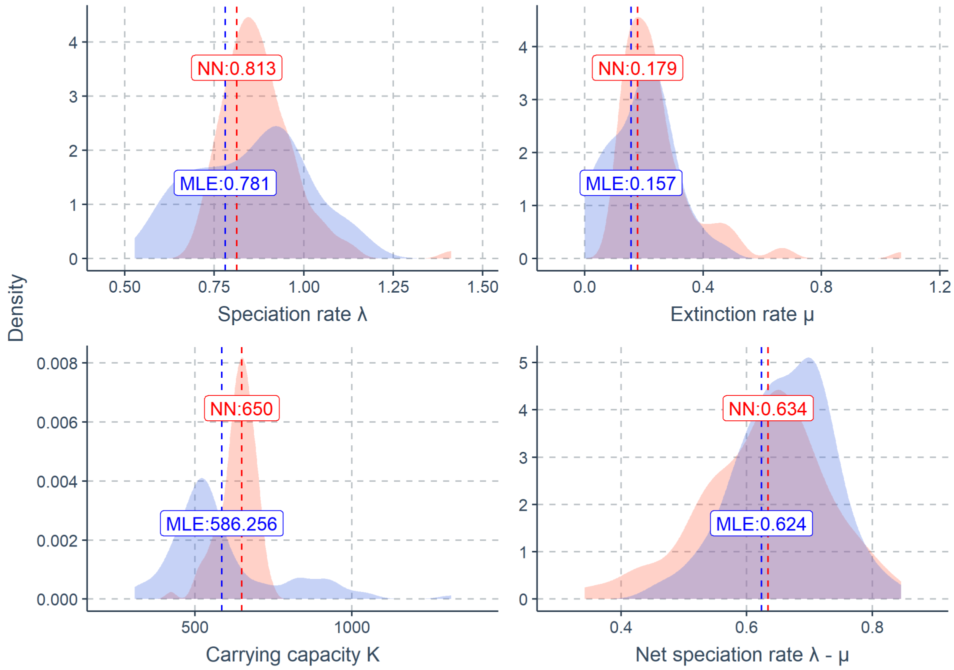

Fig. 15. The neural network estimation uncertainty for a bird phylogeny (Furnariidae). The parameters are estimated using a pre-trained neural network (Boost BT, boosting strategy that corrects GNN results using LSTM) under a diversity-dependent diversification scenario. For reference, maximum likelihood estimation (MLE) is also used to estimate the same parameters. Each panel shows one parameter's estimates using neural network and MLE methods with their uncertainties. The red dashed lines with red numbers indicate the estimates by the neural network method. The blue dashed lines with blue numbers indicate the estimates by the MLE method. Each pink area indicates the density distribution of a neural network estimate from 1000 bootstrap-simulated phylogenies, showing the uncertainty of neural network. Each blue area indicates the density distribution of an MLE estimate from the same set of simulated phylogenies, showing the uncertainty of MLE. X-axis: Parameter (Estimate) values. Y-axis: Density. λ: Speciation rate. μ: Extinction rate. K: Carrying capacity. $\lambda - \mu$: Net speciation rate.

The rest of the figures of neural network estimation uncertainty on the empirical phylogenies under the DDD scenario can be found at `https://github.com/EvoLandEco/eveGNN/tree/master/uncertainty`

G. Results under the Diversity-Dependent Diversification Scenario

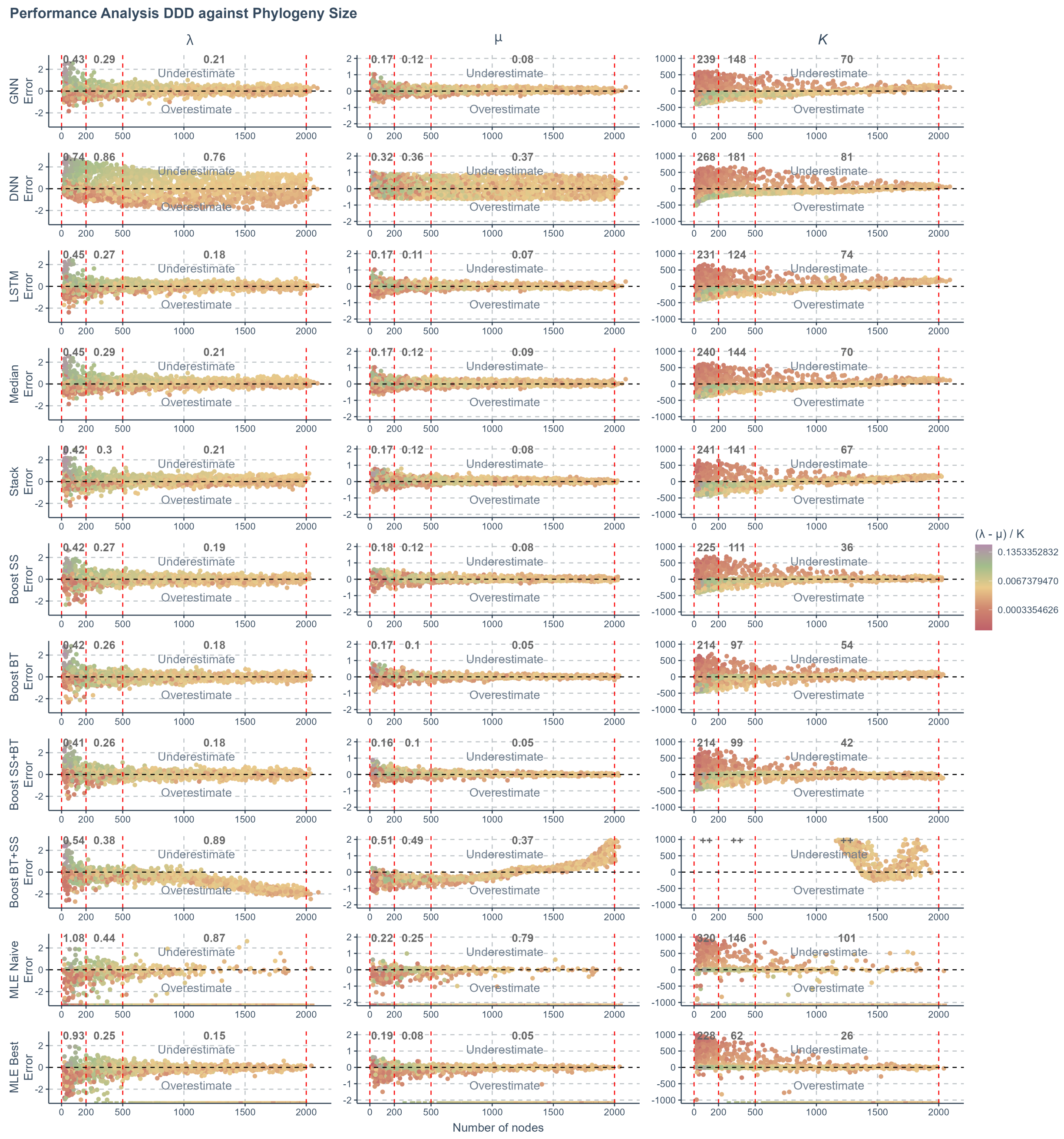

(Caption on next page.)

Fig. 16. *(Figure on previous page.)* The prediction error of various methods applied to phylogenies simulated under a diversity-dependent diversification scenario, against the total number of nodes (including root, internal, and tip nodes) in the phylogenies. The errors shown are the differences between the true parameters used to simulate the phylogenies and the values predicted or estimated by each method. Each row represents a method, and each column corresponds to the results for one specific parameter. Phylogenies are categorized based on their size into three sectors within each panel, separated by four vertical red dashed lines. From left to right, the sectors are: small phylogenies with fewer than 200 nodes, medium-sized phylogenies with 200 to 500 nodes, and large phylogenies with more than 500 nodes. The values shown in black within each sector are the mean absolute prediction errors of all data points in the sectors. Color coding: The color of the data points illustrates the strength of the carrying capacity effect, calculated as $(\lambda - \mu)/K$. The color gradient transitions from red to purple, indicating increasing strength of the effect. This scale is transformed using log10 for clearer visual differentiation. GNN: Predictions obtained by the graph neural network using the phylogenies transformed to graph format. DNN: Predictions by the dense neural network using summary statistics. LSTM: Predictions by the long short-term memory recurrent neural network using branching times. Median: Bagging strategy that takes the median value of the predictions from GNN, DNN, and LSTM. Stack: Stacking strategy that utilizes a meta-learner to integrate results from GNN, DNN, and LSTM. Boost SS: Boosting strategy that corrects GNN results using DNN. Boost BT: Boosting strategy that corrects GNN results using LSTM. Boost SS+BT: Sequential correction of GNN errors first using DNN, followed by LSTM. Boost BT+SS: Sequential correction of GNN errors first using LSTM, followed by DNN. MLE Naive: Maximum Likelihood Estimation results using random starting points for parameter optimization. MLE Best: MLE results using the true parameter values as the starting points for optimization. In the MLE result panels, small squares spreading along the x-axis signify optimization failures. Due to significantly lower accuracy, other aggregation methods from the bagging strategy are not displayed on the plot. X-axis: Size of the phylogenies. Y-axis: Error. λ: Speciation rate. μ: Extinction rate. K: Carrying capacity.

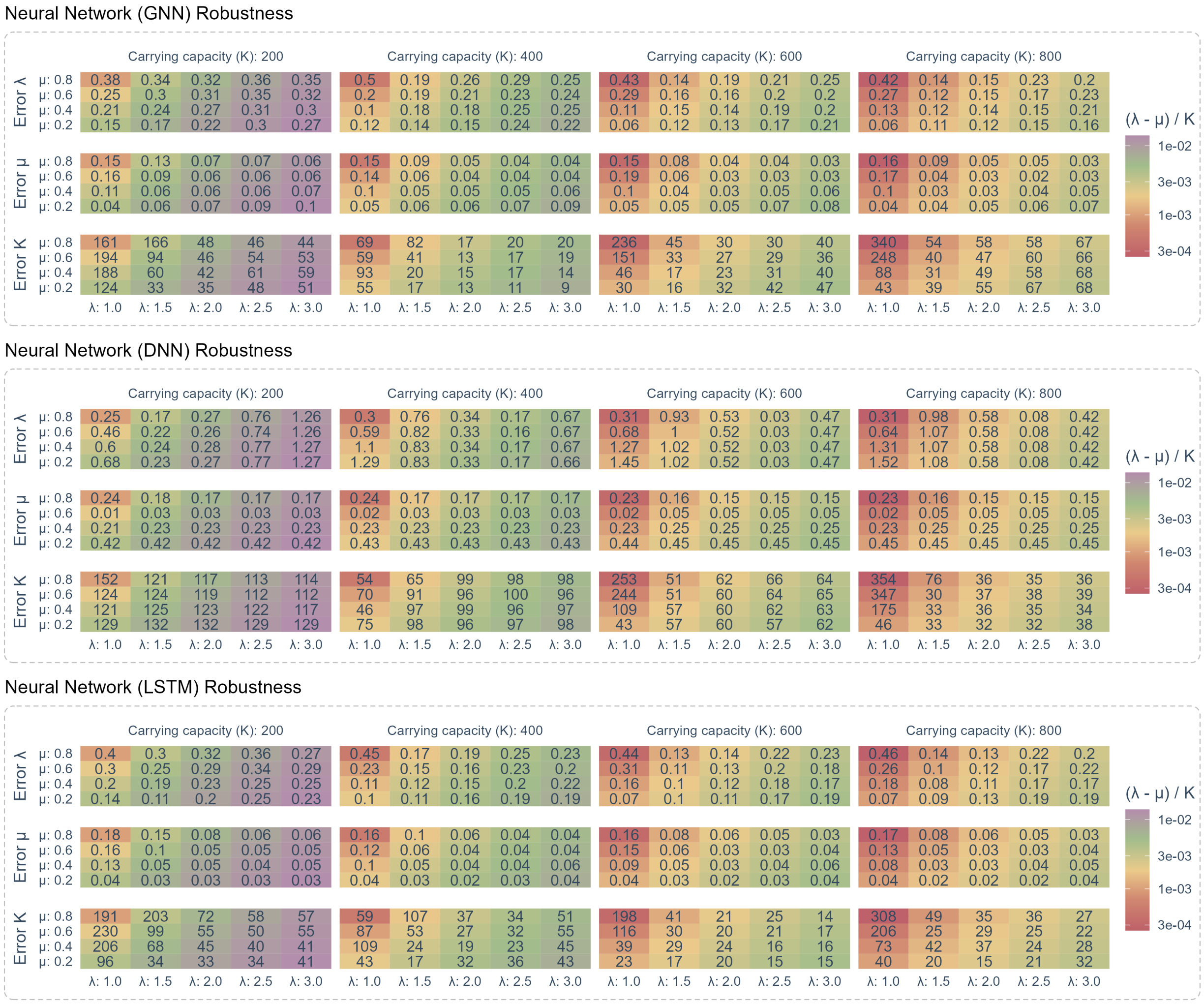

Fig. 17. Robustness comparison between graph neural network (GNN), dense neural network (DNN) and long short-term memory recurrent neural network (LSTM) when operating independently. Same structure as the previous figure.

Neural Network (GNN) Robustness

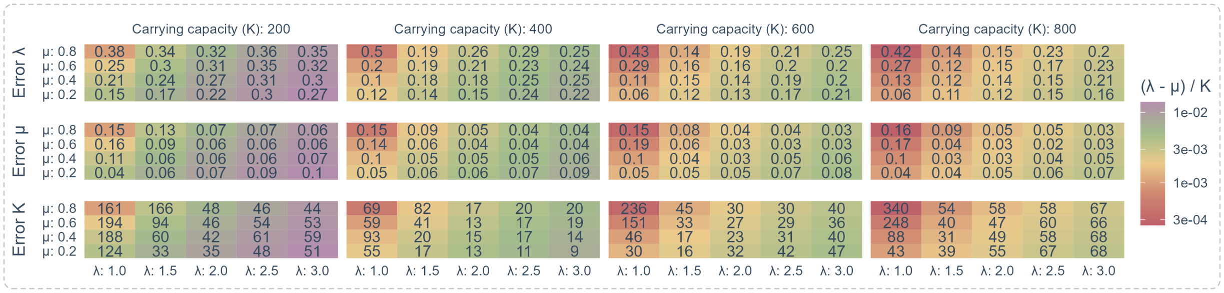

Neural Network (Boosting BT) Robustness

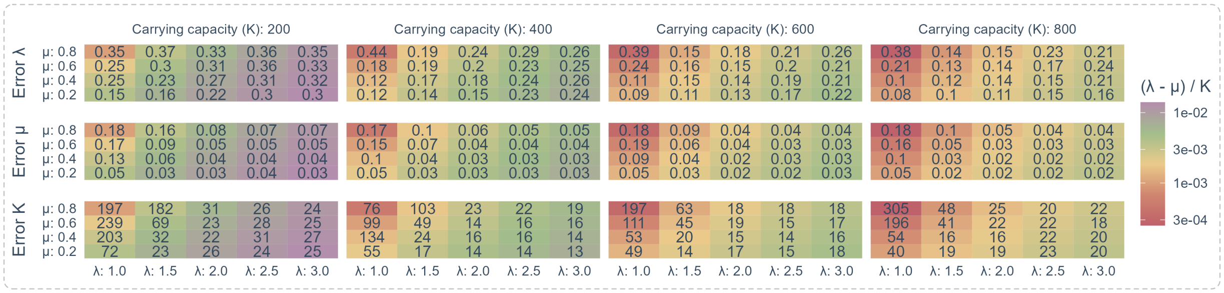

Neural Network (Boosting SS + BT) Robustness

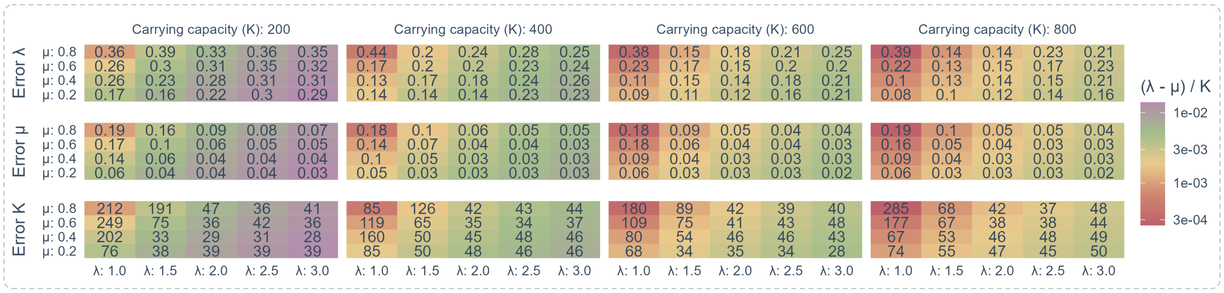

Maximum Likelihood Estimation (Naive Case) Robustness

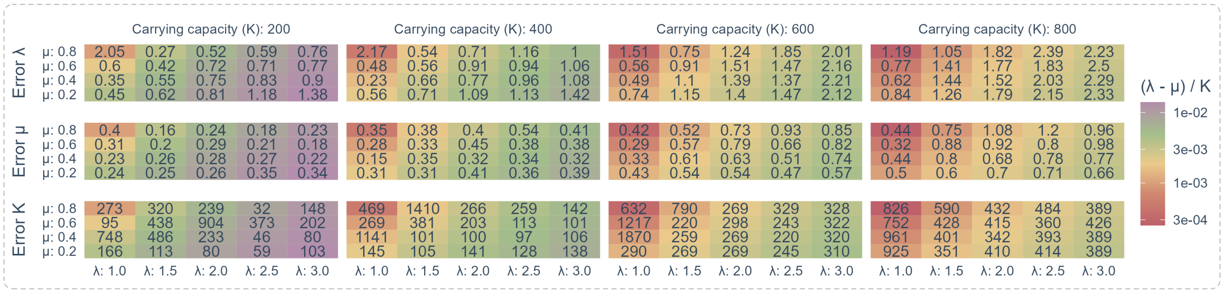

Maximum Likelihood Estimation (Best Case) Robustness

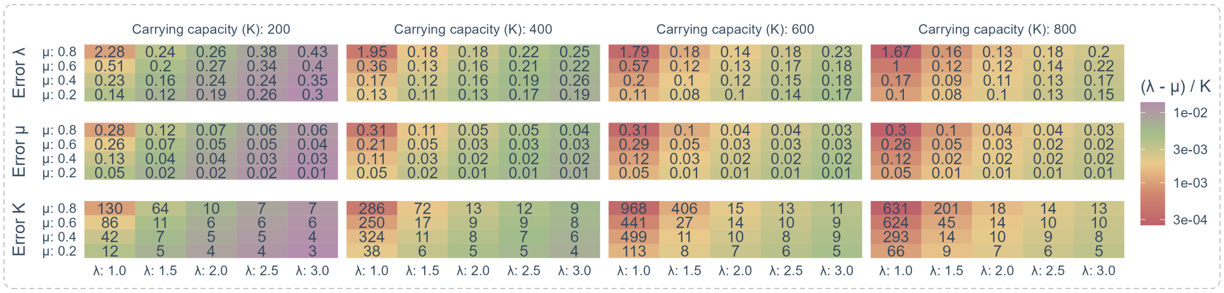

Fig. 18. *(Figure on previous page.)* The robustness of neural network and maximum likelihood estimation was assessed on 80 sets of phylogenies, each containing 1000 trees randomly simulated under a diversity-dependent diversification scenario, employing identical parameter settings but varied in size, topology, and structure. Each segment delineated by dashed lines corresponds to distinct methods. Each column within a segment is associated with a specific carrying capacity (K) used in the simulation of the phylogenies. Each row within a segment details the mean absolute errors between the true and estimated values of a specific parameter, with parameter names labeled on the left side of each row. GNN: Graph neural network. Boosting BT: Graph neural network with long short-term memory recurrent neural network correcting its residuals using branching times. Boosting SS + BT: Graph neural network with dense neural network and long short-term memory recurrent neural network correcting residuals sequentially using summary statistics and branching times. Naive Case: Maximum likelihood estimation using random initial parameter as the starting point. Best Case: Maximum likelihood estimation using true parameter as the starting point. X-axis: Represents the true speciation rate (λ) used to simulate phylogenies. Y-axis: Represents the true extinction rate (μ) used to simulate phylogenies. Cell Content: The numbers displayed within each heatmap cell indicate the mean absolute error for a parameter, given the specific λ, μ and K settings. Color Coding: The background color of each cell illustrates the strength of the carrying capacity effect, calculated as $(\lambda - \mu)/K$. The color gradient transitions from red to purple, indicating increasing strength of the effect. This scale is transformed using log10 for clearer visual differentiation. Note that the numerical values within the cells are not mapped to the background colors. For a detailed reference to the effect strength values corresponding to the background colors, refer to the figure legends.

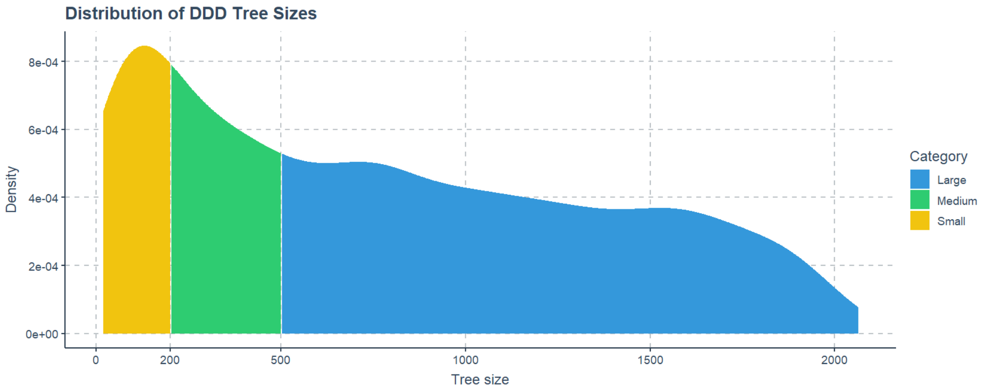

Fig. 19. The density distribution of phylogeny sizes under the diversity-dependent diversification (DDD) scenario. The colors of the areas under the density curve indicate the three categories used in our analyses. Yellow area: Small-sized phylogenies with less than 200 nodes (approx. 100 tips). Greed area: Medium-sized phylogenies with more than 200 nodes and less than 500 nodes (approx. 250 tips). Blue area: Large-sized phylogenies with more than 500 nodes.

H. Results under the Birth-Death Scenario

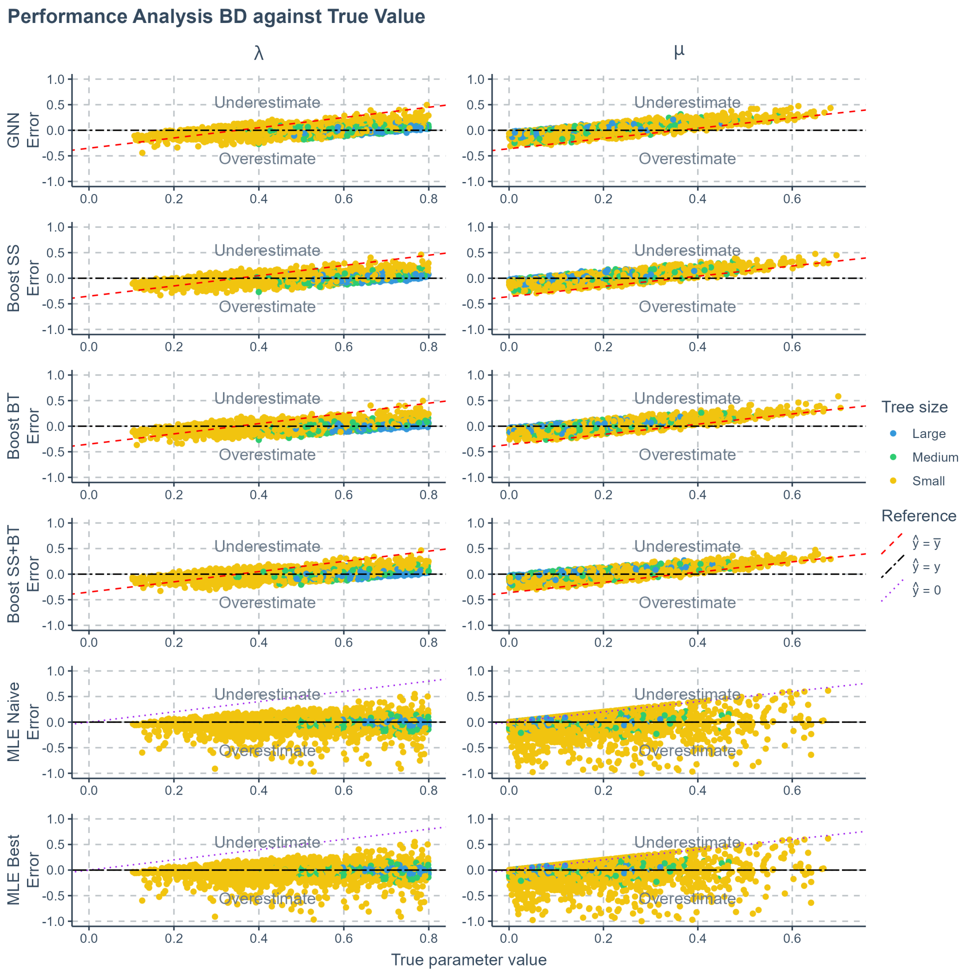

Fig. 20. The prediction error (absolute error) of various methods applied to phylogenies simulated under a birth-death scenario, against true values. The errors shown are the differences between the true parameters used to simulate the phylogenies and the values predicted or estimated by each method. Each row represents a method, and each column corresponds to the results for one specific parameter. Phylogenies are categorized based on their size: yellow for small phylogenies with fewer than 200 nodes (including root, internal, and tip nodes), green for medium-sized phylogenies with 200 to 500 nodes, and blue for large phylogenies with more than 500 nodes. GNN: Predictions obtained by the graph neural network using the phylogenies. Boost SS: Boosting strategy that corrects GNN results using DNN. Boost BT: Boosting strategy that corrects GNN results using LSTM. Boost SS+BT: Sequential correction of GNN errors first using DNN, followed by LSTM. MLE Naive: Maximum Likelihood Estimation results using random starting points for parameter optimization. MLE Best: MLE results using the true parameter values as the starting points for optimization. Red dashed lines in panels representing neural network results indicate the mid-points of the parameter spaces ($\hat{y} = \bar{y}$ where $\hat{y}$ denotes a estimated parameter and $\bar{y}$ denotes the mid-point of the parameter space). Data points close to purple dotted lines ($\hat{y} = 0$) in MLE result panels indicate near-zero estimates. Black two-dash lines indicate accurate estimates ($\hat{y} = y$ where y denotes the true parameter value). X-axis: True parameter values. Y-axis: Error, or difference between true and predicted values. λ: Speciation rate. μ: Extinction rate.

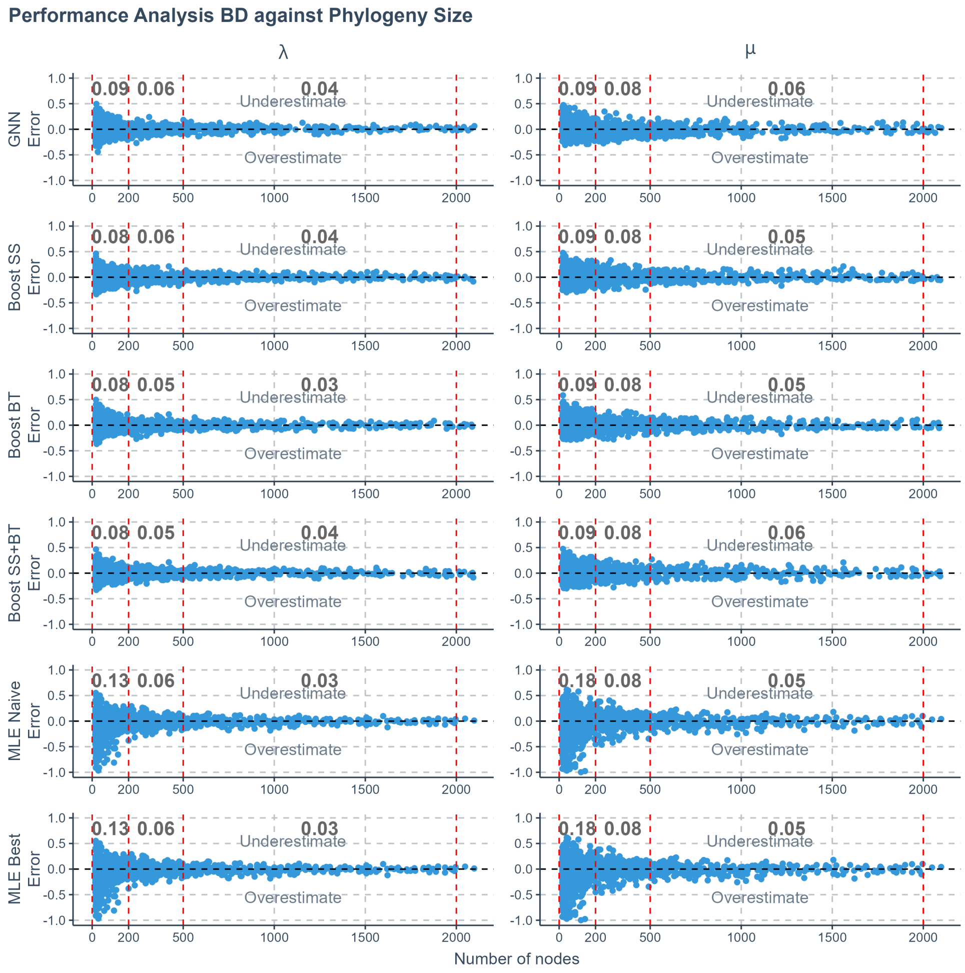

Fig. 21. The prediction error (absolute error) of various methods applied to phylogenies simulated under a birth-death scenario, against the total number of nodes in the phylogenies. The errors shown are the differences between the true parameters used to simulate the phylogenies and the values predicted or estimated by each method. Each row represents a method, and each column corresponds to the results for one specific parameter. Phylogenies are categorized based on their size into three sectors within each panel, separated by four vertical red dashed lines. From left to right, the sectors are: small phylogenies with fewer than 200 nodes (including root, internal, and tip nodes), medium-sized phylogenies with 200 to 500 nodes, and large phylogenies with more than 500 nodes. GNN: Predictions obtained by the graph neural network using the phylogenies. Boost SS: Boosting strategy that corrects GNN results using DNN. Boost BT: Boosting strategy that corrects GNN results using LSTM. Boost SS+BT: Sequential correction of GNN errors first using DNN, followed by LSTM. MLE Naive: Maximum Likelihood Estimation results using random starting points for parameter optimization. MLE Best: MLE results using the true parameter values as the starting points for optimization. X-axis: Size of the phylogenies. Y-axis: Error. λ: Speciation rate. μ: Extinction rate.

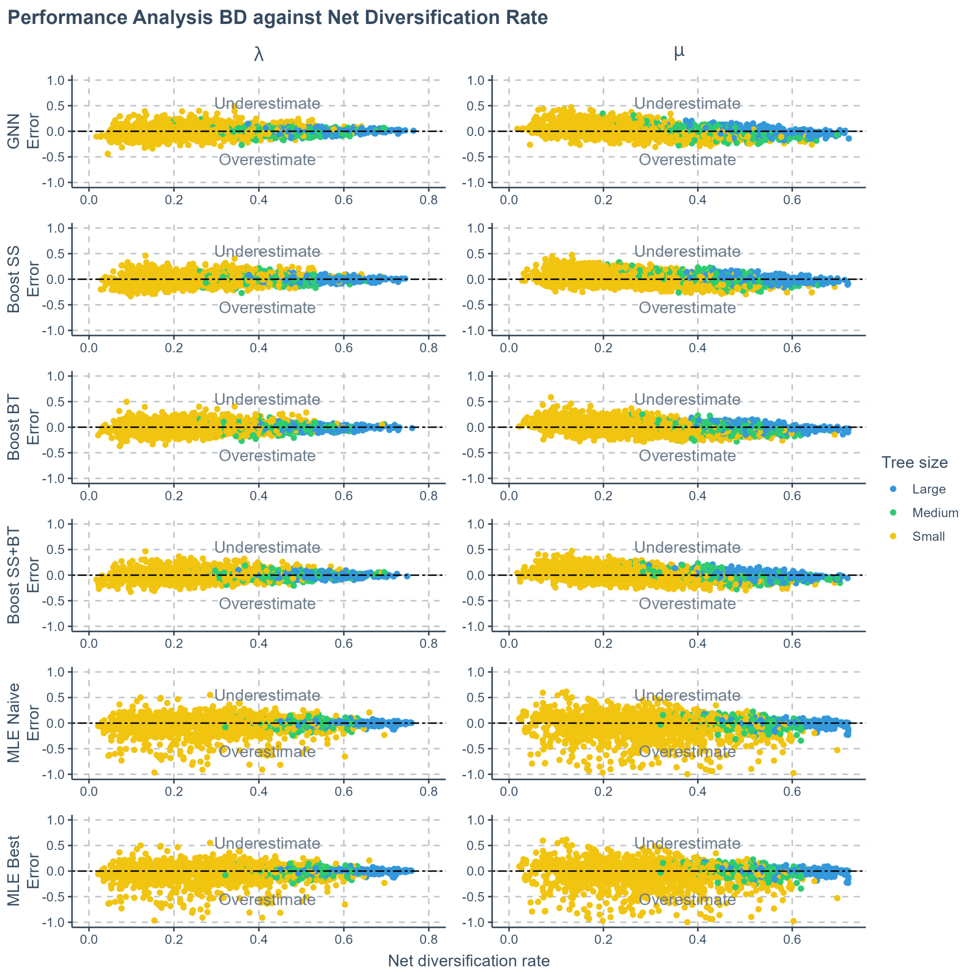

Fig. 22. The prediction error (absolute error) of various methods applied to phylogenies simulated under a birth-death scenario, against net diversification rate ($\lambda - \mu$). The errors shown are the differences between the true parameters used to simulate the phylogenies and the values predicted or estimated by each method. Each row represents a method, and each column corresponds to the results for one specific parameter. Phylogenies are categorized based on their size: yellow for small phylogenies with fewer than 200 nodes (including root, internal, and tip nodes), green for medium-sized phylogenies with 200 to 500 nodes, and blue for large phylogenies with more than 500 nodes. GNN: Predictions obtained by the graph neural network using the phylogenies. Boost SS: Boosting strategy that corrects GNN results using DNN. Boost BT: Boosting strategy that corrects GNN results using LSTM. Boost SS+BT: Sequential correction of GNN errors first using DNN, followed by LSTM. MLE Naive: Maximum Likelihood Estimation results using random starting points for parameter optimization. MLE Best: MLE results using the true parameter values as the starting points for optimization. Black two-dash lines indicate accurate estimate. X-axis: True parameter values. Y-axis: Error, or difference between true and predicted values. λ: Speciation rate. μ: Extinction rate.

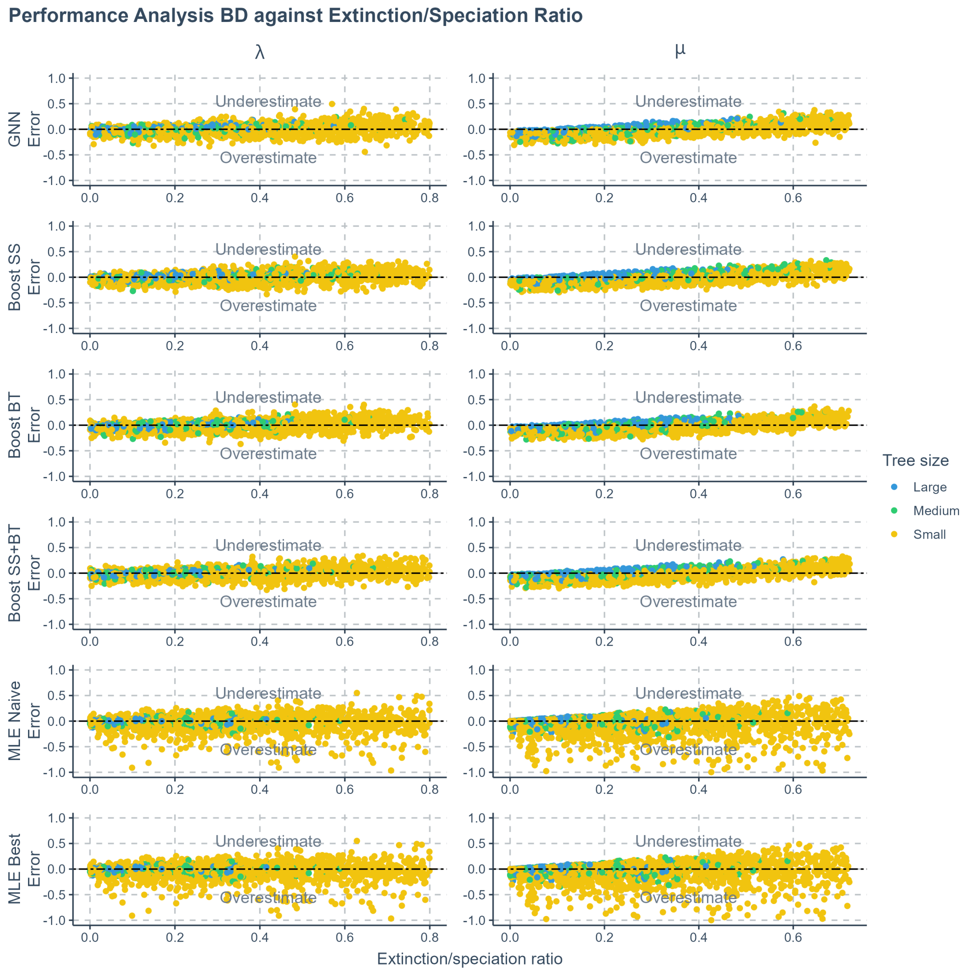

Fig. 23. The prediction error (absolute error) of various methods applied to phylogenies simulated under a birth-death scenario, against extinction/speciation ratio (μ/λ). The errors shown are the differences between the true parameters used to simulate the phylogenies and the values predicted or estimated by each method. Each row represents a method, and each column corresponds to the results for one specific parameter. Phylogenies are categorized based on their size: yellow for small phylogenies with fewer than 200 nodes (including root, internal, and tip nodes), green for medium-sized phylogenies with 200 to 500 nodes, and blue for large phylogenies with more than 500 nodes. GNN: Predictions obtained by the graph neural network using the phylogenies. Boost SS: Boosting strategy that corrects GNN results using DNN. Boost BT: Boosting strategy that corrects GNN results using LSTM. Boost SS+BT: Sequential correction of GNN errors first using DNN, followed by LSTM. MLE Naive: Maximum Likelihood Estimation results using random starting points for parameter optimization. MLE Best: MLE results using the true parameter values as the starting points for optimization. Black two-dash lines indicate accurate estimates. X-axis: True parameter values. Y-axis: Error, or difference between true and predicted values. λ: Speciation rate. μ: Extinction rate.

I. Results under the Protracted Birth-Death Scenario

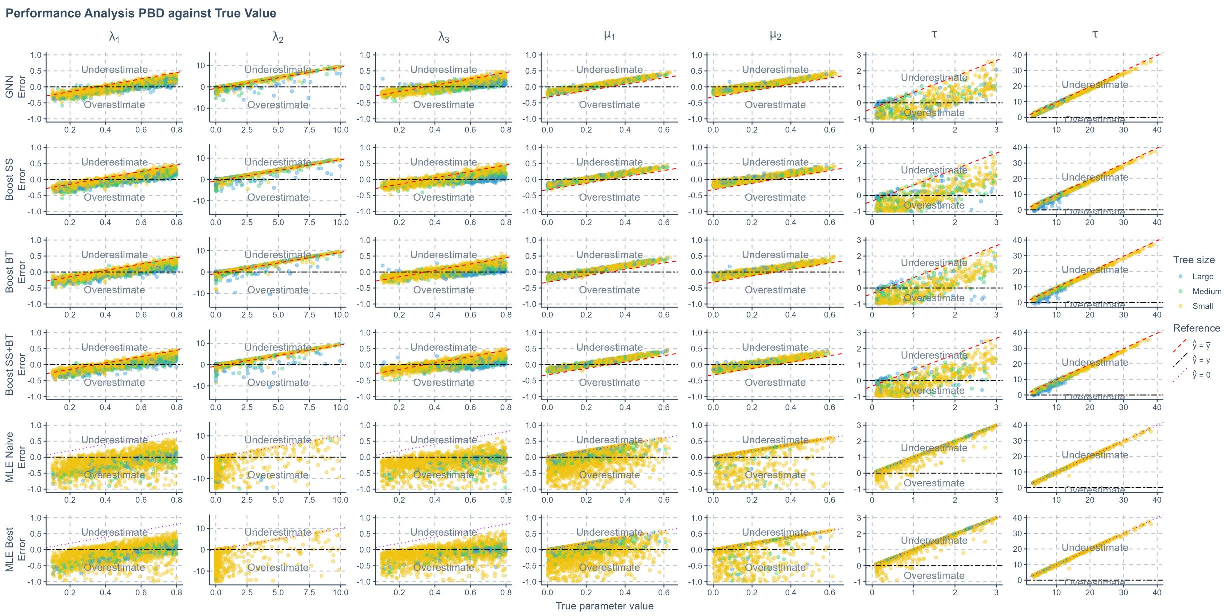

Fig. 24. The prediction error (absolute error) of various methods applied to phylogenies simulated under a protracted birth-death scenario, against true values. The errors shown are the differences between the true parameters used to simulate the phylogenies and the values predicted or estimated by each method. Each row represents a method, and each column corresponds to the results for one specific parameter. Phylogenies are categorized based on their size: yellow for small phylogenies with fewer than 200 nodes (including root, internal, and tip nodes), green for medium-sized phylogenies with 200 to 500 nodes, and blue for large phylogenies with more than 500 nodes. GNN: Predictions obtained by the graph neural network using the phylogenies. Boost SS: Boosting strategy that corrects GNN results using DNN. Boost BT: Boosting strategy that corrects GNN results using LSTM. Boost SS+BT: Sequential correction of GNN errors first using DNN, followed by LSTM. MLE Naive: Maximum Likelihood Estimation results using random starting points for parameter optimization. MLE Best: MLE results using the true parameter values as the starting points for optimization. Red dashed lines in panels representing neural network results indicate the mid-points of the parameter spaces. Purple dotted lines in MLE result panels signify where estimated values are 0. X-axis: True parameter values. Y-axis: Error, or difference between true and predicted values. λ_1: Speciation initiation rate of the good species. λ_2: Speciation completion rate. λ_3: Speciation initiation rate of the incipient species. μ_1: Extinction rate of the good species. μ_2: Extinction rate of the incipient species. τ: Expected duration of speciation.

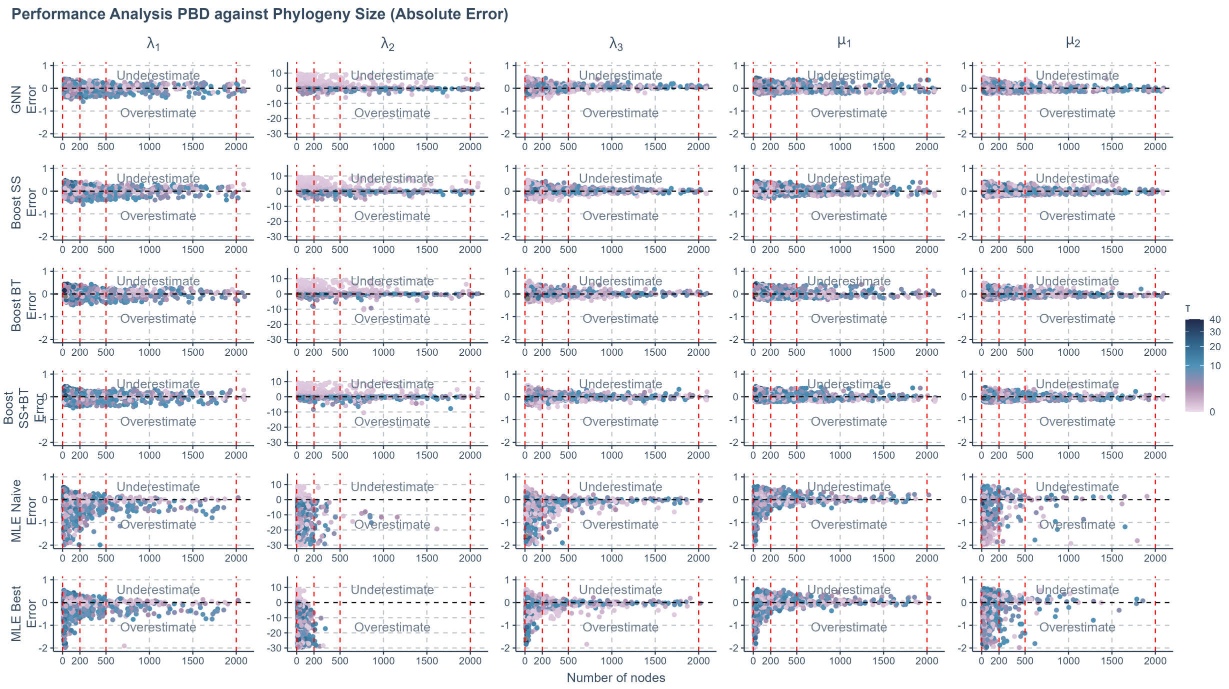

Fig. 25. The prediction error (absolute error) of various methods applied to phylogenies simulated under a protracted birth-death scenario, against the total number of nodes in the phylogenies. The errors shown are the differences between the true parameters used to simulate the phylogenies and the values predicted or estimated by each method. Each row represents a method, and each column corresponds to the results for one specific parameter. Phylogenies are categorized based on their size into three sectors within each panel, separated by four vertical red dashed lines. From left to right, the sectors are: small phylogenies with fewer than 200 nodes (including root, internal, and tip nodes), medium-sized phylogenies with 200 to 500 nodes, and large phylogenies with more than 500 nodes. Color Coding: The color of the data points illustrates the expected duration of speciation. The color gradient transitions from light purple to dark blue, indicating increasing value of the duration. This scale is transformed using square root for clearer visual differentiation. GNN: Predictions obtained by the graph neural network using the phylogenies. Boost SS: Boosting strategy that corrects GNN results using DNN. Boost BT: Boosting strategy that corrects GNN results using LSTM. Boost SS+BT: Sequential correction of GNN errors first using DNN, followed by LSTM. MLE Naive: Maximum Likelihood Estimation results using random starting points for parameter optimization. MLE Best: MLE results using the true parameter values as the starting points for optimization. X-axis: Size of the phylogenies. Y-axis: Error. λ_1: Speciation initiation rate of the good species. λ_2: Speciation completion rate. λ_3: Speciation initiation rate of the incipient species. μ_1: Extinction rate of the good species. μ_2: Extinction rate of the incipient species. τ: Expected duration of speciation.

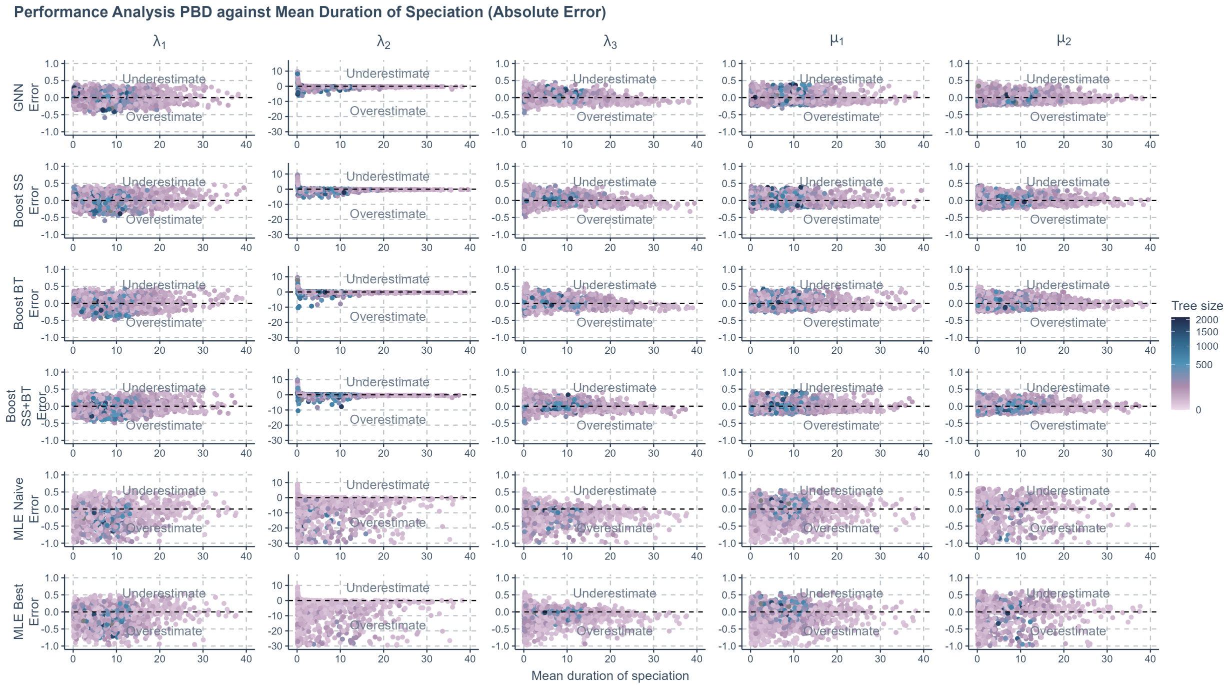

Fig. 26. The prediction error (absolute error) of various methods applied to phylogenies simulated under a protracted birth-death scenario, against the true mean duration of speciation. The errors shown are the differences between the true parameters used to simulate the phylogenies and the values predicted or estimated by each method. Each row represents a method, and each column corresponds to the results for one specific parameter. Color Coding: The color of the data points illustrates the total number of nodes of the phylogenies. The color gradient transitions from light purple to dark blue, indicating increasing value of the node number. This scale is transformed using square root for clearer visual differentiation. GNN: Predictions obtained by the graph neural network using the phylogenies. Boost SS: Boosting strategy that corrects GNN results using DNN. Boost BT: Boosting strategy that corrects GNN results using LSTM. Boost SS+BT: Sequential correction of GNN errors first using DNN, followed by LSTM. MLE Naive: Maximum Likelihood Estimation results using random starting points for parameter optimization. MLE Best: MLE results using the true parameter values as the starting points for optimization. X-axis: Size of the phylogenies. Y-axis: Error. λ_1: Speciation initiation rate of the good species. λ_2: Speciation completion rate. λ_3: Speciation initiation rate of the incipient species. μ_1: Extinction rate of the good species. μ_2: Extinction rate of the incipient species. τ: Expected duration of speciation.

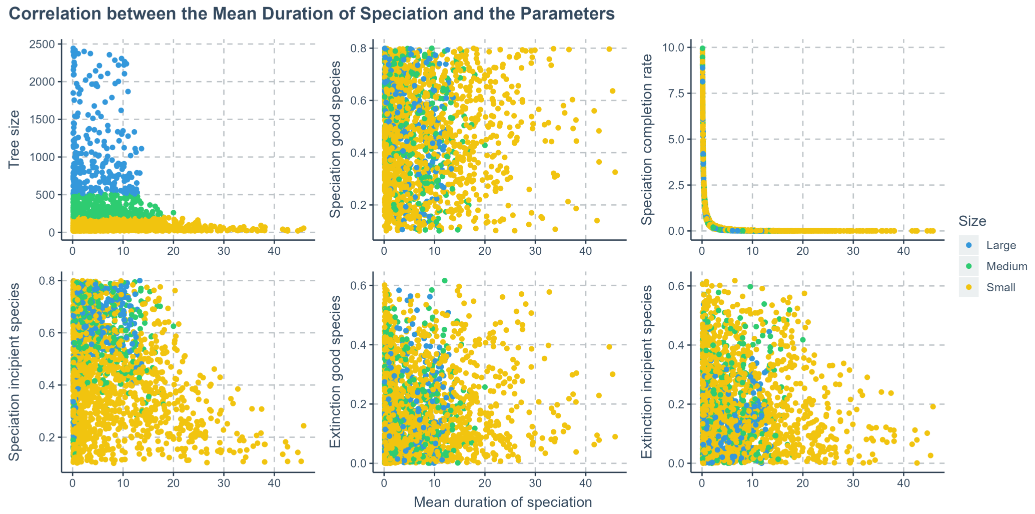

Fig. 27. The correlation between the mean duration of speciation and the true parameter values under the protracted birth-death diversification scenario. Phylogenies are categorized based on their size: yellow for small phylogenies with fewer than 200 nodes (including root, internal, and tip nodes), green for medium-sized phylogenies with 200 to 500 nodes, and blue for large phylogenies with more than 500 nodes. X-axis: Mean duration of speciation. Y-axis: Tree size.

J. Comparison between Our Methods and Existing Methods

To benchmark our approaches against a convolutional architecture, we replaced the Median bagging panel in all performance figures related to the DDD scenario with a CNN1D panel reflecting the implementation of Voznica et al. (2022). This allows a direct sidebyside comparison between our neural networks and one of the bestperforming 1DCNNs in the literature.

Overall, the architecture by Voznica et al. (2022) performed similarly and exhibited similar patterns as our approaches. CNN1D better recovered the carrying capacity effect strength than than GNN, DNN and LSTM alone, but lagged behind our boosting approaches–except for Boost BT+SS–in overall parameter prediction accuracy.

See the figures below for details.

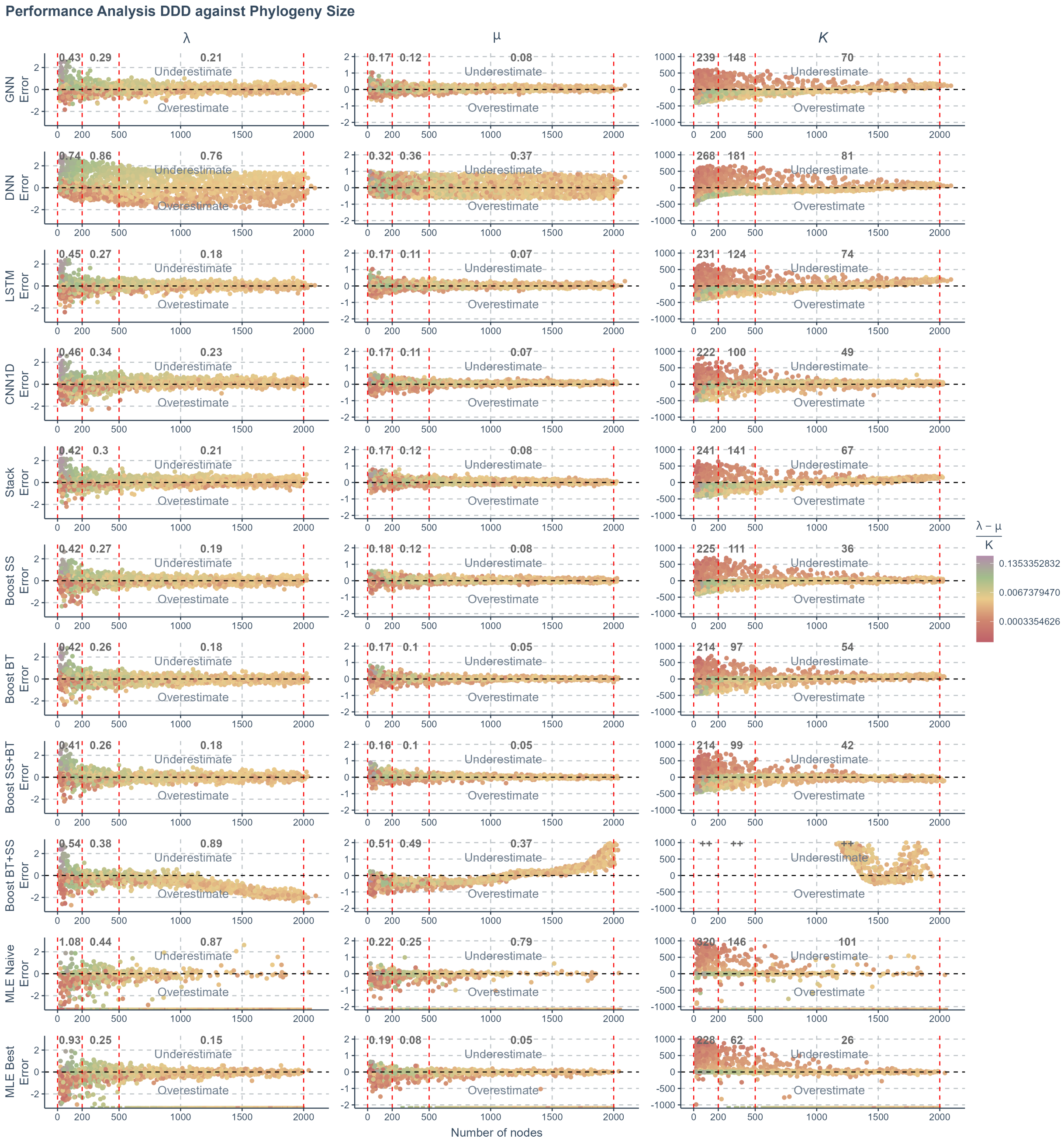

Fig. 28. Prediction error of estimated parameters on trees of different sizes under a diversity-dependent diversification scenario. Compared to the original figure in the results section, the "Median" bagging approach panel is replaced with "CNN1D" established by Voznica et al. (2022).

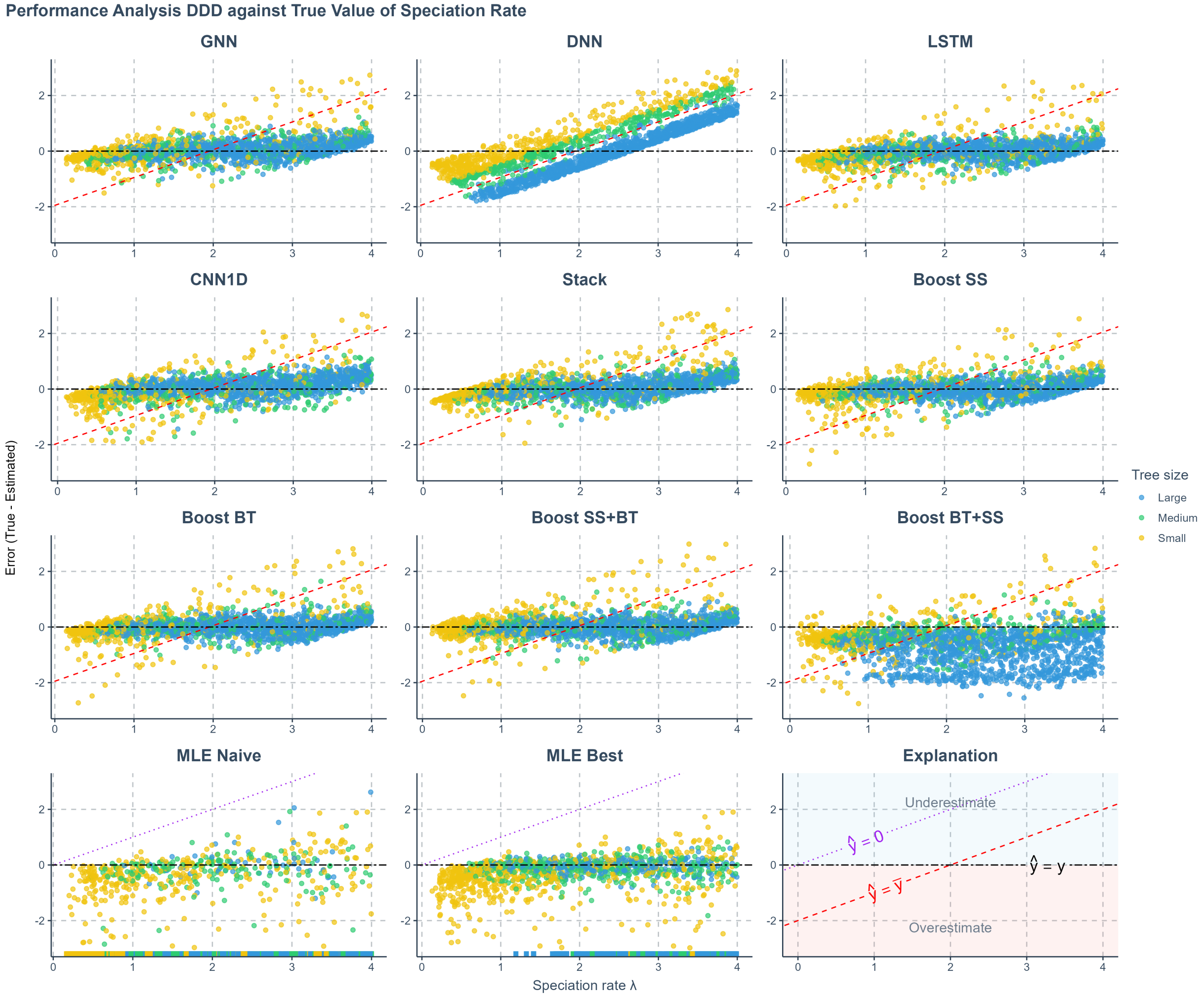

Fig. 29. Prediction error of speciation rate plotted against true speciation rate under a diversity-dependent diversification scenario. Compared to the original figure in the results section, the "Median" bagging approach panel is replaced with "CNN1D" established by Voznica et al. (2022).

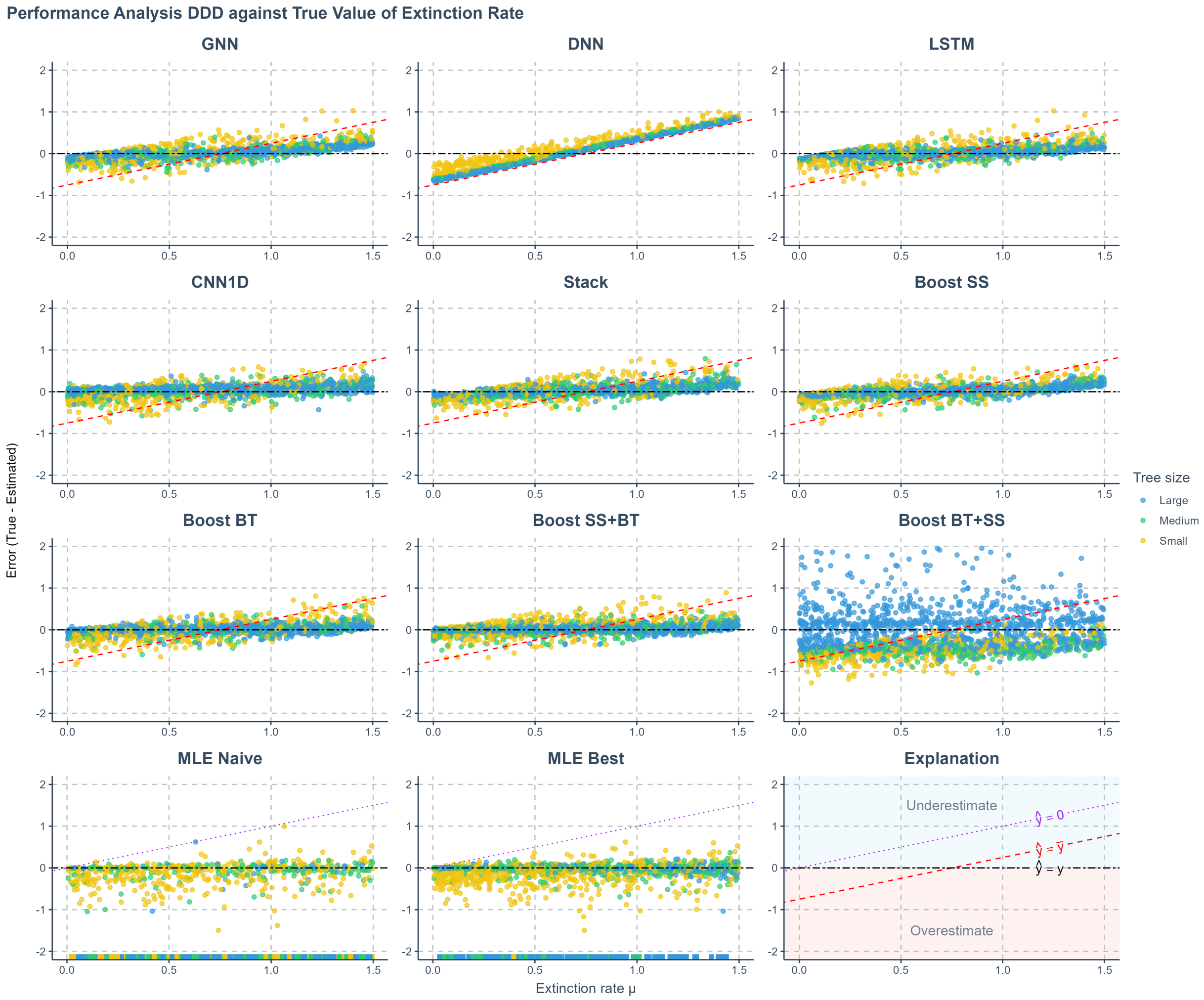

Fig. 30. Prediction error of extinction rate plotted against true extinction rate under a diversity-dependent diversification scenario. Compared to the original figure in the results section, the "Median" bagging approach panel is replaced with "CNN1D" established by Voznica et al. (2022).

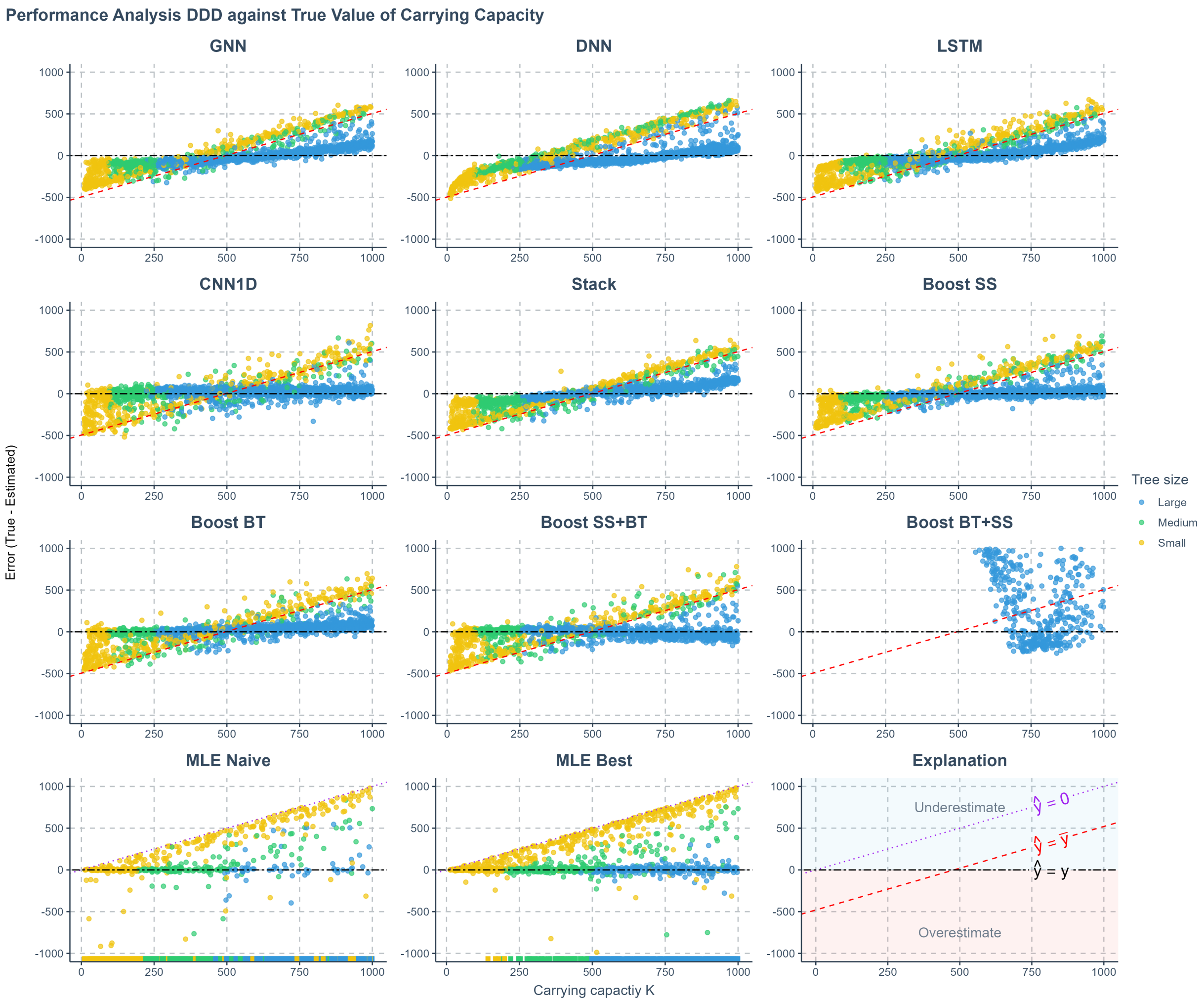

Fig. 31. Prediction error of carrying capacity plotted against true carrying capacity under a diversity-dependent diversification scenario. Compared to the original figure in the results section, the "Median" bagging approach panel is replaced with "CNN1D" established by Voznica et al. (2022).

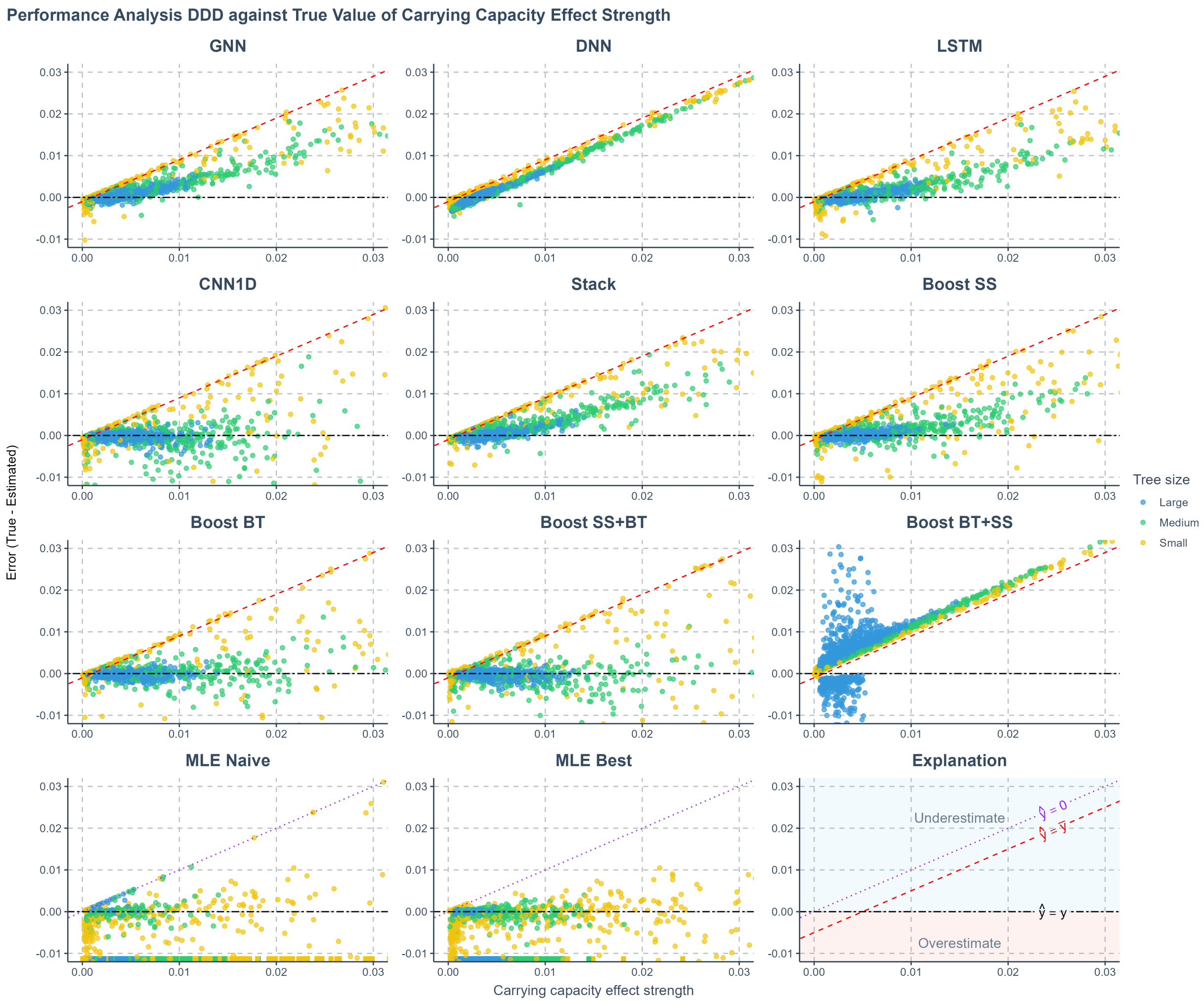

Fig. 32. Prediction error of effect strength of carrying capacity plotted against true effect strength of carrying capacity under a diversity-dependent diversification scenario. Compared to the original figure in the results section, the "Median" bagging approach panel is replaced with "CNN1D" established by Voznica et al. (2022).

K. Dataset Re-balancing

Birth-death processes without carrying capacity effect may have larger variance of tree size than the DDD trees. A skew in the frequency of tree size across datasets may appear that leads to a non-representative sample.

To address this issue, we re-balanced the BD dataset by creating 10 bins, each designated to hold phylogenies within specific size ranges, spanning from 10 to 2000 nodes in increments of 200 nodes per bin (the first bin accepts phylogeny of sizes 10 to 200). We randomly simulated phylogenies using parameters sampled from the same space as the BD training dataset and allocated them to these bins according to their sizes, continuing this process until each bin reached its target capacity of 10,000 phylogenies. This method leads to a more equal representation of phylogenies of each size range, reducing size-based sampling bias. The filled bins were subsequently combined to form a re-balanced dataset, which in total has 100,000 phylogenies.

To compare with the original BD dataset, we trained neural networks on the re-balanced dataset, and validated neural network performance on an additional testing dataset (10,000 phylogenies simulated using the same parameter space). We computed MLE estimates on 2,000 randomly sampled phylogenies from the testing dataset. See Figure 33 for the results.

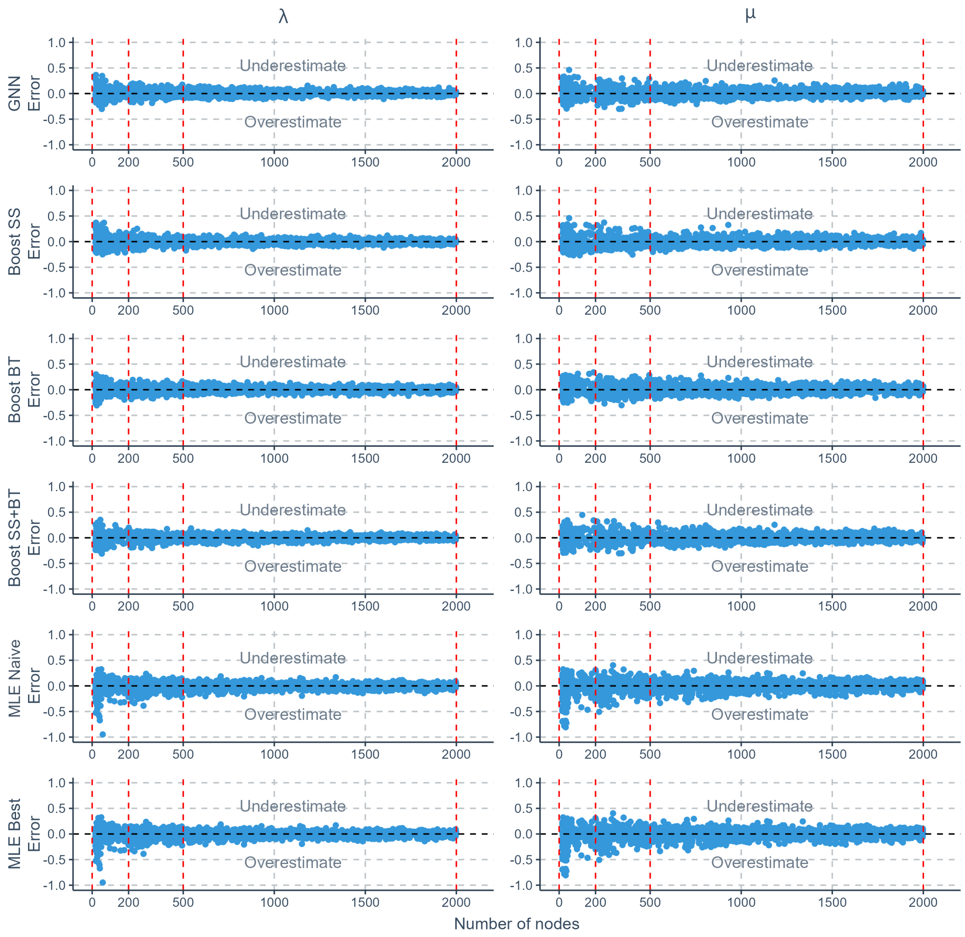

Fig. 33. The prediction error (absolute error) of various methods applied to re-balanced phylogenies simulated under a birth-death scenario, against the total number of nodes in the phylogenies. The errors shown are the differences between the true parameters used to simulate the phylogenies and the values predicted or estimated by each method. Each row represents a method, and each column corresponds to the results for one specific parameter. Phylogenies are categorized based on their size into three sectors within each panel, separated by four vertical red dashed lines. From left to right, the sectors are: small phylogenies with fewer than 200 nodes (including root, internal, and tip nodes), medium-sized phylogenies with 200 to 500 nodes, and large phylogenies with more than 500 nodes. This scale is transformed using square root for clearer visual differentiation. GNN: Predictions obtained by the graph neural network using the phylogenies. Boost SS: Boosting strategy that corrects GNN results using DNN. Boost BT: Boosting strategy that corrects GNN results using LSTM. Boost SS+BT: Sequential correction of GNN errors first using DNN, followed by LSTM. MLE Naive: Maximum Likelihood Estimation results using random starting points for parameter optimization. MLE Best: MLE results using the true parameter values as the starting points for optimization. X-axis: Size of the phylogenies. Y-axis: Error. λ: Speciation rate. μ: Extinction rate.

L. Data outside the Training Space and Complete Phylogeny

We simulated additional datasets to explore the generalization ability of the neural networks when facing data with true parameters completely outside the training space, as well as to compare neural network performances between extant and complete phylogenies. Each simulated dataset was divided into in-distribution and out-of-distribution datasets. We used the in-distribution datasets for training and testing and the out-of-distribution datasets for evaluating the generalization ability of the trained neural networks. Validating trained neural networks on the out-of-distribution datasets can provide insights into whether their performances are tailored to the peculiarities of the already seen data and whether they are robust to new, unseen phylogenies. For each tree we kept two versions: tree of all species (TAS) and tree of extant species (TES). See Table 3 for the parameter settings of the additional datasets, see Table 4 for the criteria of in-distribution and out-of-distribution dataset separation. To conserve GPU memory, the parameter space for additional datasets was deliberately kept smaller, given that the TAS dataset inherently contains far more information than the TES.

A: Parameter settings for BD and DDD trees

Type	Age	N	λ_0		μ_0		K	
			a	b	a	b	a	b
BD	10	60k	0.1	0.6	0.0	$0.9\lambda_0$	-	-
DDD	10	100k	0.1	3.0	0.0	$0.9\lambda_0{}^*$	10	1000

B: Parameter settings for PBD trees

Type	Age	N	b_1		λ_1		b_2		μ_1		μ_2	
			a	b	a	b	a	b	a	b	a	b
PBD	10	100k	0.1	0.8	0.001	10	0.1	0.8	0.0	$0.8b_1$	0.0	$0.8b_2$

Table 3. List of simulated tree datasets. The type column specifies which function is used to generate the trees. The age column specifies the crown age of the trees. The N column specifies the number of trees in the dataset. The rest of the columns specify the lower (a) and the upper (b) bounds of the initial parameters for the tree simulations, all the parameters are sampled from $U(a, b)$ except for λ_1 of the protracted birth-death scenario. λ_1 is computed as $\lambda_1 = 10^e$ where e is sampled from $U(-3, 1)$. U denotes uniform distribution. List A shows the parameter distributions of the birth-death trees and the diversity-dependent-diversification trees, λ: intrinsic speciation rate/birth rate; μ: intrinsic extinction rate/death rate; K: carrying capacity. List B shows the parameter distributions of the protracted birth-death trees, λ_1: speciation-initiation rate of good species; λ_2: speciation-completion rate; λ_3: speciation-initiation rate of incipient species; μ_1: extinction rate of good species; μ_2: extinction rate of incipient species. *In diversity-dependent-diversification simulations, the maximum extinction rate is capped at 1.5 if $0.9\lambda > 1.5$.

Model	Parameter	Left Out	In	Right Out
BD	λ_0	$[0.10, 0.18)$	$[0.18, 0.52]$	$(0.52, 0.60]$
BD	μ_0	$[0.00, 0.08)$	$[0.08, 0.46]$	$(0.46, 0.54]$
DDD	λ_0	$[0.00, 0.30)$	$[0.30, 2.70]$	$(2.70, 3.00]$
DDD	μ_0	$[0.00, 0.10)$	$[0.10, 0.80]$	$(0.80, 0.90]$
DDD	K	$[10, 100)$	$[100, 900]$	$(900, 1000]$
PBD	b_1	$[0.10, 0.18)$	$[0.18, 0.72]$	$(0.72, 0.80]$
PBD	λ_1	$[0.001, 0.002)$	$[0.002, 5]$	$(5, 10]$
PBD	b_2	$[0.10, 0.18)$	$[0.18, 0.72]$	$(0.72, 0.80]$
PBD	μ_1	$[0.00, 0.06)$	$[0.06, 0.58]$	$(0.58, 0.64]$
PBD	μ_2	$[0.00, 0.06)$	$[0.06, 0.58]$	$(0.58, 0.64]$

Table 4. Criteria for in-distribution (in-sample) and out-of-distribution (out-of-sample) dataset separation. Trees generated from each model are separated into left out-of-sample group, in-sample group and right out-of-sample group, based on the parameter ranges. The Model column shows the model of a parameter; the Parameter column shows the corresponding parameter; the Left Out column shows the criteria for the left out-of-sample group; the In Sample column shows the criteria for the in-sample group; the Right Out column shows the criteria of the right out-of-sample group. λ: intrinsic speciation rate/birth rate; μ: intrinsic extinction rate/death rate; K: carrying capacity. List B shows the parameter distributions of the protracted birth-death trees, λ_1: speciation-initiation rate of good species; λ_2: speciation-completion rate; λ_3: speciation-initiation rate of incipient species; μ_1: extinction rate of good species; μ_2: extinction rate of incipient species.

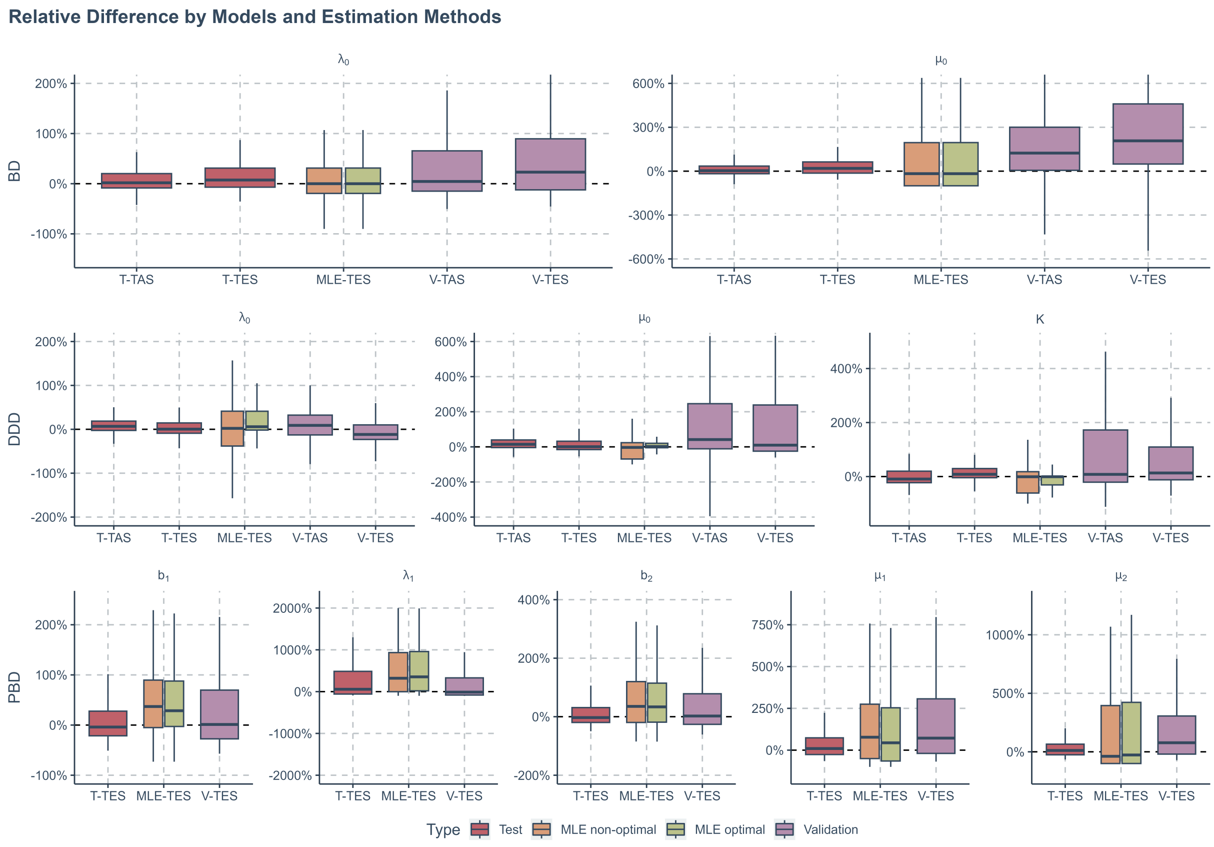

Fig. 34. Comparisons of relative differences (in percentage) between ground true and estimated parameter values. From top to bottom, the panels in each row present the relative differences of trees generated by a specific diversification process. From left to right, the panels in each column present the relative differences of a specific parameter used when simulating the trees. Within each panel, each box represents a specific method for parameter estimation on a specific data set (as described in x-axis labels). Red boxes represent parameter estimation by using only graph neural network (GNN) on the in-sample datasets (Test in figure), yellow boxes represent the non-optimal maximum likelihood estimation (MLE) method on the complete datasets (direct outputs from simulation, without any separation), green boxes represent the optimal MLE method on the complete datasets, purple boxes represent parameter estimation by GNN on the out-of-sample datasets. BD - birth-death trees; DDD - diversity-dependent-diversification trees; PBD - protracted birth-death trees. λ - birth rate/intrinsic speciation rate; μ death rate/intrinsic extinction rate; K - carrying capacity; λ_1 - speciation rate of good species; λ_2 - speciation-completion rate; λ_3 - speciation rate of incipient species; μ_1 - extinction rate of good species; μ_2 extinction rate of incipient species. T-TAS - GNN parameter estimation on full trees (with extinct lineages) in the in-sample data set; T-TES - GNN parameter estimation on extant trees (without extinct lineages) in the in-sample dataset; MLE-TES - MLE parameter estimation on extant trees in the complete dataset; V-TAS - GNN parameter estimation on full trees in the out-of-sample dataset; V-TES - GNN parameter estimation on extant trees in the out-of-sample dataset.

M. Learning from Summary Statistics

To analyze the relationships between summary statistics and the true parameters used in the diversity-dependent diversification simulations, we computed Pearson correlation indices for each summary statistic against the true values of the three parameters. The absolute values of the correlations are visualized as a heatmap, detailed in Figure 35.

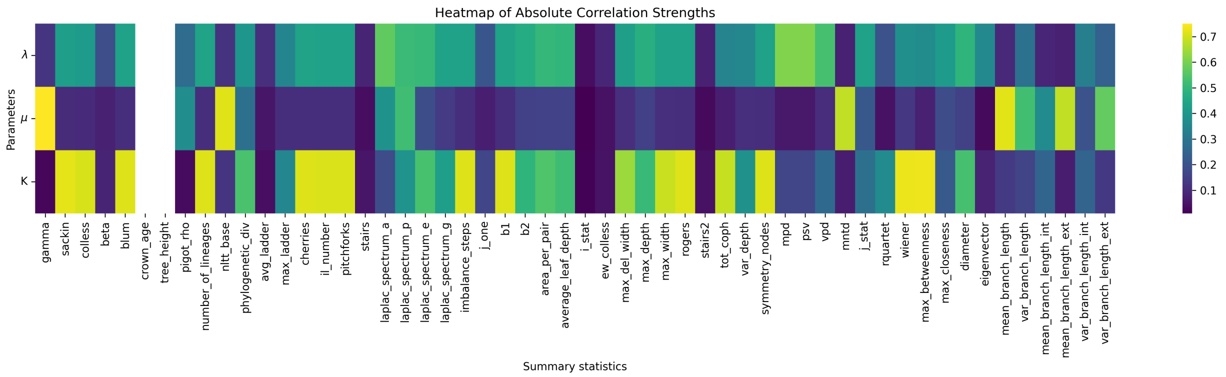

Fig. 35. Heatmap of absolute correlation strengths between true parameters and summary statistics from simulated trees under a diversity-dependent diversification scenario. Each column corresponds to a specific summary statistic, while each row corresponds to a true parameter that was used to simulate the phylogenies. The true parameters are denoted as follows: λ for speciation rate, μ for extinction rate, and K for carrying capacity. The color gradient, ranging from dark purple to yellow, represents the increasing values of the absolute Pearson correlations between the summary statistics and the true parameters, See Appendix O for the details of the statistics.

The heatmap analysis reveals that a large number of summary statistics showed a high correlation with carrying capacity, whereas strong correlations with speciation and extinction rates are considerably less frequent. Generally, the correlation strength between the true parameters and extinction rate is markedly lower. These findings align with the observed performance of DNN, which generally yields good estimates for carrying capacity but is much less effective in accurately predicting speciation rates and particularly poor at estimating extinction rates.

We further evaluated the DNN's performance by benchmarking it against a range of non-neural-network regression techniques using the same summary statistics. These

included multivariate linear regression, Ridge regression, Lasso regression, random forest regression, and gradient boosting regression. Our findings indicated that while these traditional regression and machine learning methods generally outperformed the DNN, they still did not match the performance of our more complex neural networks.

Traditional regression methods and other machine learning techniques often outperform linear feed-forward neural networks in classical regression tasks. Such methods can stabilize performance with less data compared to neural networks, which usually require large datasets to generalize effectively. In our study, the dataset size – consisting of 100,000 entries across 54 statistics – may seem substantial, but it is still relatively modest when tasked with regressing multiple parameters simultaneously.

Despite these findings, DNNs have shown efficacy in enhancing the performance of other neural networks, particularly through the prediction of residuals using summary statistics. DNN might not be the optimal choice for estimating parameters from summary statistics alone, but they can be valuable in ensemble learning strategies.

N. Meta Information of the Selected Empirical Trees

Family	Tree	Ntip
Amphibia	Caecilidae	31
Amphibia	Hynobiidae	46
Amphibia	Salamandridae	42
Amphibia	Plethodontidae	278
Amphibia	Pipidae	23
Amphibia	Eleutherodactylidae	145
Amphibia	Ranidae	218
Bird	Tyrannidae	419
Bird	Thraupidae	370
Bird	Psittacidae	330
Bird	Trochilidae	334
Bird	Columbidae	306
Bird	Furnariidae	302
Bird	Muscicapidae	279
Bird	Accipitridae	242
Bird	Picidae	223
Bird	Thamnophilidae	219
Bird	Fringillidae	194
Bird	Strigidae	191
Bird	Turdidae	170
Bird	Meliphagidae	177
Bird	Phasianidae	176
Bird	Emberizidae	163
Bird	Anatidae	157
Bird	Cisticolidae	142
Bird	Pycnonotidae	124
Bird	Rallidae	125
Bird	Cuculidae	138
Bird	Estrildidae	140
Bird	Nectariniidae	127
Bird	Leiothrichidae	127
Bird	Corvidae	120
Bird	Zosteropidae	120
Bird	Sturnidae	109
Bird	Parulidae	109
Bird	Ploceidae	108
Bird	Icteridae	102
Bird	Apodidae	99
Bird	Laridae	99
Bird	Alaudidae	91
Bird	Monarchidae	87

Bird	Scolopacidae	89
Bird	Caprimulgidae	88
Bird	Alcedinidae	91
Bird	Campephagidae	80
Bird	Procellariidae	81
Bird	Hirundinidae	83
Bird	Troglodytidae	79
Bird	Phylloscopidae	71
Bird	Ardeidae	61
Bird	Pellorneidae	66
Bird	Sylviidae	62
Bird	Cardinalidae	68
Bird	Charadriidae	64
Bird	Falconidae	64
Bird	Motacillidae	62
Bird	Acanthizidae	63
Bird	Cotingidae	65
Bird	Vireonidae	58
Bird	Acrocephalidae	52
Bird	Bucerotidae	55
Bird	Paridae	53
Bird	Pachycephalidae	50
Bird	Locustellidae	53
Bird	Rhinocryptidae	53
Bird	Timaliidae	55
Bird	Cracidae	50
Bird	Pipridae	52
Bird	Grallariidae	49
Bird	Malaconotidae	46
Bird	Passeridae	48
Bird	Rhipiduridae	42
Bird	Dicaeidae	45
Bird	Tinamidae	47
Bird	Petroicidae	44
Bird	Ramphastidae	35
Bird	Tityridae	41
Bird	Trogonidae	42
Bird	Lybiidae	41
Bird	Paradisaeidae	40
Bird	Phalacrocoracidae	33
Bird	Bucconidae	35
Bird	Threskiornithidae	34
Bird	Mimidae	34
Bird	Odontophoridae	34

Bird	Oriolidae	30
Bird	Laniidae	29
Bird	Pittidae	31
Bird	Platysteiridae	30
Bird	Cettiidae	32
Bird	Megalaimidae	28
Bird	Maluridae	27
Bird	Sittidae	24
Bird	Meropidae	26
Bird	Dicruridae	24
Bird	Otididae	25
Bird	Alcidae	23
Bird	Hydrobatidae	22
Bird	Musophagidae	23
Bird	Megapodiidae	21
Bird	Cacatuidae	21
Bird	Diomedeidae	21
Bird	Vangidae	21
CrocoTurtle	Crocodylia	25
CrocoTurtle	Testudines	233
Mammal	Vespertilionidae	386
Mammal	Soricidae	329
Mammal	Sciuridae	276
Mammal	Pteropodidae	174
Mammal	Phyllostomidae	150
Mammal	Bovidae	138
Mammal	Cercopithecidae	127
Mammal	Molossidae	98
Mammal	Didelphidae	84
Mammal	Hipposideridae	74
Mammal	Rhinolophidae	73
Mammal	Echimyidae	69
Mammal	Dasyuridae	63
Mammal	Mustelidae	59
Mammal	Heteromyidae	58
Mammal	Leporidae	58
Mammal	Macropodidae	56
Mammal	Nesomyidae	55
Mammal	Ctenomyidae	51
Mammal	Dipodidae	51
Mammal	Emballonuridae	49
Mammal	Cebidae	48
Mammal	Cervidae	45
Mammal	Felidae	40

Mammal	Talpidae	39
Mammal	Geomyidae	38
Mammal	Pitheciidae	37
Mammal	Canidae	34
Mammal	Delphinidae	34
Mammal	Viverridae	34
Mammal	Herpestidae	33
Mammal	Spalacidae	31
Mammal	Ochotonidae	28
Mammal	Gliridae	27
Mammal	Tenrecidae	25
Mammal	Atelidae	24
Mammal	Erinaceidae	22
Mammal	Phalangeridae	22
Squamate	Xantusiidae	26
Squamate	Gerrhosauridae	28
Squamate	Cordylidae	42
Squamate	Varanidae	53
Squamate	Chamaeleonidae	142
Squamate	Iguanidae	31
Squamate	Phrynosomatidae	114
Squamate	Pythonidae	26
Squamate	Viperidae	209

O. List of Summary Statistics

**Summary Statistics**	
Gamma	Area Per Pair (aPP)
Sackin	Average Leaf Depth (aLD)
Colless	I Statistic
Aldous' Beta Statistic	ewColless
Blum	Max Delta Width (maxDelW)
Crown Age	Maximum of Depth
Tree Height	Variance of Depth
Pigot's Rho	Maximum Width
Number of Lineages	Rogers
nLTT with Empty Tree	Total Cophenetic Distance
Phylogenetic Diversity	Symmetry Nodes
AvgLadder Index	Mean of Pairwise Distance (mpd)
Cherries	Variance of Pairwise Distance (vpd)
ILnumber	Phylogenetic Species Variability (psv)
Pitchforks	Mean Nearest Taxon Distance (mntd)
Stairs	J Statistic of Entropy
Stairs2	Rquartet Index
Laplacian Spectrum Asymmetry	Laplacian Spectrum Log Eigen
Laplacian Spectrum Peakedness	Laplacian Spectrum Eigengap
Number of Nodes	Wiener Index
B1	Max Betweenness
B2	Max Closeness
Diameter, Without Branch Lengths	Maximum Eigen Vector Value
Mean Branch Length	Variance of Branch Length
Mean External Branch Length	Variance of External Branch Length
Mean Internal Branch Length	Variance of Internal Branch Length
Number of Imbalancing Steps	J_One Statistic

Table 6. List of phylogenetic summary statistics used in neural network training

P. Computational Costs

In our experiments, generating 100,000 phylogenies took 3–8 hours, depending on the scenario (BD, DDD, or PBD). MLE on BD models ran in about 2 hours for all 100,000 trees (with parallelization), whereas fitting DDD models could take up to 24 hours per tree and often failed. Neural-network costs split into training and inference: training on 100,000 trees for 100 epochs with boosting approaches can exceed 48 hours on high-end GPUs (e.g., NVIDIA A100), though simpler architectures (DNN, CNN1D and LSTM) finish in ≈2 hours. Once trained, even our most complex network predicts parameters for 100,000 trees in ≈45 minutes (including up to 30 minutes of data loading and preprocessing) under GPU acceleration.
