# Supplementary figures and images for: Parameter Estimation from Phylogenetic Trees Using Neural Networks and Ensemble Learning
Source: Syst Biol. 2025 Sep 3;75(2):344–65. doi: 10.1093/sysbio/syaf060 (PMC13017624; doi:10.1093/sysbio/syaf060)

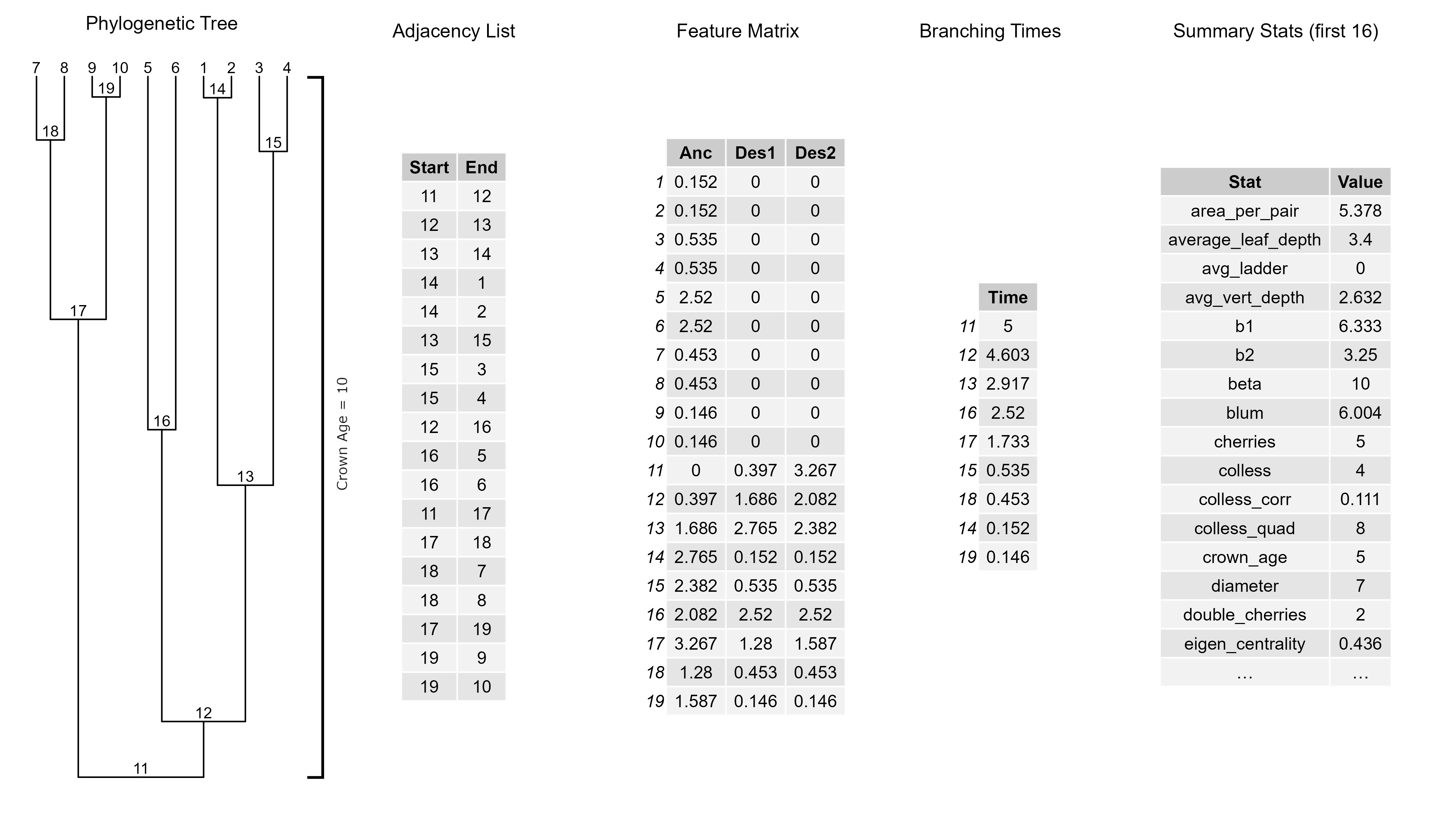

Supplement: syaf060_Supplemental_Files [file syaf060_supplemental_files.zip › figure12.png]

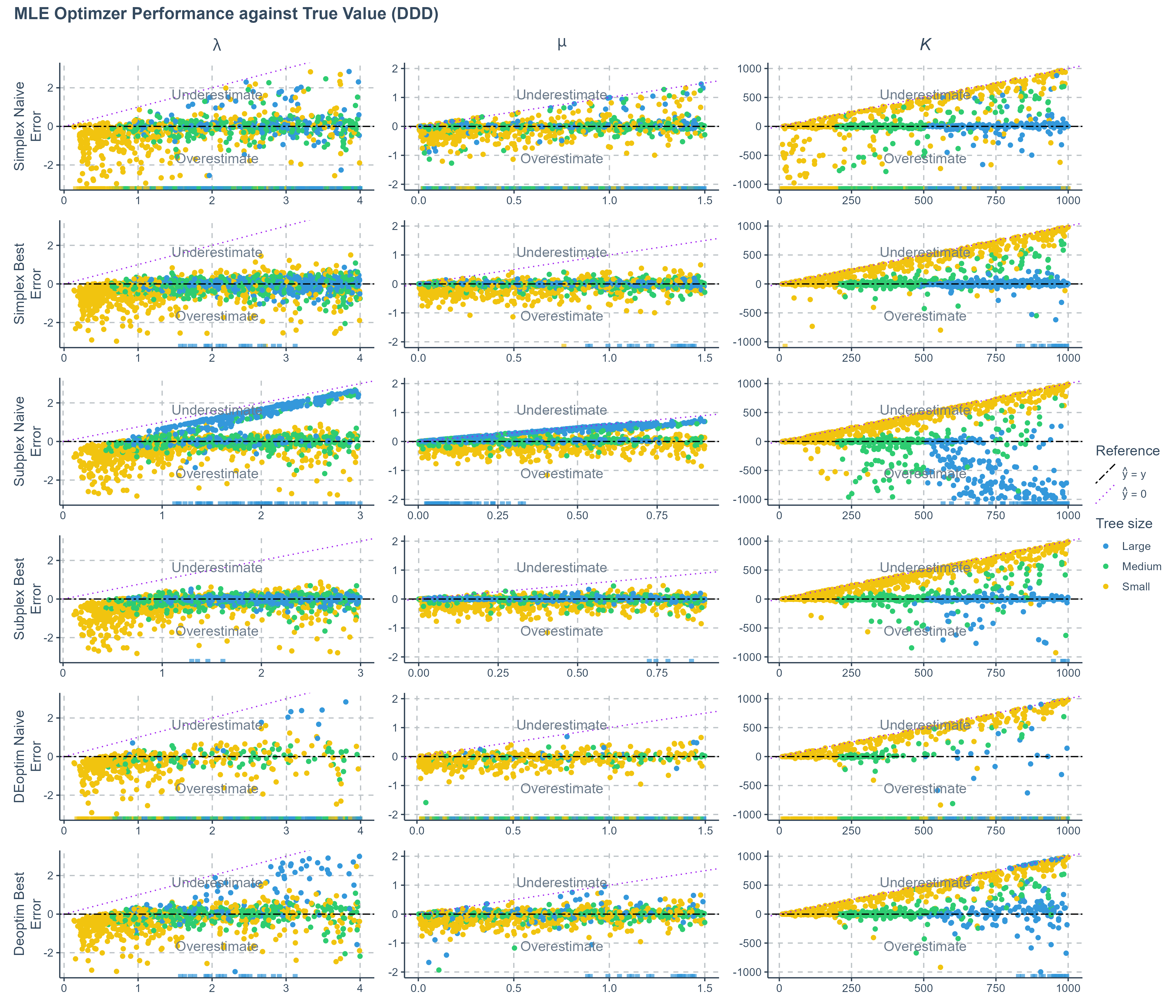

Supplement: syaf060_Supplemental_Files [file syaf060_supplemental_files.zip › figure13.png]

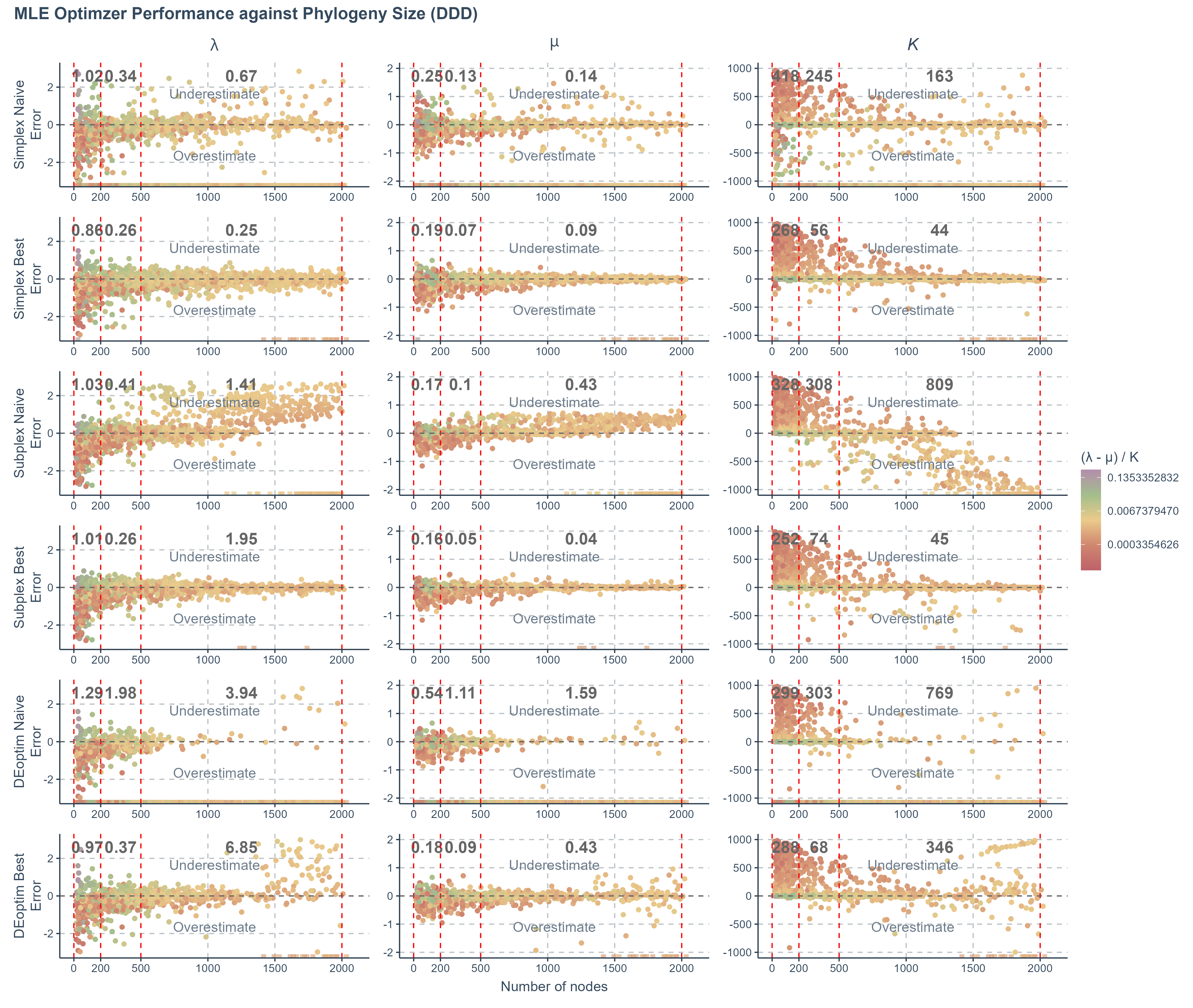

Supplement: syaf060_Supplemental_Files [file syaf060_supplemental_files.zip › figure14.png]

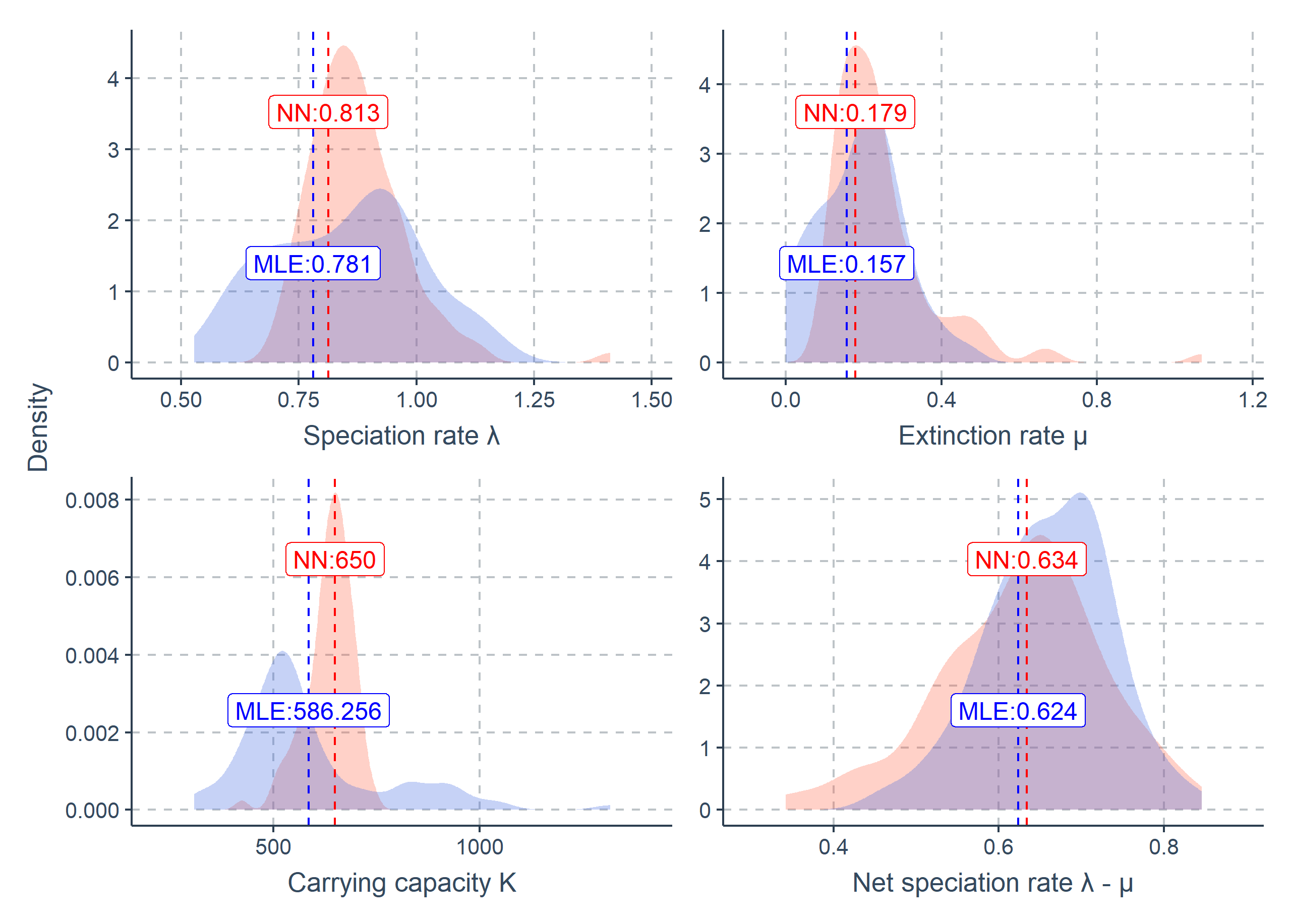

Supplement: syaf060_Supplemental_Files [file syaf060_supplemental_files.zip › figure15.png]

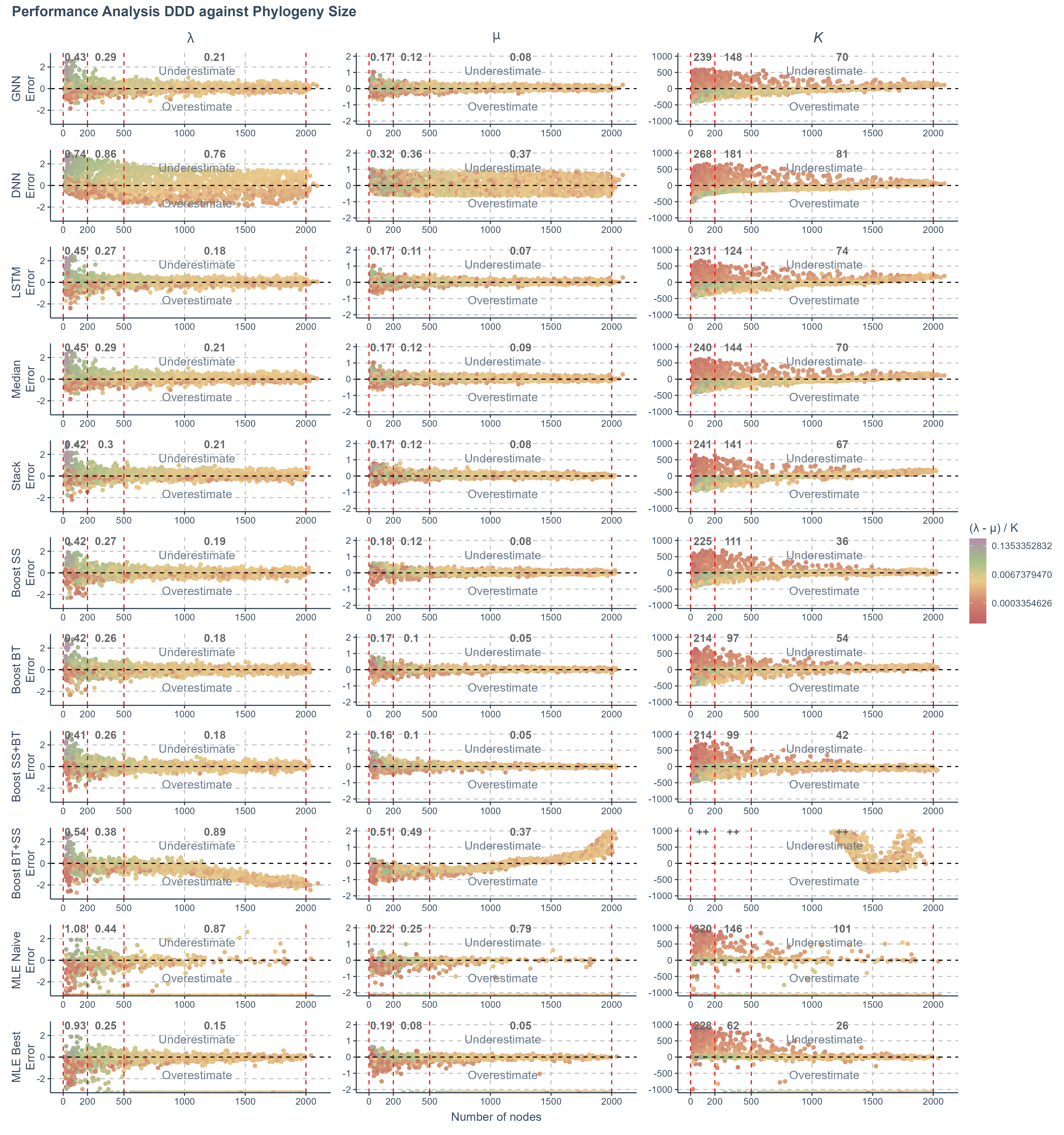

Supplement: syaf060_Supplemental_Files [file syaf060_supplemental_files.zip › figure16.png]

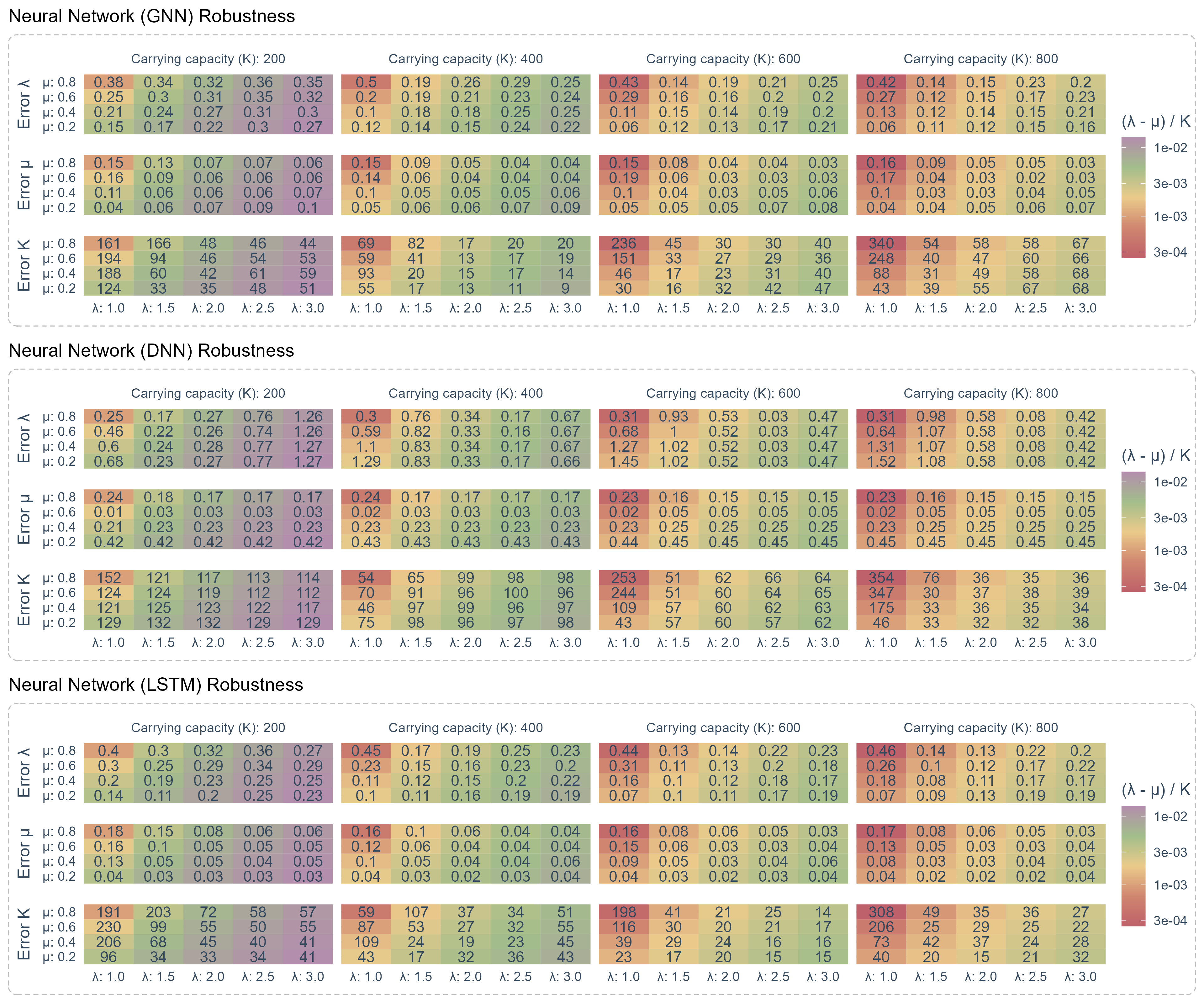

Supplement: syaf060_Supplemental_Files [file syaf060_supplemental_files.zip › figure17.png]

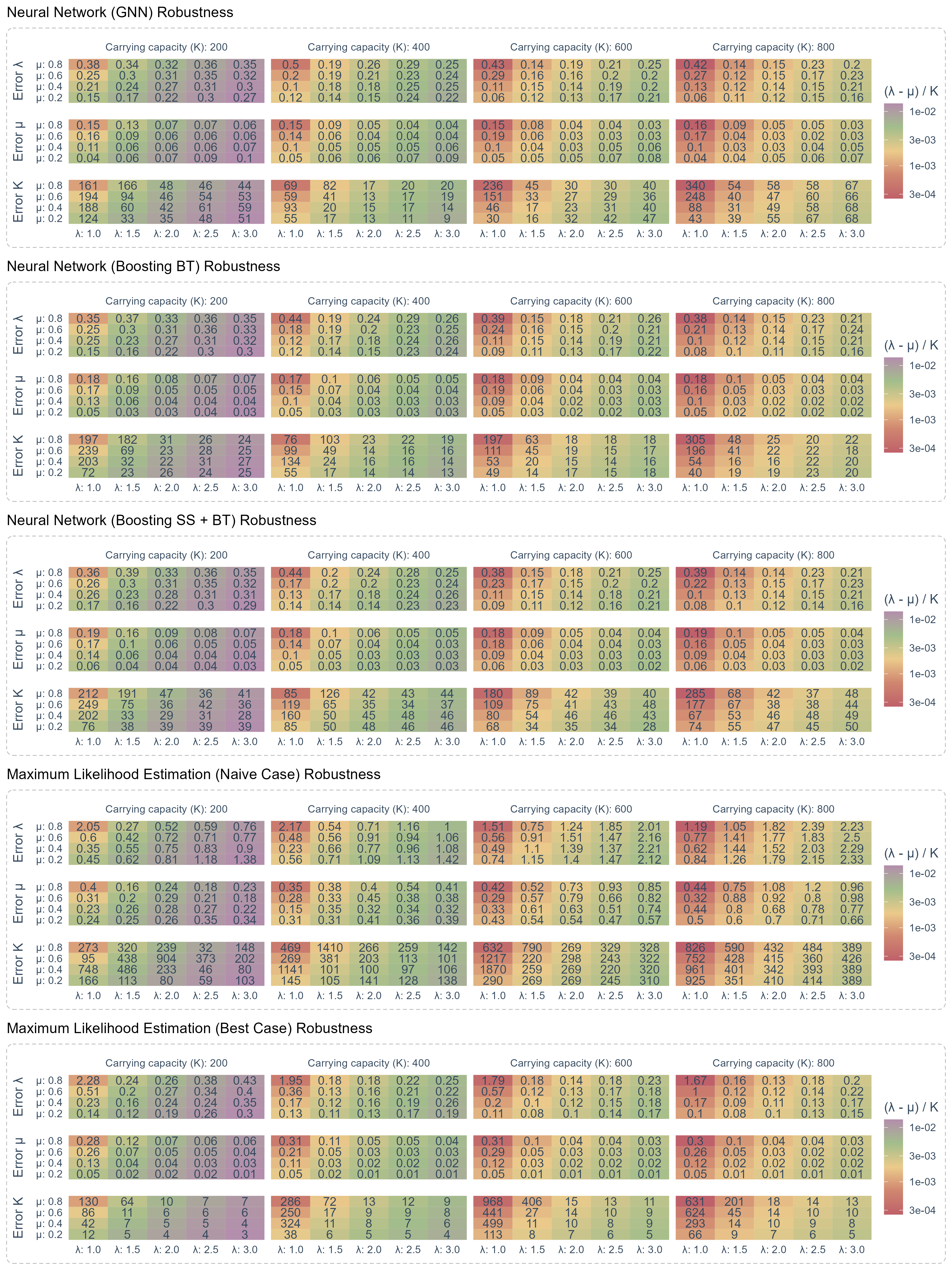

Supplement: syaf060_Supplemental_Files [file syaf060_supplemental_files.zip › figure18.png]

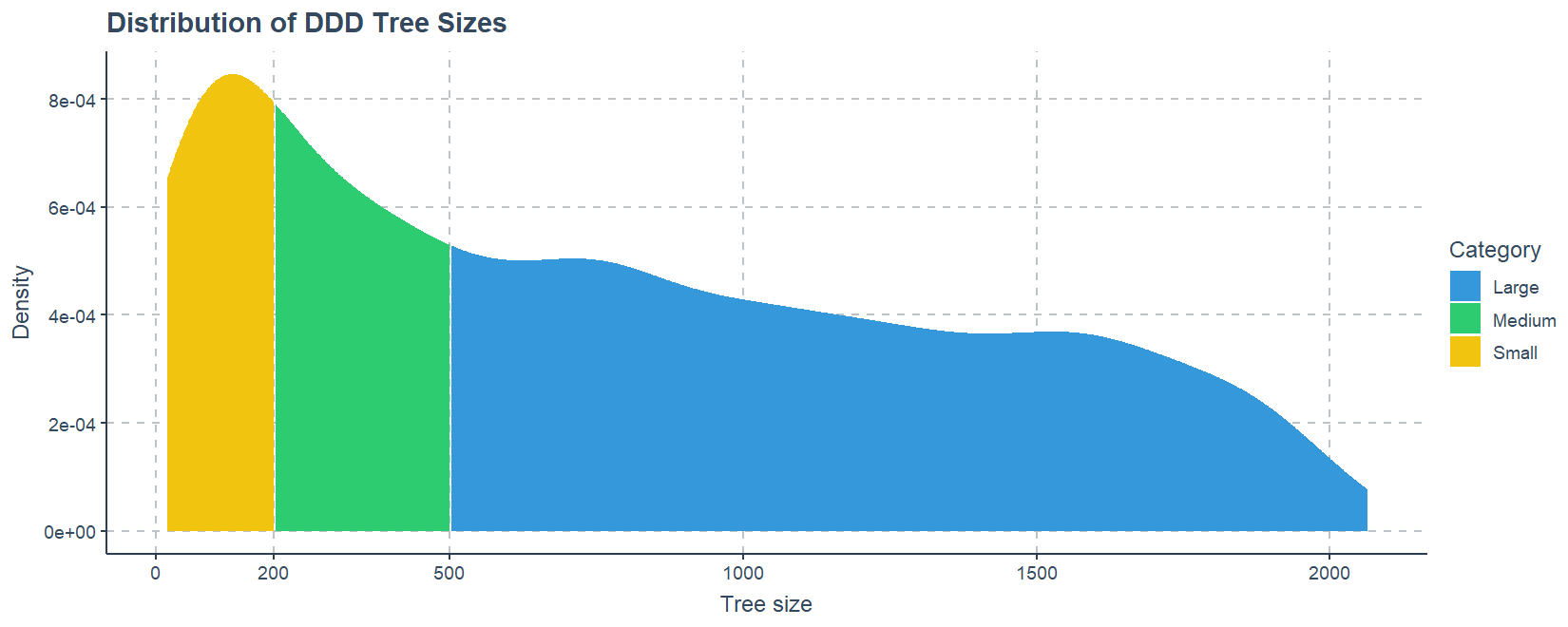

Supplement: syaf060_Supplemental_Files [file syaf060_supplemental_files.zip › figure19.png]

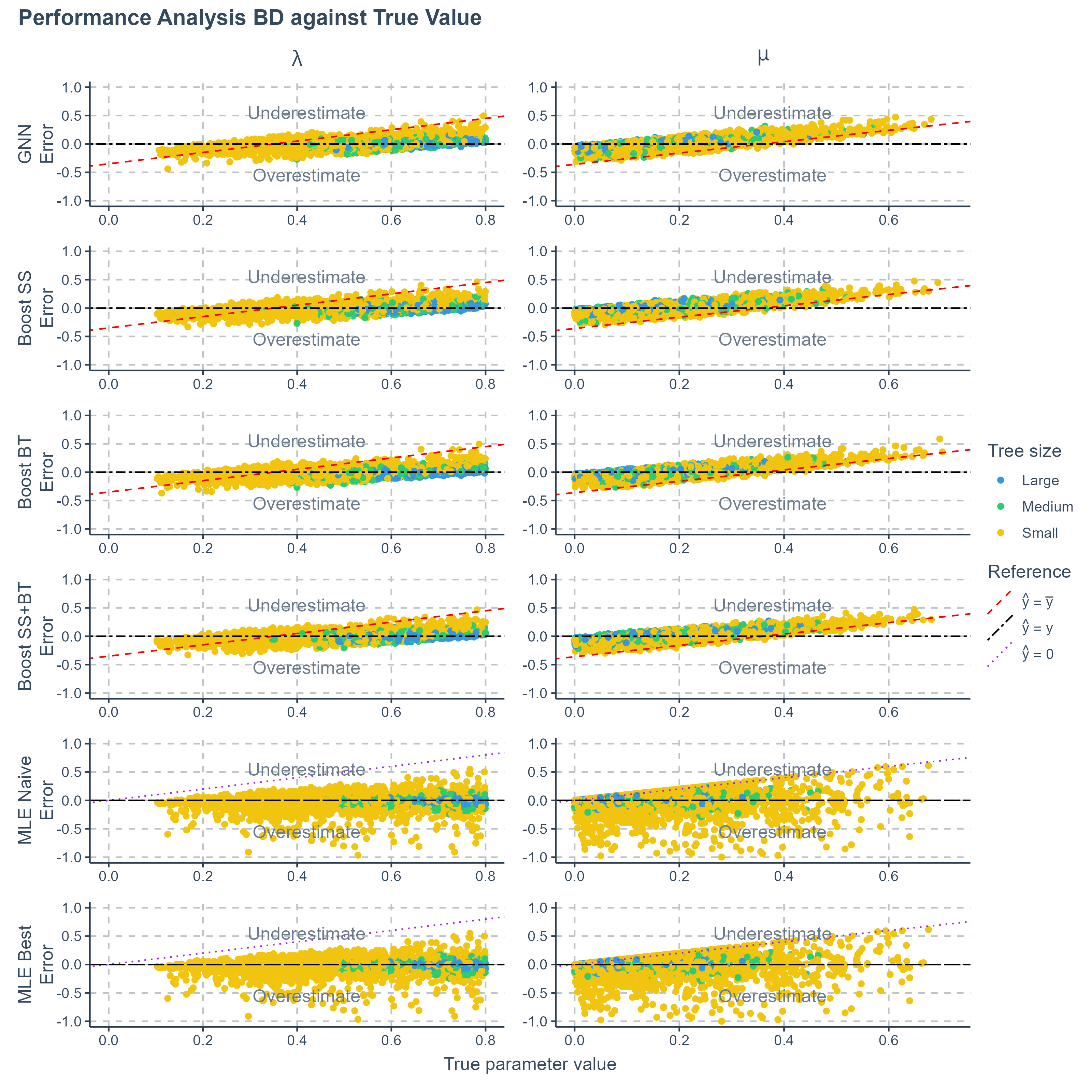

Supplement: syaf060_Supplemental_Files [file syaf060_supplemental_files.zip › figure20.png]

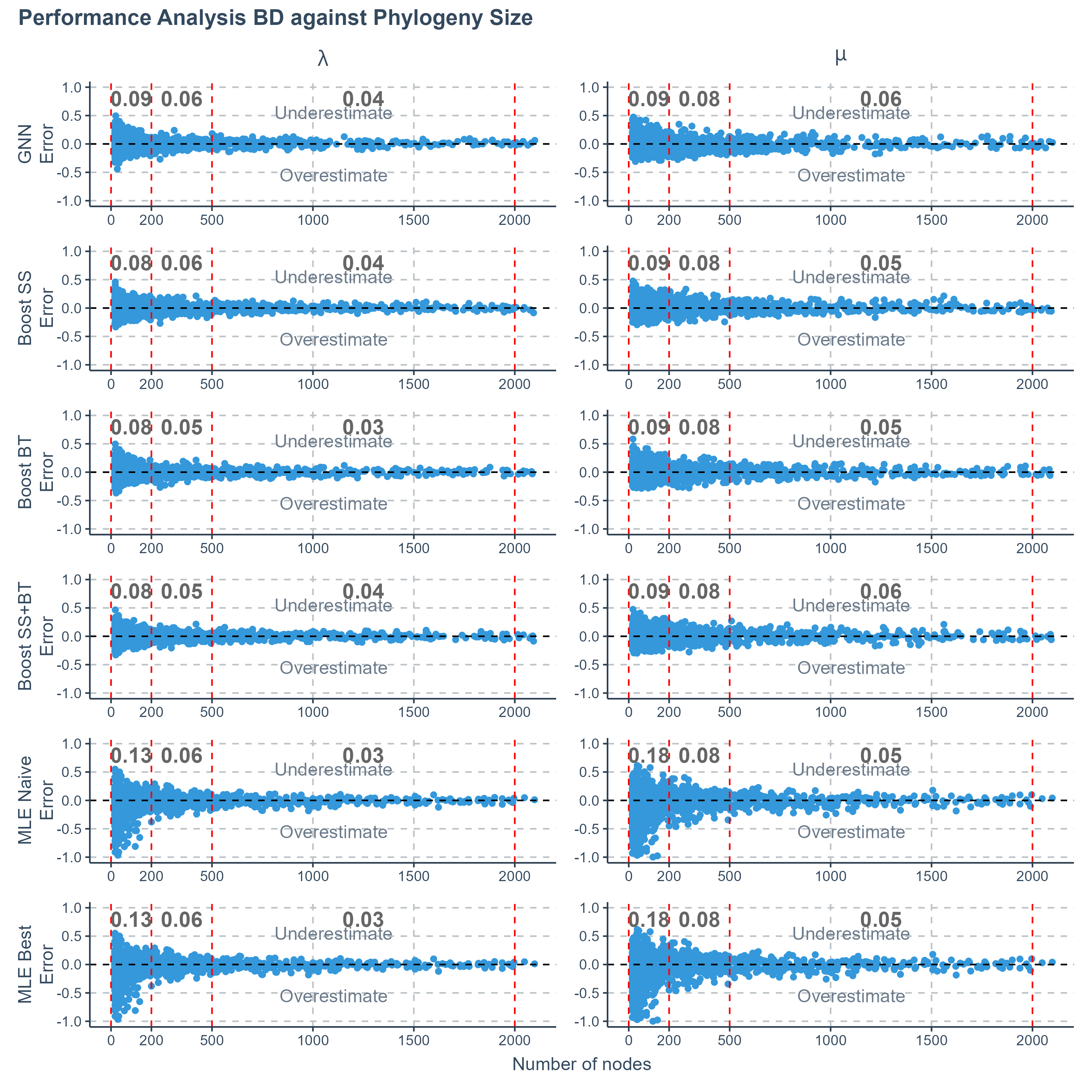

Supplement: syaf060_Supplemental_Files [file syaf060_supplemental_files.zip › figure21.png]

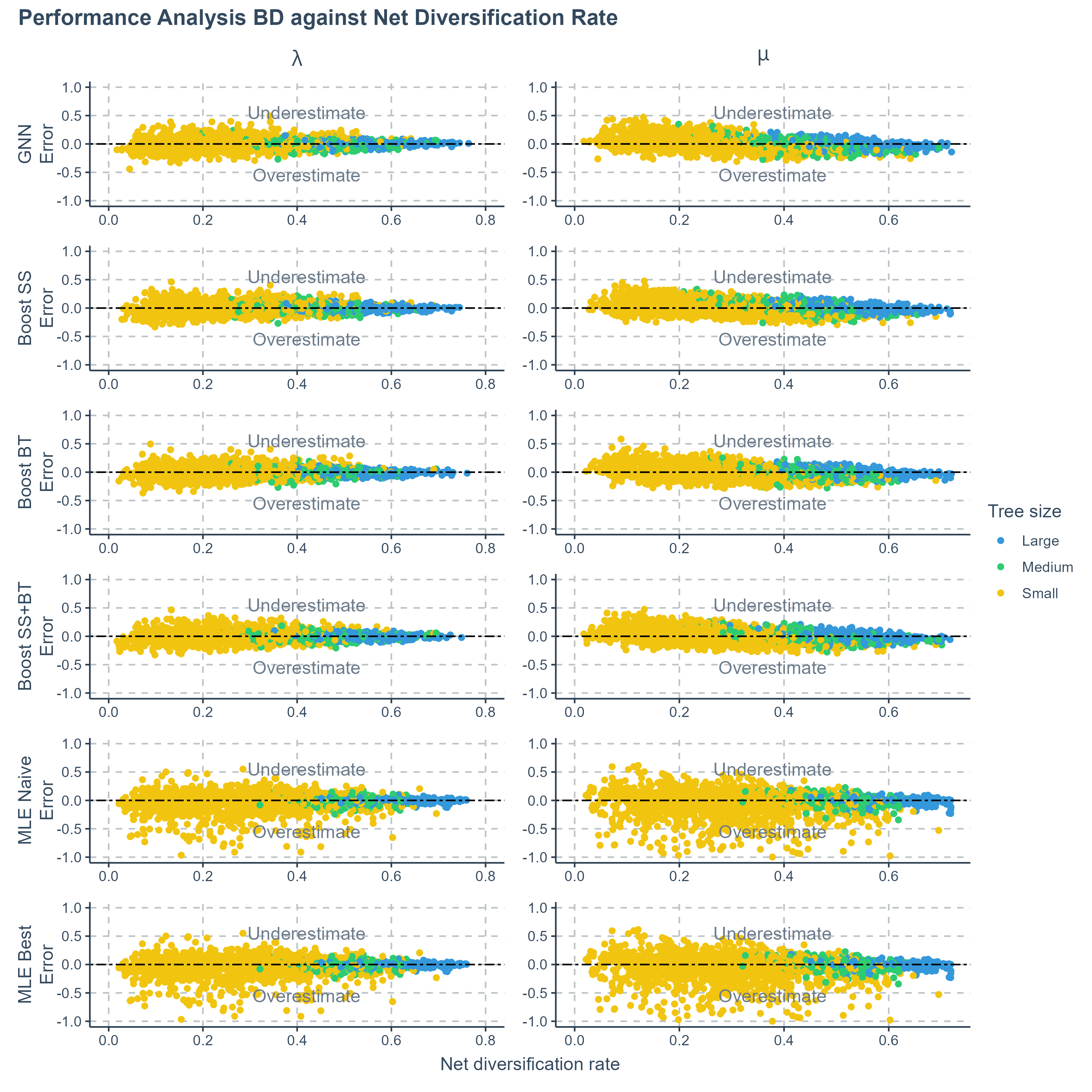

Supplement: syaf060_Supplemental_Files [file syaf060_supplemental_files.zip › figure22.png]

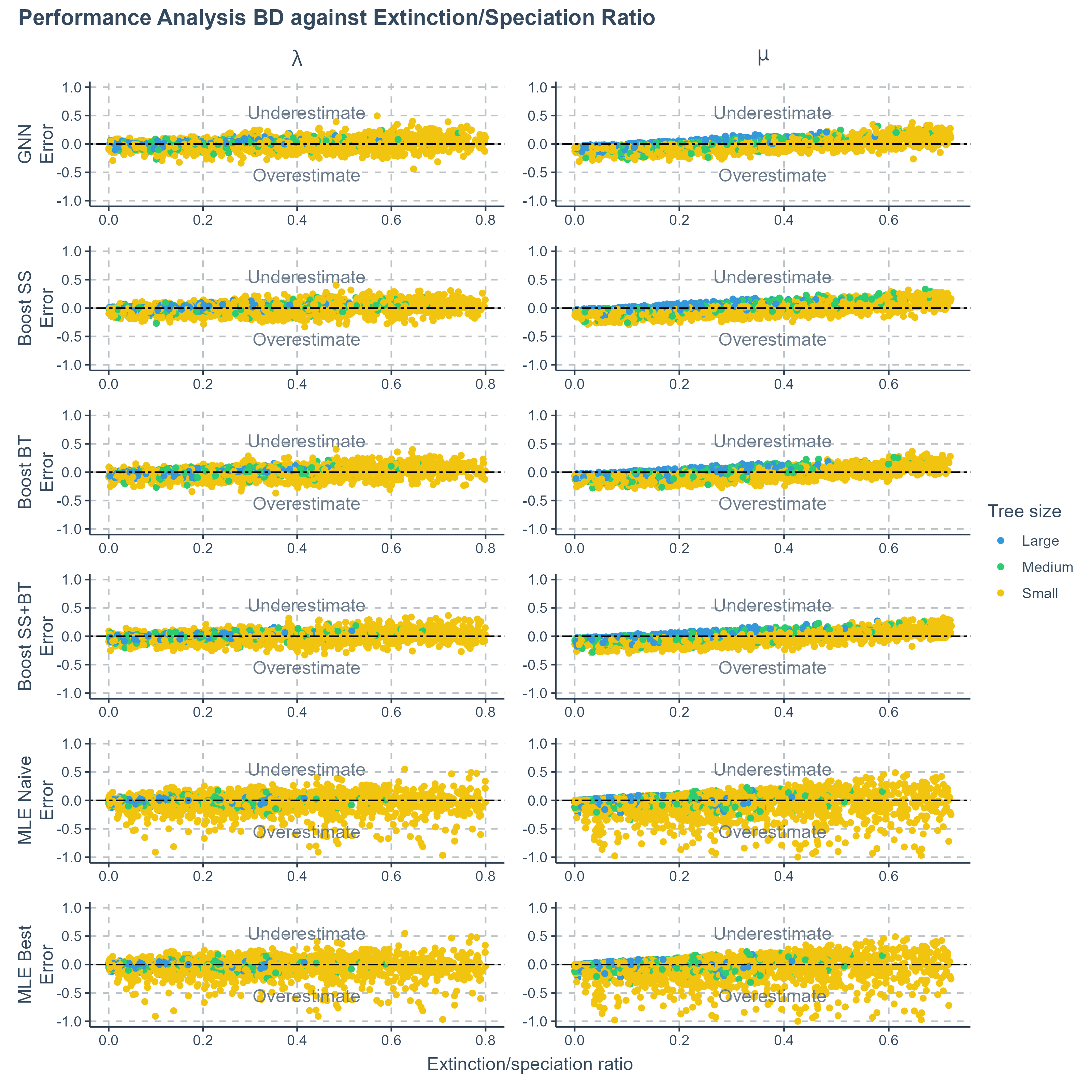

Supplement: syaf060_Supplemental_Files [file syaf060_supplemental_files.zip › figure23.png]

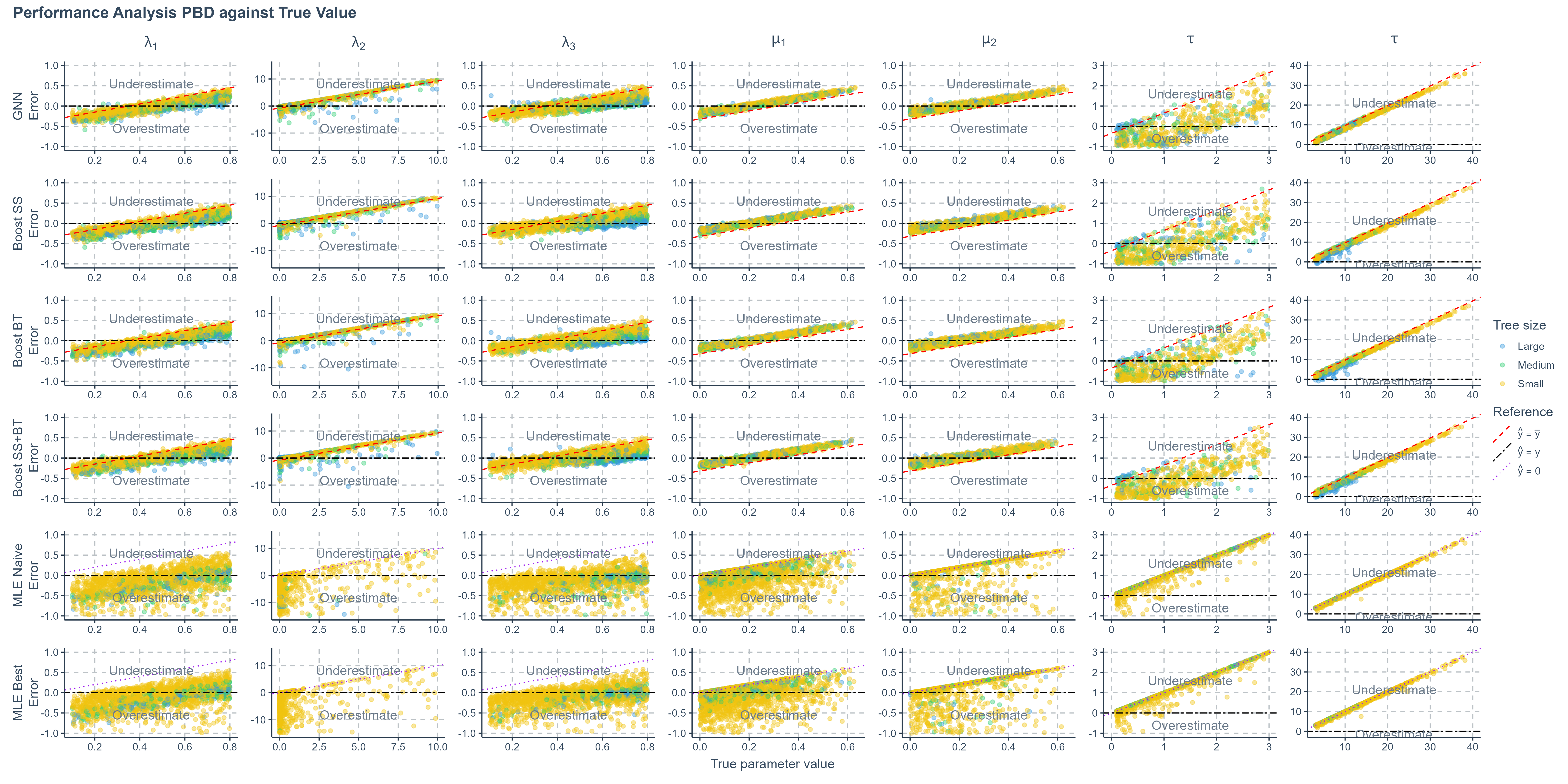

Supplement: syaf060_Supplemental_Files [file syaf060_supplemental_files.zip › figure24.png]

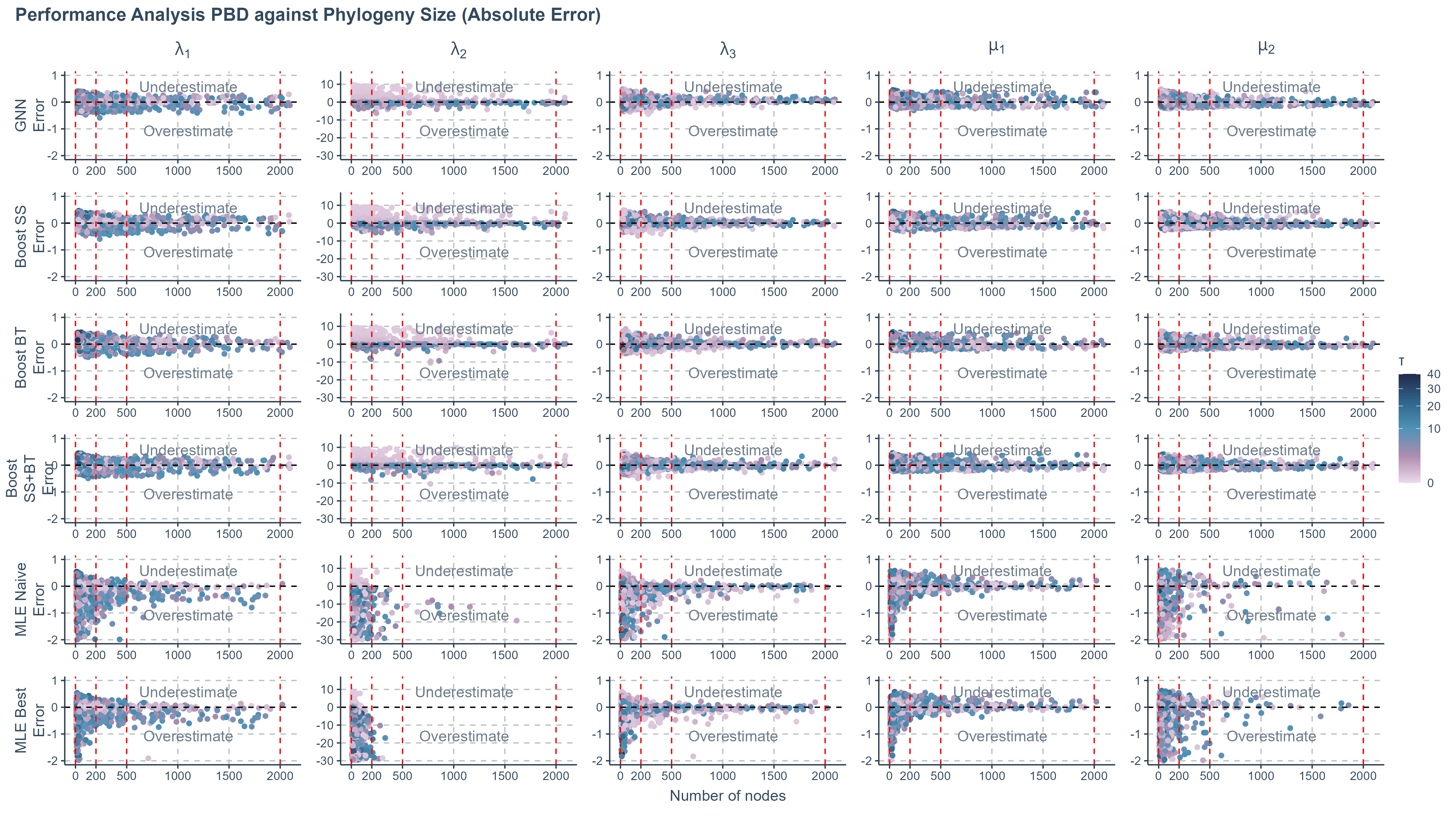

Supplement: syaf060_Supplemental_Files [file syaf060_supplemental_files.zip › figure25.png]

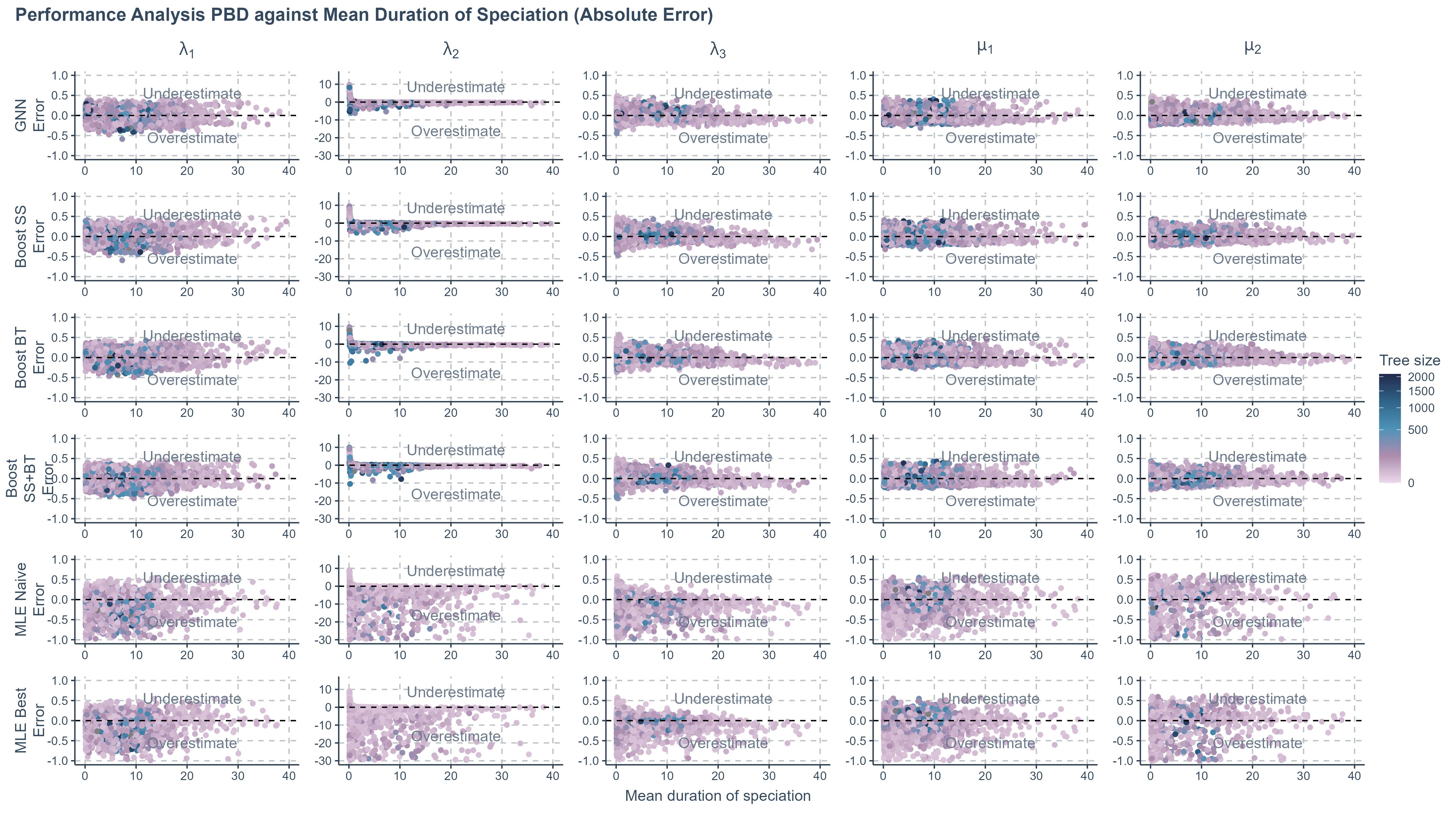

Supplement: syaf060_Supplemental_Files [file syaf060_supplemental_files.zip › figure26.png]

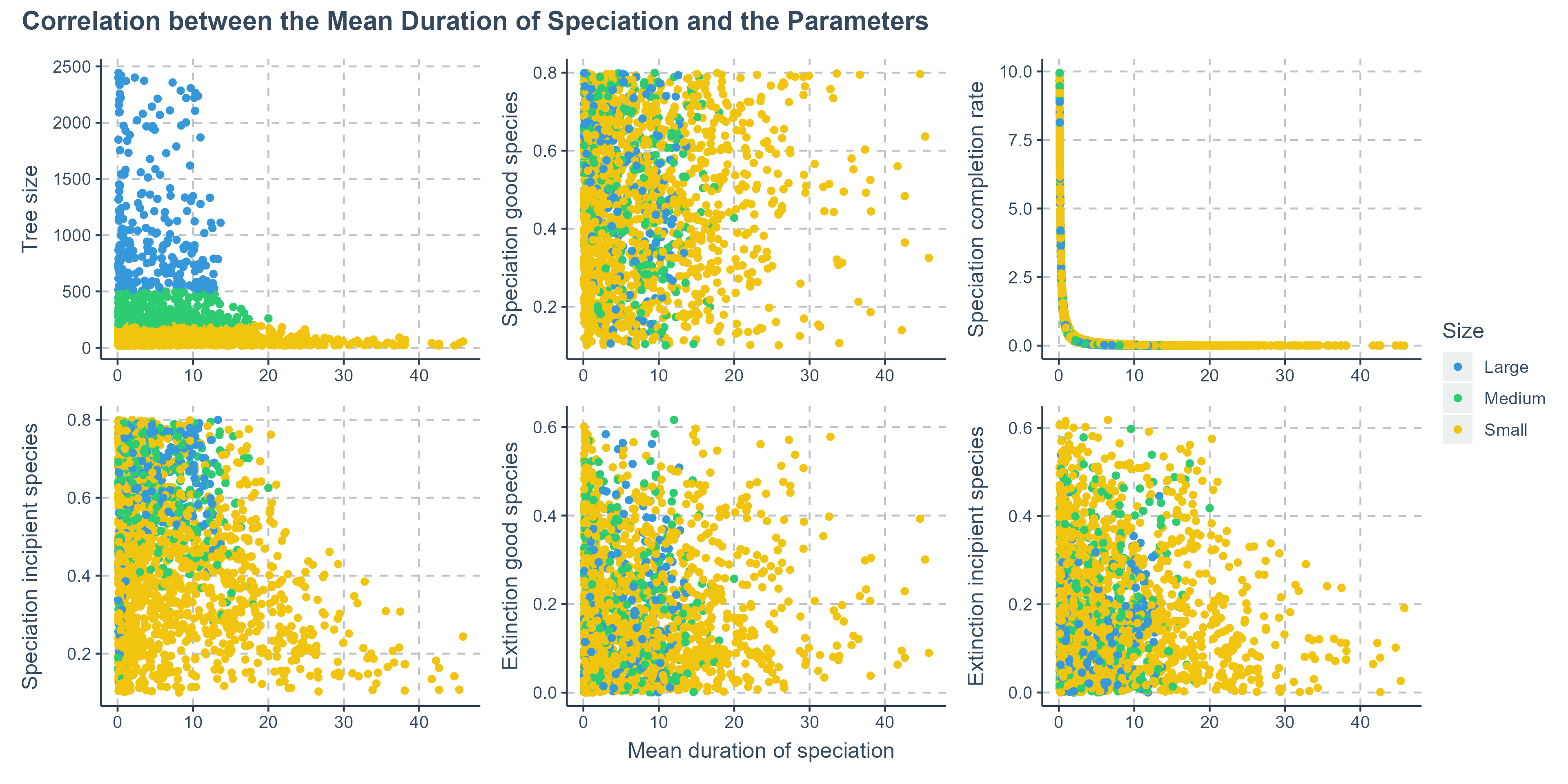

Supplement: syaf060_Supplemental_Files [file syaf060_supplemental_files.zip › figure27.png]

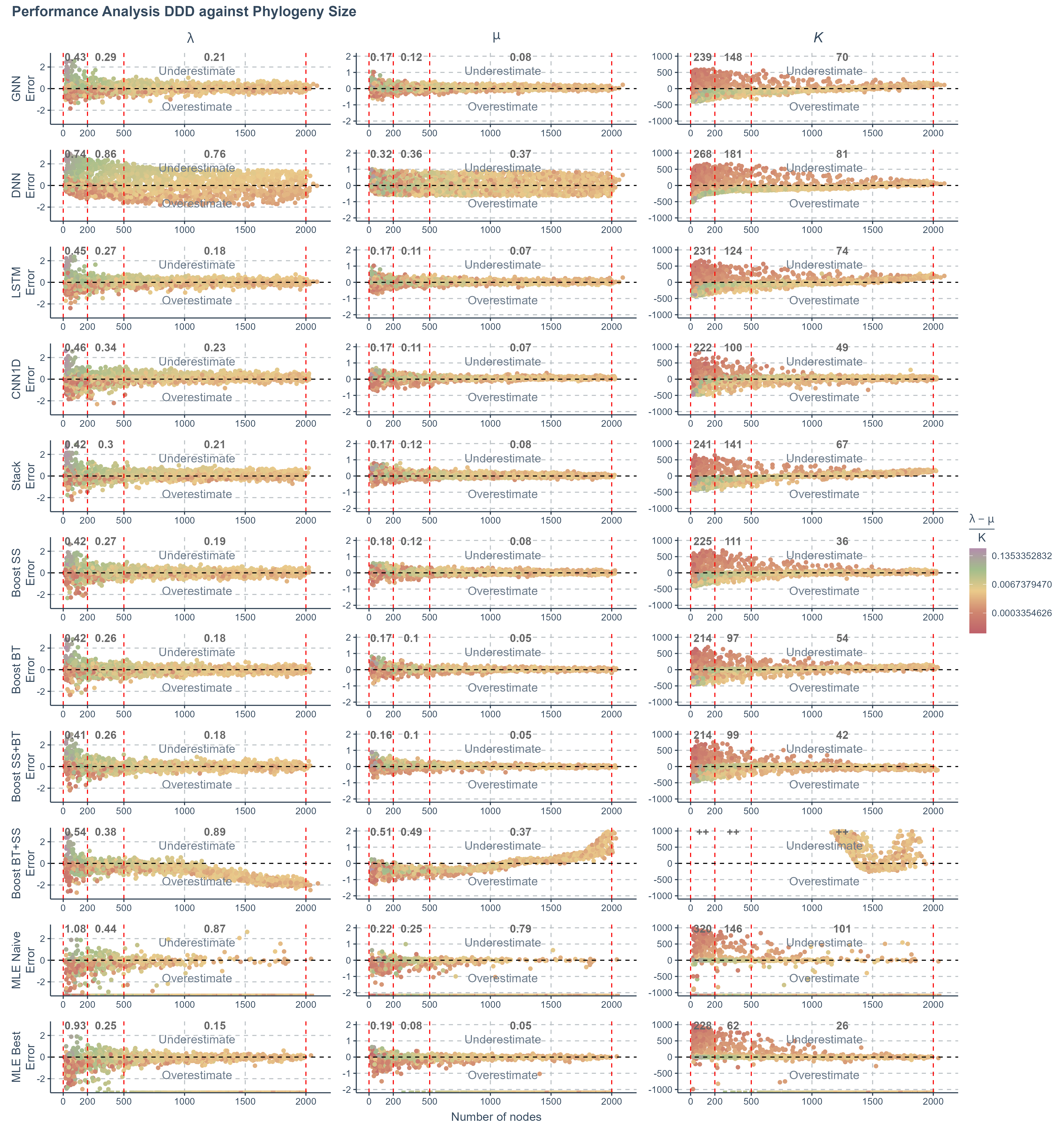

Supplement: syaf060_Supplemental_Files [file syaf060_supplemental_files.zip › figure28.png]

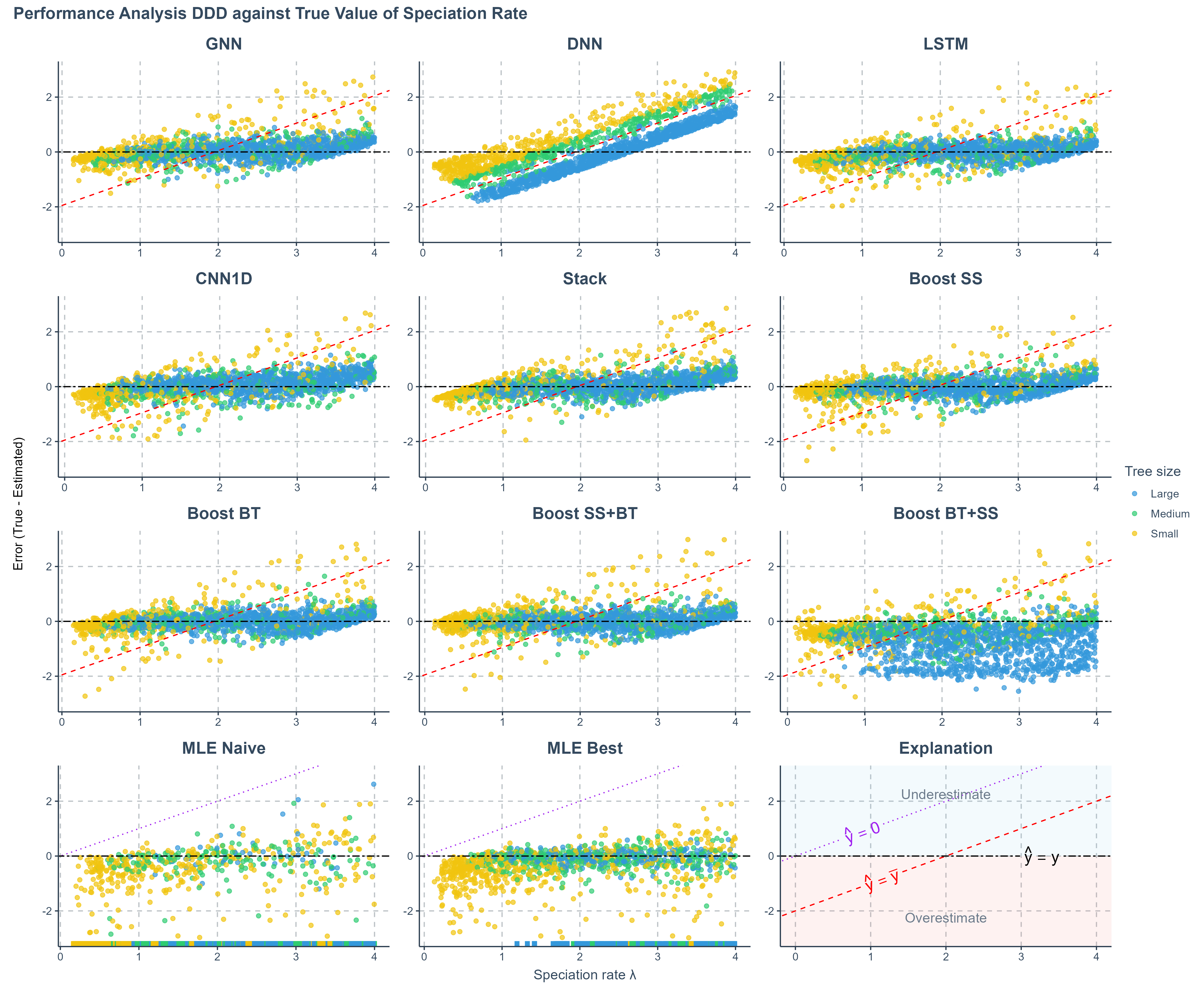

Supplement: syaf060_Supplemental_Files [file syaf060_supplemental_files.zip › figure29.png]

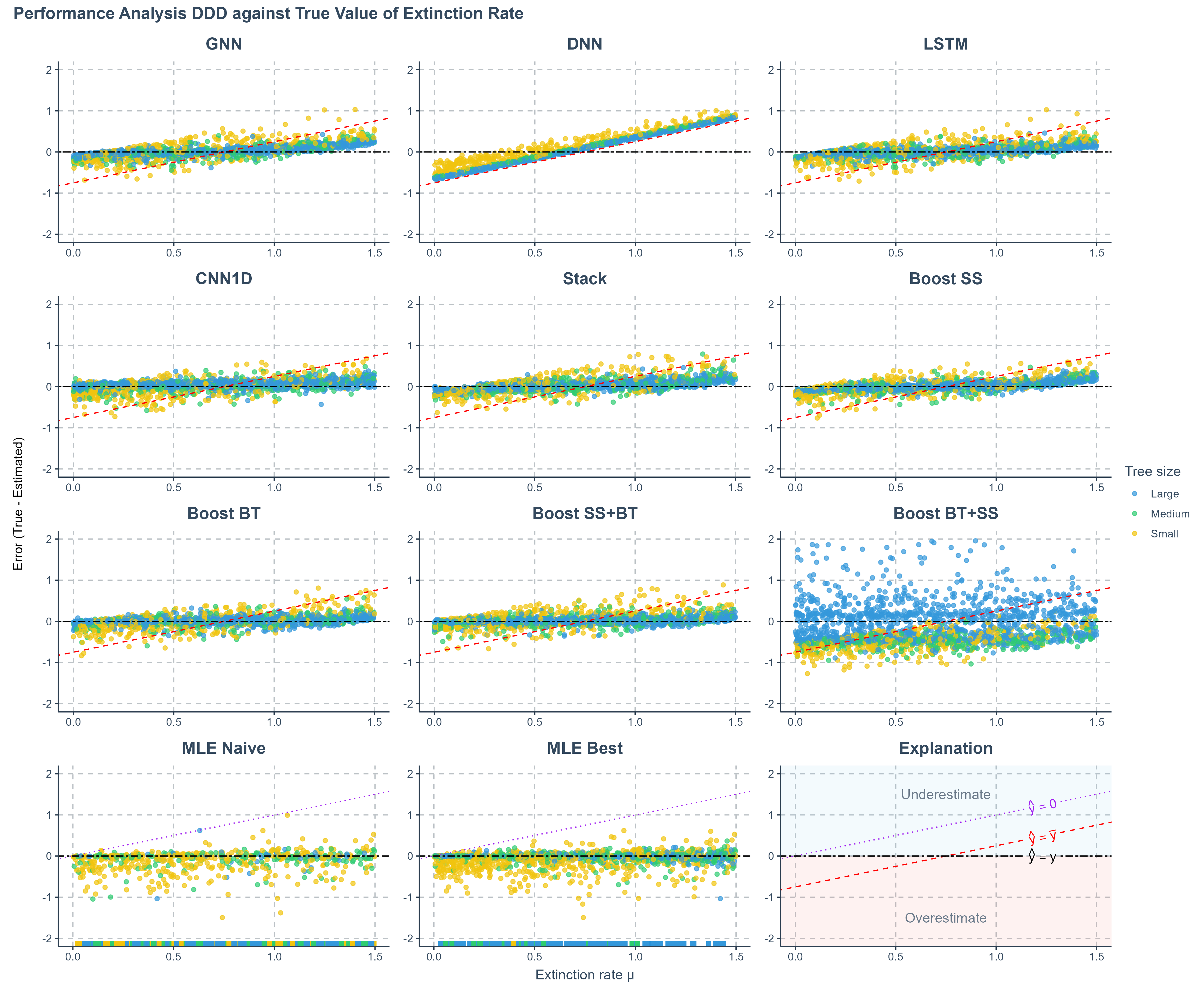

Supplement: syaf060_Supplemental_Files [file syaf060_supplemental_files.zip › figure30.png]

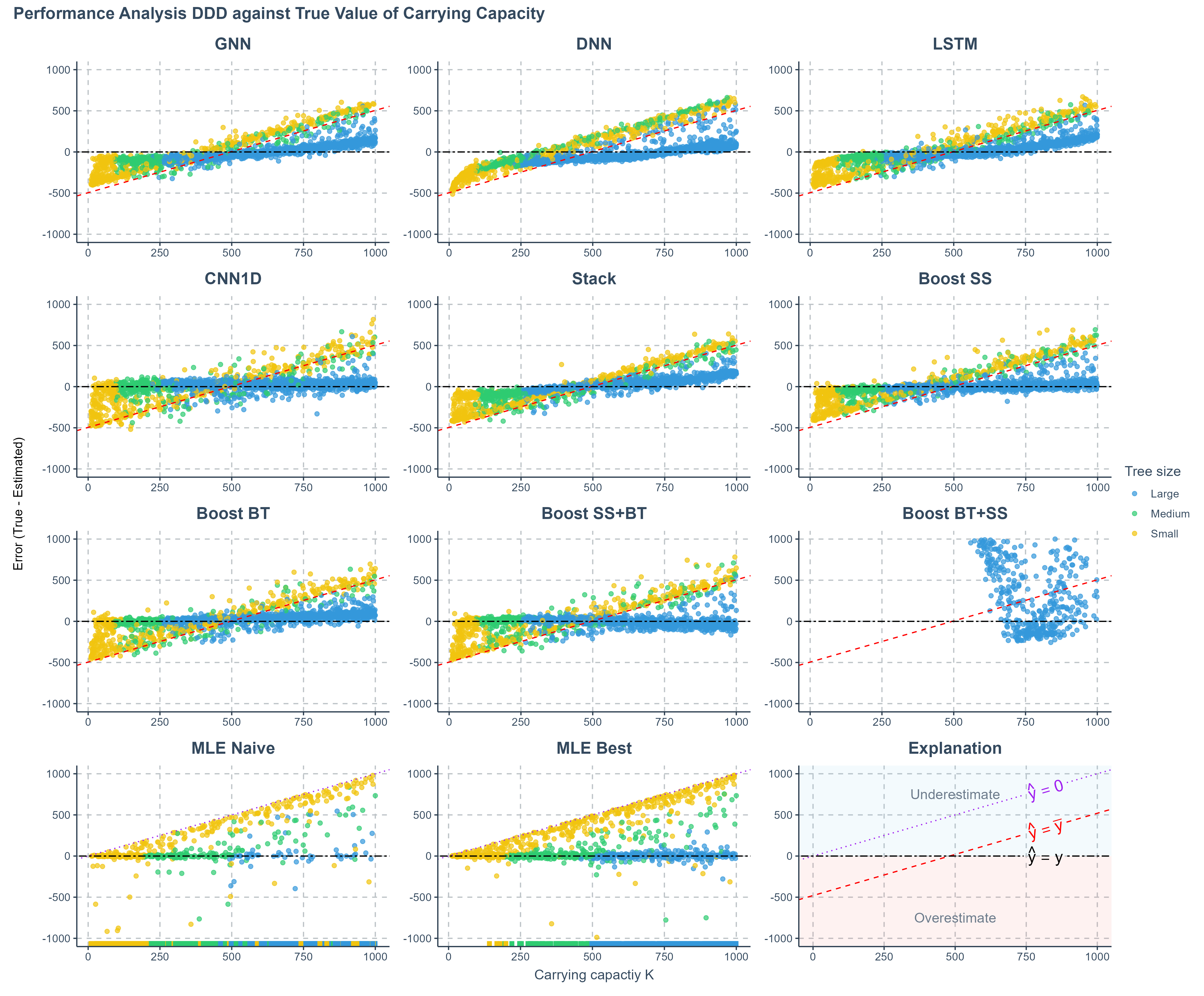

Supplement: syaf060_Supplemental_Files [file syaf060_supplemental_files.zip › figure31.png]

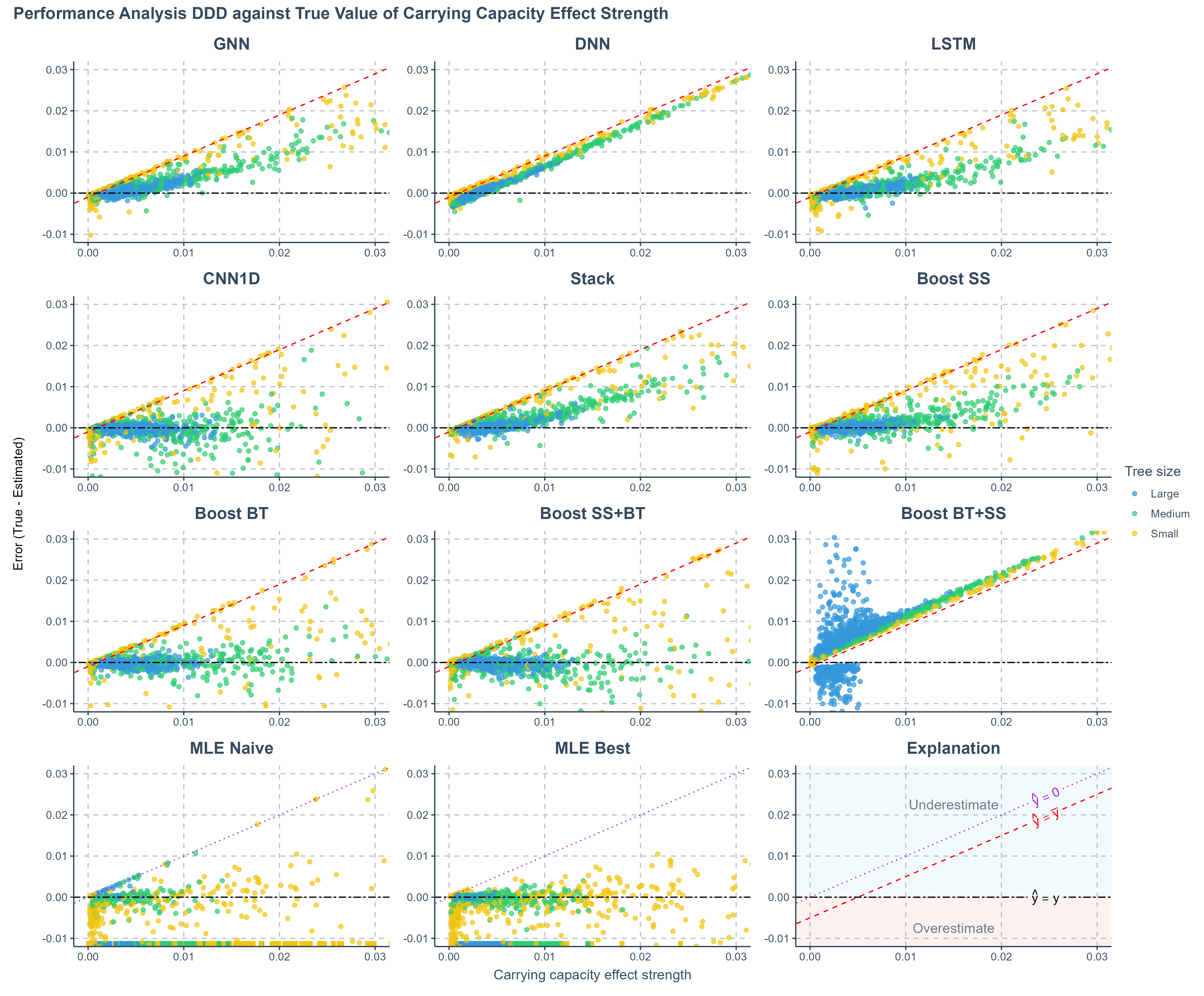

Supplement: syaf060_Supplemental_Files [file syaf060_supplemental_files.zip › figure32.png]

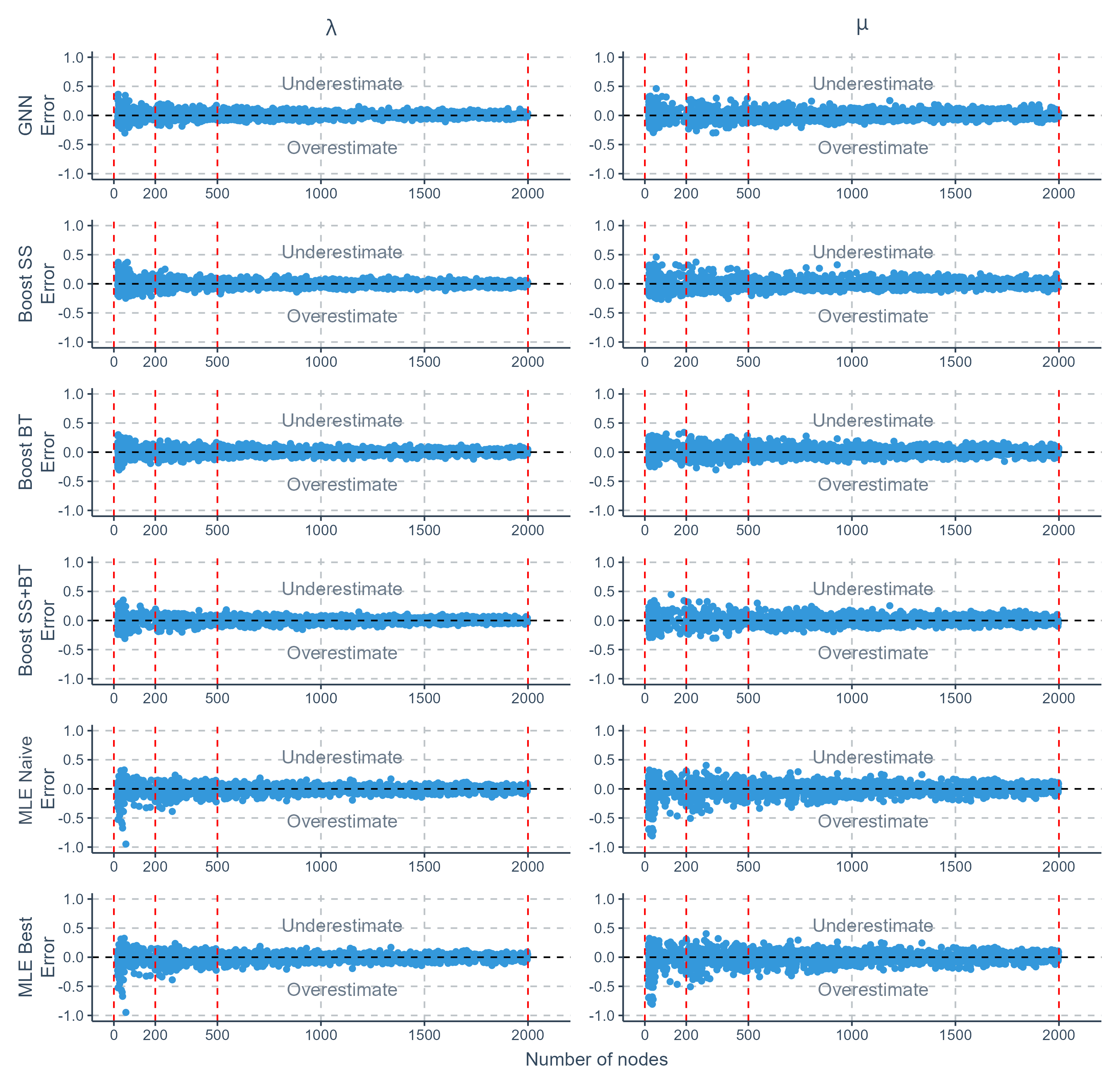

Supplement: syaf060_Supplemental_Files [file syaf060_supplemental_files.zip › figure33.png]

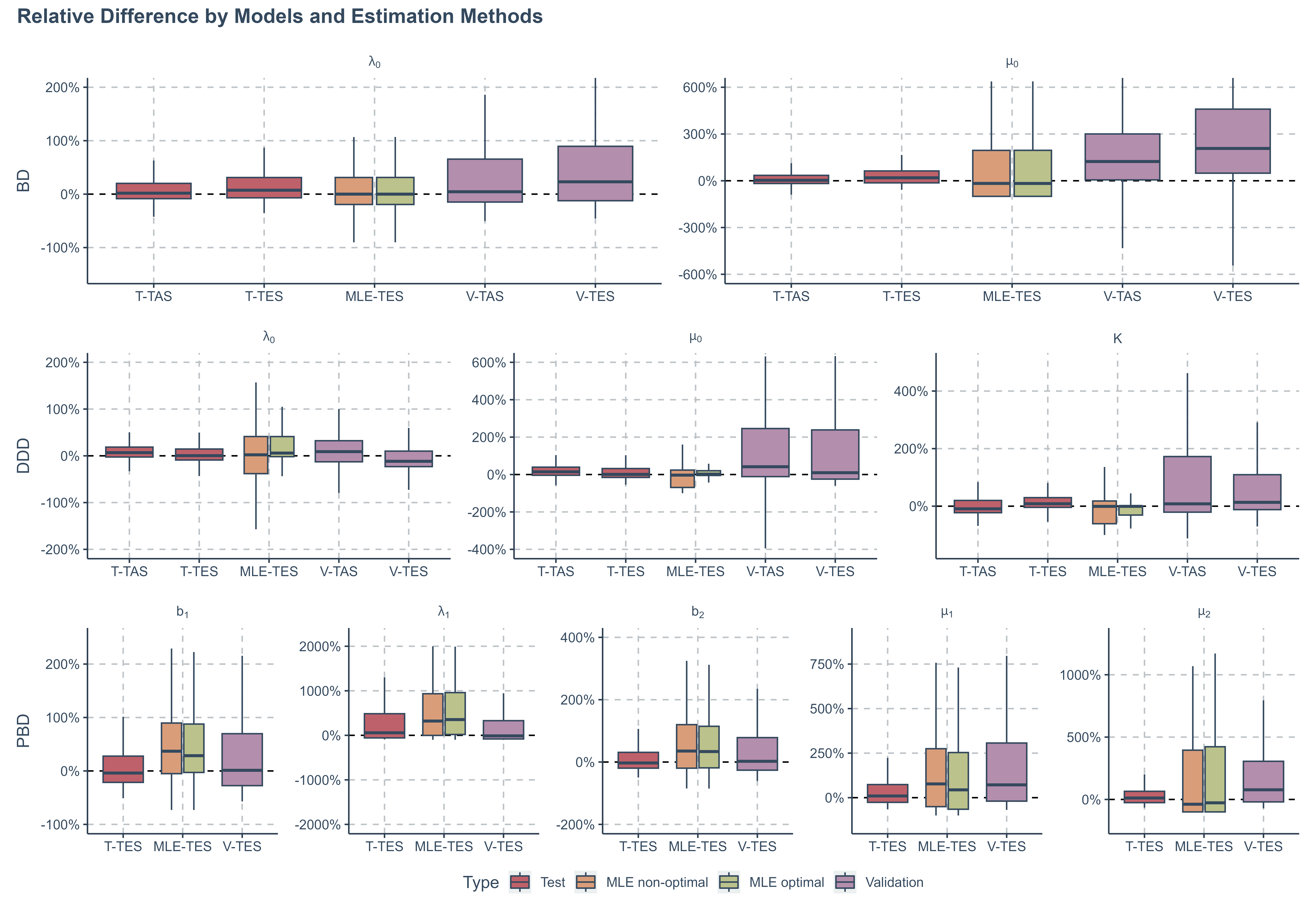

Supplement: syaf060_Supplemental_Files [file syaf060_supplemental_files.zip › figure34.png]

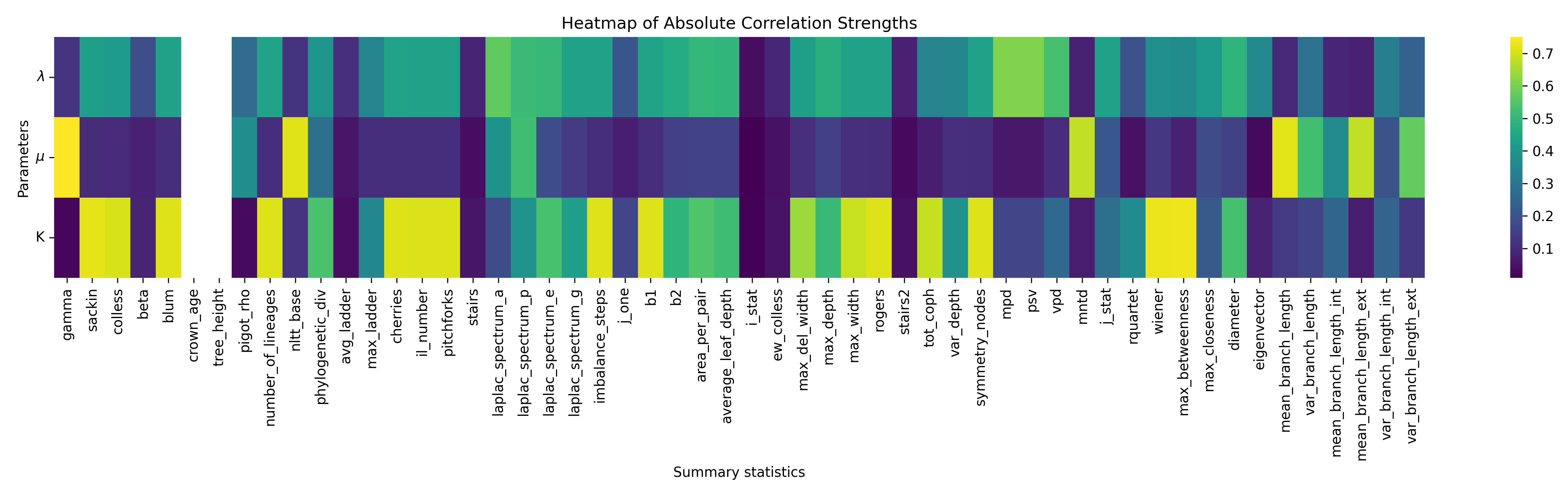

Supplement: syaf060_Supplemental_Files [file syaf060_supplemental_files.zip › figure35.png]
